# AI-Based Approaches for the Identification and Quantification of Traumatic Brain Injury in Computed Tomography Imaging: Systematic Review

**DOI:** 10.2196/87794

**Published:** 2026-09-10

**Authors:** Vu-Thu-Nguyet Pham, Irini Logothetis, Prasanthan Thaveenthiran, Simon Vajda, Rondhir Jithoo, Joseph Mathew, Kon Mouzakis

**Affiliations:** 1 Applied Artificial Intelligence Initiative, Deakin University, BC, Level 7/221 Burwood Hwy, Melbourne, Victoria, 3125, Australia, 61 487092912; 2 National Trauma Research Institute, The Alfred Hospital, Melbourne, Victoria, Australia; 3 Department of Neurosurgery, The Alfred Hospital, Melbourne, Victoria, Australia

**Keywords:** traumatic brain injury, AI, computed tomography, medical imaging, systematic review

## Abstract

**Background:**

Traumatic brain injury (TBI) is a leading cause of global disability and mortality, requiring timely diagnosis to prevent secondary injury. Manual computed tomographic (CT) evaluation often causes diagnostic delays, especially in smaller hospitals with limited radiological expertise. AI methods have been increasingly proposed to automate CT-based TBI assessment.

**Objectives:**

The aim of this review is to present a comprehensive review of AI techniques applied to CT scans for TBI assessment.

**Methods:**

This study presents a systematized review conducted in accordance with PRISMA (Preferred Reporting Items for Systematic Reviews and Meta-Analyses) 2020 reporting guidelines. Titles, abstracts, and full texts were screened against predefined eligibility criteria. Data on datasets, model architectures, preprocessing, and performance metrics were extracted using a structured form. Risk of bias was not formally assessed using a standardized tool. Instead, we qualitatively evaluated common sources of bias, such as dataset size, clinical evaluation, and external validation. Due to substantial heterogeneity in tasks, datasets, and outcome measures, we performed narrative synthesis only. We searched PubMed, Scopus, Web of Science, and IEEE Xplore from inception to December 31, 2025, for English-language peer-reviewed studies. We included original research papers that used AI or machine learning methods applied to human noncontrast head CT for TBI-related assessment. Studies had to report quantitative performance metrics. Nonoriginal papers, non-CT modalities, pediatric-only cohorts, and non-TBI applications were excluded. We restricted inclusion to English-language, peer-reviewed studies and did not perform a formal risk-of-bias or publication-bias assessment, which may overestimate the strength of the evidence.

**Results:**

By screening the 674 publications found, we identified 101 studies for evaluation. We grouped these 101 studies into 4 categories: binary TBI classification (TBI or non-TBI), hemorrhage detection (classification, localization, segmentation, and quantization), midline shift measurement, and increased intracranial pressure estimation. Most work focused on hemorrhage detection, with fewer studies on TBI severity classification, midline shift estimation, and increased intracranial pressure assessment. Reported performance was frequently high, but many studies relied on small, single-center datasets, limited annotation detail, and internal validation only. External validation and prospective clinical evaluation were rare.

**Conclusions:**

AI methods for CT-based TBI assessment demonstrate strong performance in retrospective evaluations under controlled conditions, particularly for hemorrhage detection and segmentation; however, the current evidence base is constrained by limited dataset diversity, incomplete reporting, and lack of clinical validation. Accordingly, these findings should be interpreted as evidence of methodological feasibility rather than clinical readiness.

## Introduction

### Overview

Traumatic brain injury (TBI), often referred to as the “silent epidemic” [1-3], affects an estimated 64‐74 million people globally each year [3]. In total, 176 American people die each day due to causes related to TBI as per the United States Centers for Disease Control and Prevention report “Multiple Cause of Death Data” released in 2020 [4]. TBI pathophysiology is generally categorized into 2 stages: primary and secondary injuries. Primary injuries are irreversible injuries that happen after the first physical trauma to the brain [5,6]. Secondary injuries develop over time due to complex molecular, metabolic, and inflammatory responses. These responses can lead to elevated intracranial pressure (ICP), cerebral vasospasm, ischemia, herniation, infarction, and midline shift (MLS) [7-9]. Unlike primary injuries, secondary injuries present a critical window of opportunity for intervention, referred to as the “golden hours.” Timely assessment and medical treatment during the golden hours following the trauma can significantly reduce or delay the progression and severity of these injuries [10,11]. Therefore, minimizing the time to treatment is essential in improving the outcomes of patients with TBI.

**Figure 1.**
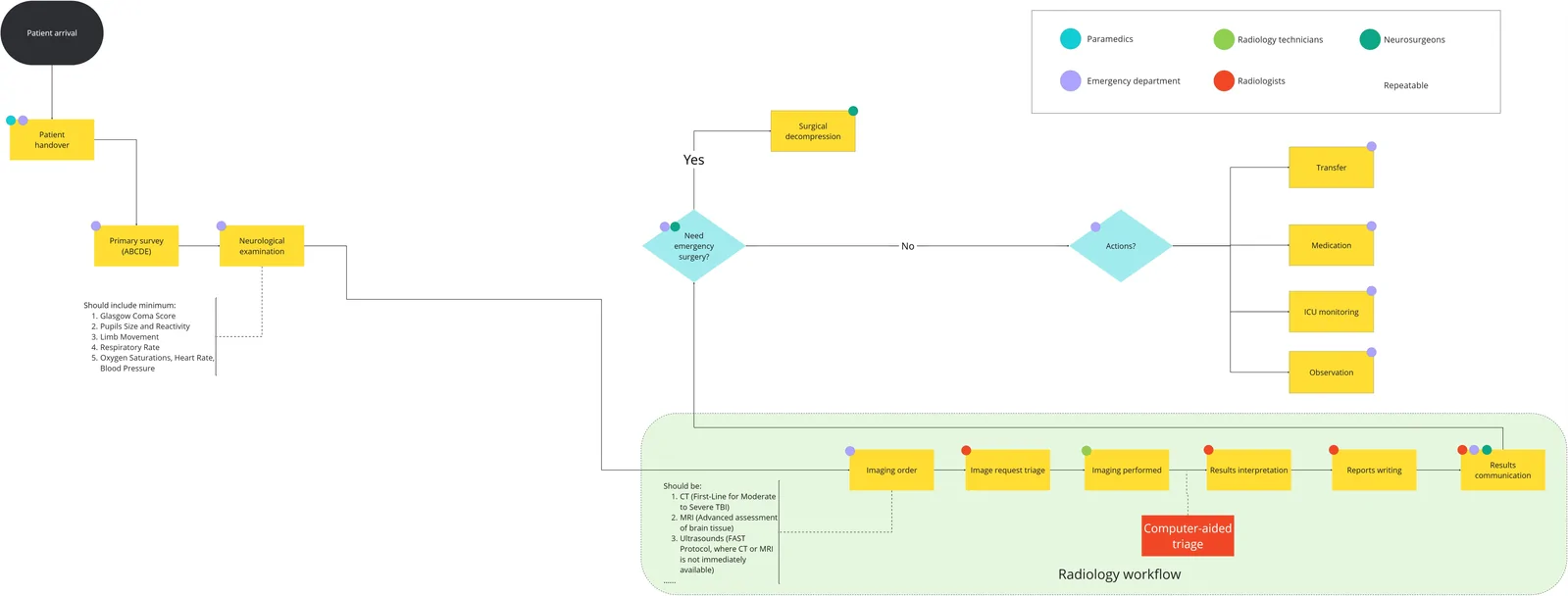
Standard clinical and radiological workflow for the assessment and management of patients with suspected TBI upon hospital arrival, highlighting the critical role and timing of CT imaging and interpretation. ABCDE: Airway, Breathing, Circulation, Disability, Exposure; CT: computed tomography; FAST: face, arm, speech, time; ICU: intensive care unit; MRI: magnetic resonance imaging; TBI: traumatic brain injury.

In recognition of this urgency, clinical management of suspected TBI begins immediately upon the patient’s arrival at the hospital. Figure 1 illustrates this clinical workflow. When a patient arrives at the hospital with suspected TBI, the emergency team first stabilizes the patient by performing the primary survey, which is designed to assess and treat life-threatening injuries in a timely fashion, using the systematic Airway, Breathing, Circulation, Disability, Exposure algorithm. The algorithms include an assessment of the patient’s neurological status, performed by evaluating consciousness using a Glasgow Coma Scale (GCS) and assessing the function of the central and peripheral nervous systems through motor strength, sensory function, reflexes, pupils’ examination, etc, which may provide signs of TBI. Based on these assessments, the emergency team determines the patient’s priority for care. Further imaging, including a brain scan as part of whole-body imaging, is requested when there are signs of TBI, such as low GCS scores, weaker motor strength, or unequal or unreactive pupils. Radiologists review images to confirm the diagnosis and provide detailed reports, enabling subspecialized teams to manage serious injuries.

Several imaging diagnostic tools are available for diagnosing TBI, depending on the care available, including computed tomography (CT) scans, magnetic resonance imaging (MRI) scans, positron emission tomography (PET) scans, ultrasound, and transcranial Doppler. These alternative tools have their own advantages and disadvantages. A comparison of these tools across the dimensions relevant to hospital settings is presented in Multimedia Appendix 1. It includes an evaluation on noninvasive, rapid results, cost, high-resolution anatomical imaging, accessibility, availability for emergency use, contraindications, and clinical utility. While MRI provides high sensitivity for detecting subtle injuries such as diffuse axonal injury or microhemorrhages, its use is often limited in acute settings due to longer scan times, higher costs, and reduced availability. Ultrasound and transcranial Doppler are noninvasive and useful for evaluating blood flow dynamics in brain vessels; however, they lack the spatial resolution to detect parenchymal injuries or structural abnormalities within the brain. Additionally, the accuracy of ultrasound or Doppler imaging can be subjective, as it depends on uncontrolled external factors, such as the technician’s expertise, patient movement, and the scanning technique used. Consequently, CT is the preferred first-line imaging modality for patients with trauma to diagnose TBI, as it is noninvasive, provides quick results, is widely available, and has few contraindications [12-14].

Despite its advantages, the reliance on CT imaging in TBI assessment presents its own set of challenges. First, in current practices, clinicians (in this paper, the term “clinicians” refers broadly to health care professionals involved in the TBI assessment, including general practitioners, radiologists, neurosurgeons, and other relevant specialists) typically perform assessments manually based on individual CT slices. Radiologists are inundated with medical images daily, with an estimated interpretation of hundreds of scans per shift [15]. In a busy hospital, this translates to thousands of scans requiring detailed analysis [16], creating an overwhelmingly heavy workload. Moreover, manual methods often fail to accurately and comprehensively quantify critical injury characteristics, such as hematoma size and volume, the degree of MLS, the extent of brain contusions, and indicators of diffuse axonal injury. Studies show that the average rate of diagnostic errors in radiology reports ranges from 3% to 5%, which translates to roughly 40 million imaging diagnostic errors each year [17]. Exacerbating this problem is an uneven distribution of skilled health care professionals and resource limitations, resulting in notable differences in care delivery and decision-making processes across various health care settings. For example, the Alfred Trauma Center in Australia has neurosurgeons available to review CT scans in real time via a dedicated viewing system. This enables the detection of severe TBI indicators in real time, allowing patients to be immediately transferred for surgical intervention. In contrast, smaller hospitals often face resource constraints, such as the unavailability of on-site radiologists. In such cases, nonspecialized or junior staff must review and assess the CT scans to identify severe cases that require immediate attention or necessitate the assistance of tertiary centers and/or escalation to a remote radiologist. This responsibility is particularly challenging when (1) other medical conditions mimic TBI symptoms or (2) the nonspecialized medical staff have limited experience with complex neuroimaging cases. Such scenarios are prone to inter- and intraobserver variability, leading to inconsistent interpretations and reduced diagnostic accuracy [17,18].

These challenges highlight the urgent need for automated, consistent solutions to support neuroimaging assessments, thereby alerting to concerning images, reducing interpretation time, and ensuring consistency in TBI assessments. Computerized tools could be transformative for smaller, rural hospitals to support health care workers when specialized neurosurgical and neurological care resources are scarce [19,20]. To address these issues, researchers are leveraging AI, particularly computer vision techniques, to automate the process of analyzing CT scans, thereby supporting medical staff in reducing the time required to identify severe TBI cases. AI-driven models can accurately detect and quantify critical findings, such as intracranial hemorrhages (ICHs) and MLSs [21]. In addition, these systems can support general practitioners and radiologists by reducing the time to identify and characterize TBIs in a scan from 15‐30 minutes per case [22] to seconds [23]. These advancements underscore the potential of integrating AI into the TBI assessment workflows in trauma units.

However, to develop an effective AI tool that supports clinicians in TBI assessment, the proposed AI framework must be tailored to address the challenges in integrating AI into clinical settings. This study aims to summarize existing AI approaches for detecting and quantifying CT-based abnormalities in TBI, highlighting their capabilities and limitations. Our research questions are as follows:

- RQ1: What are the types of problems in TBI assessment?
- RQ2: What data sources and data types are commonly used in AI for TBI assessment?
- RQ3: What preprocessing pipelines are applied to prepare imaging data for AI models?
- RQ4: What AI algorithms and development methods have been used for TBI assessment over the past decade?
- RQ5: How are these methods evaluated, what is their performance, and what limitations do they present?
- RQ6: What challenges persist, and what potential solutions could enhance AI adoption in clinical practice?

We adopted the PRISMA (Preferred Reporting Items for Systematic Reviews and Meta-Analyses) guidelines for a rigorous approach in selecting literature. However, this study constitutes a systematized review rather than a full systematic review, and certain limitations in our coverage are acknowledged. A screening process excluded studies due to availability, accessibility, and relevance to this review. While we have strived for a comprehensive and unbiased selection, the process may still reflect criteria that introduce potential biases. Furthermore, the present analysis does not evaluate or compare the performance of AI models against real-world clinical gold standards, as such validation falls beyond the scope of this review. Consequently, bias assessment frameworks such as QUADAS-2 (Quality Assessment of Diagnostic Accuracy Studies-2) and PROBAST (Prediction Model Risk of Bias Assessment Tool) are also not applied.

This paper is structured as follows: the Methods section outlines the required background knowledge and presents our methodology. The Results section presents the review results of AI applications in TBI assessments, including datasets and data processing, TBI detection, hematoma localization, MLS measurement, and ICP estimation. Finally, we discuss current limitations and propose future research directions to advance AI integration into clinical practice in the Discussion section.

The research questions are addressed as follows: RQ1 is addressed in the Search Results section, RQ2-RQ3 are addressed in the TBI Datasets and Data Preprocessing Pipeline section, RQ4 is addressed in the Binary TBI Classification through ICP Estimation sections, RQ5 is synthesized across the Results and Discussion sections, and RQ6 is discussed explicitly in the Discussion section.

### Background

TBI is a description of structural abnormalities (damage) in the brain such as hematomas, MLSs, and increased ICP. Hematomas are one of the most common complications in TBI and are broadly classified into extra-axial and intra-axial types based on their location relative to the brain tissue. Extra-axial hematomas include epidural hematomas (EDHs), subdural hematomas (SDHs), and subarachnoid hemorrhages (SAH), while intra-axial hematomas comprise intraparenchymal hemorrhages (IPHs) and intraventricular hemorrhages (IVHs) [24,25]. CT remains the primary imaging modality for detecting and characterizing these hemorrhagic lesions. Clinicians assessing CT scans with presented hematomas have a visualization of the size, location, and extent of bleeding. This information serves as vital prognostic indicators [26,27] determining the urgency and type of intervention, such as surgical evacuation or conservative management.

MLS is another critical quantitative measure in TBI assessment [26,28]. It arises when elevated ICP or mass effects from conditions such as hematomas or brain swelling force brain structures away from their normal anatomical positions. MLS is assessed in CT scans by measuring the displacement of midline anatomical landmarks, such as the septum pellucidum or falx cerebri [29]. Clinicians measure the MLS by identifying the ideal midline (iML), which is often aligned with the falx cerebri’s attachment to the skull, then calculating the distance between the iML and a specific brain structure that has shifted, such as the septum pellucidum.

ICP is another determinant of TBI severity. Elevated ICP is observed in brain injuries due to the mass effect from ICH [28]. An increase in ICP decreases brain blood flow pressure (ie, cerebral perfusion pressure), resulting in compression, deformation, and herniation of brain tissue, which can exacerbate the injury [30,31]. Clinical intervention is necessary when ICP exceeds 22 mm Hg; levels above this threshold lead to an increased risk of fatality [32]. Clinical guidelines recommend ICP monitoring for patients with a GCS score <8 and an abnormal head CT. Abnormal CT findings include mass lesions (eg, hematoma or contusion), cerebral edema or swelling, MLS, and compressed basal cisterns, particularly the perimesencephalic cisterns [33]. Currently, ICP measurements are invasive, whereby clinicians adopt ventricular or parenchymal pressure monitoring [34,35]. Despite this direct approach in measuring ICP, CT imaging allows for indirect ICP assessments. Radiological indicators for assessing ICP through CT scans include compressed ventricles, effaced sulci, and MLS, all suggestive of elevated ICP.

AI has shown promise in streamlining TBI characteristics from CT scans by adopting computer vision techniques for detecting brain structural abnormalities. This can support clinicians in triaging TBI cases, reducing the time to assessment and intervention for severe cases [23]. In addition, AI can support clinicians in reducing diagnostic errors [36]. One common AI technique is classification, where an AI model is trained to label a CT scan with a predefined category (eg, normal or abnormal), based on examples it has seen before. Another helpful technique is object detection, which identifies the presence of an abnormality such as a hematoma and provides additional information such as its location in the scan. Segmentation is another AI technique that separates the image into different regions, clearly outlining the edges of abnormal areas. This makes it easier to study the shape and structure of parts of the brain such as lesions. Building on segmentation, quantification techniques measure specific details of these abnormalities, such as the volume of a hematoma or the distance of the brain’s MLS.

## Methods

### Protocols

This study was conducted as a systematized review following the PRISMA 2020 reporting guideline [37]. While the literature search, study selection, and reporting were performed using systematic and reproducible procedures, this review does not meet all methodological criteria required for a full systematic review. PRISMA guidance was therefore used as a framework to promote transparency and completeness of reporting, rather than to imply full compliance with all requirements of a registered systematic review (Checklist 1). Specifically, no formal protocol was registered in a public database such as PROSPERO prior to conducting the review. Study screening and data extraction were performed by a single reviewer (VTNP). Furthermore, the review focuses on mapping AI tasks, methodologies, datasets, and evaluation practices rather than estimating overall diagnostic performance of those models. The present analysis does not evaluate or compare the performance of AI models against real-world clinical gold standards, as such validation falls beyond the scope of this review. Consequently, bias assessment frameworks such as QUADAS-2 and PROBAST are also not applied. To address potential biases relevant to AI research, we focused on examining dataset characteristics, annotation transparency, and validation strategy. The implications of these factors for interpretation and generalizability are discussed explicitly in the Discussion section.

### Search Strategy

The search was conducted across 4 major scientific databases including PubMed, Scopus, Web of Science, and IEEE Xplore, covering publications available up to December 31, 2025. Search queries combined keywords related to AI, machine learning (ML), deep learning (DL), CT imaging, and TBI, supplemented with MeSH terms. A detailed list of the search queries used is reported in Table S1 in Multimedia Appendix 2.

### Screening Strategy

The screening strategy for this review was designed to select studies aligned with the research objectives; refer to Table S2 in Multimedia Appendix 2 for our inclusion and exclusion criteria. This review focused exclusively on AI approaches applied to noncontrast head CT imaging. Studies incorporating multimodal inputs such as clinical scores, physiological signals, or laboratory data were excluded to maintain a focused analysis of imaging-specific methodologies and evaluation practices. This restriction was intended to limit methodological heterogeneity and to reflect the central role of CT imaging in acute TBI assessment workflows.

Eligible studies included studies that (1) were published as a peer-reviewed journal paper or conference paper, (2) used CT scans as input data, and (3) involved the development or application of AI algorithms for TBI assessment, including tasks such as hemorrhage segmentation, MLS measurement, or ICP estimation.

Studies were excluded based on (1) duplication or (2) retracted publications; (3) imaging modalities used other than CT scans (eg, MRI and electroencephalogram); (4) nonhuman studies; (5) conditions unrelated to TBI (eg, stroke, brain tumors, or other neurological disorders); (6) infants or pediatric populations, as TBI and ICP differ from adults; (7) non-English language publications; (8) nonoriginal research (eg, reviews, meta-analyses, and secondary analyses) that are limited in research details; (9) statistical analysis, therapeutic techniques, and pathological investigations not in the field of AI; or (10) nonrelevant outcomes (eg, hematoma expansion, patient prognosis, and risk prediction). These exclusion criteria ensure that the review remains concentrated on AI applications for detecting TBI-related abnormalities.

### Search Results

All records retrieved from the database searches were exported to Zotero—a reference management tool, where duplicates were identified and removed. Titles and abstracts were screened against the eligibility criteria, followed by full-text screening of potentially relevant papers. Study selection was performed by one reviewer; no automation tools were used for decision-making, and no second reviewer screening was performed, which may increase the risk of selection bias. To manage the scope of this systematized review, an exceptionally stringent filtering approach was applied during the title and abstract screening phase. Studies exhibiting any ambiguity regarding imaging modality (eg, lack of explicit noncontrast CT confirmation), patient demographics, or the application of AI methodologies were excluded at this initial stage. Consequently, the 101 papers that advanced to the full-text assessment had already strictly satisfied all inclusion criteria based on their abstracts, resulting in zero subsequent exclusions during the full-text confirmation phase.

By screening the 674 publications found, we identified 101 studies for evaluation. To address RQ1, the reviewed studies are first grouped according to the primary TBI assessment task they aim to solve. We grouped these 101 studies into 4 categories: binary TBI classification (TBI or non-TBI), hemorrhage detection (classification, localization, segmentation, and quantization), MLS measurement, and increased ICP estimation. It is evident that the literature heavily focuses on hemorrhage detection, which accounts for 85% (86/101) of the total studies. The remaining studies are distributed across MLS measurement (8/101), binary TBI classification (TBI or non-TBI; 4/101), and increased ICP estimation (3/101). The remainder of this review focuses on AI applications in TBI, identifying the techniques applied and results presented in these 101 studies.

### Data Extraction

A structured data extraction form was designed a priori and piloted on a small subset of studies to ensure consistency and clarity. For each study included, one reviewer extracted the data. When information was unclear or missing, we relied on the published paper and supplementary materials; no contact with study authors was attempted.

From each study, we extracted:

- Bibliographic details: first author, year of publication, and country or region.
- Dataset details: scanner type where reported, slice thickness, number of CT scans, source of data (public vs institutional and single- vs multicenter), and whether external datasets were used.
- Annotation and reference standard: abnormality types annotated (eg, ICH subtypes, MLS, mass effect, and features used as ICP surrogates), annotation procedures (eg, number and expertise of raters), and label granularity (scan-level vs slice- or voxel-level).
- AI methodology: model family (eg, convolutional neural networks [CNNs], transformers, and traditional ML), input representation (2D, 2.5D, 3D, and patch-based), preprocessing and augmentation steps, and training strategies (eg, transfer learning, self-supervision, and ensemble methods).
- Evaluation: internal and external validation strategy, train-validation-test split, cross-validation schemes, and any comparison with human readers.
- Outcomes and performance metrics: reported metrics such as accuracy, sensitivity, and specificity as applicable.

When an outcome or methodological detail was ambiguously reported, we recorded the information as described by the authors and noted any uncertainties qualitatively in the synthesis. To accurately assess the reproducibility and completeness of the published literature, no contact with study authors was attempted to retrieve missing data or methodological justifications not included in the peer-reviewed texts. We treated the performance metrics reported by the primary studies as the main effect measures. Given the heterogeneity of tasks, datasets, and metrics, these effect measures were summarized descriptively rather than pooled quantitatively.

### Synthesis Methods

Because of substantial heterogeneity in clinical tasks (eg, binary TBI classification, hemorrhage detection and segmentation, MLS estimation, and ICP assessment), datasets (public vs institutional, sample sizes, and annotation strategies), and reported outcome metrics, we did not attempt a meta-analysis. Instead, we conducted a narrative synthesis. Studies were grouped by primary clinical task and, where relevant, by model family. Within each group, we compared model architectures, training strategies, preprocessing approaches, and reported performance metrics. To evaluate data preparation trends, we systematically extracted all reported preprocessing techniques from the included studies (eg, windowing, skull stripping, and normalization). These discrete techniques were then qualitatively grouped into conceptual categories and ordered temporally to synthesize a consensus, 9-stage sequential preprocessing pipeline, providing a conceptual framework that maps current developmental practices.

We did not formally evaluate publication bias or small-study effects, as the included studies were highly diverse and rarely reported sufficient detail for such assessments. Similarly, we did not calculate graded certainty of evidence (eg, using GRADE [Grading of Recommendations Assessment, Development and Evaluation]) because the primary aim was to describe the methodological landscape rather than to derive pooled effect estimates for a specific clinical outcome.

We did not conduct a formal risk-of-bias assessment using tools such as QUADAS-2 or PROBAST because our primary focus was to map and characterize AI methodologies, tasks, and evaluation practices rather than to synthesize pooled estimates of diagnostic accuracy or prognostic performance. Instead, we qualitatively appraised methodological aspects likely to affect bias and generalizability, including dataset size and diversity, transparency of inclusion and exclusion criteria, clarity of annotation procedures, internal versus external validation, and reporting of model development and evaluation.

## Results

### Overview

**Figure 2.**
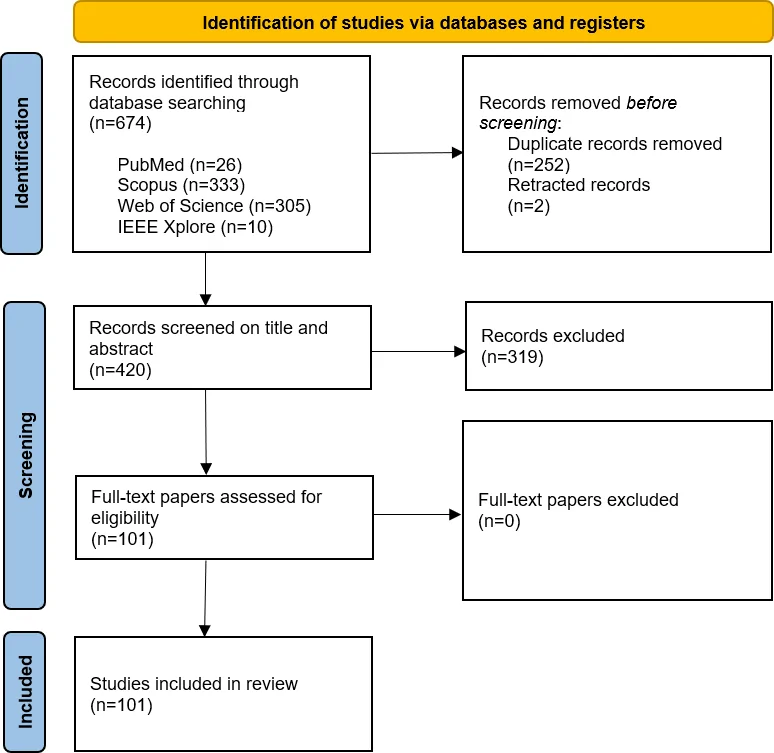
PRISMA (Preferred Reporting Items for Systematic Reviews and Meta-Analyses) 2020 flow diagram detailing the literature search, screening, and selection process for the systematized review of AI applications in computed tomography–based traumatic brain injury assessment, resulting in the inclusion of 101 peer-reviewed studies published up to December 2025.

By screening the 674 publications found, we identified 101 studies for evaluation. The overall selection process and study count at each stage are summarized in the PRISMA flow diagram (Figure 2).

### TBI Datasets and Data Preprocessing Pipeline

#### Data Preprocessing Pipeline

Preparing CT scan data for training AI models is crucial for accurate model performance in TBI assessments. The data preprocessing stage is important to reduce noise in the dataset to enhance anatomical clarity and ensure that the data are formatted and scaled appropriately for the selected AI architecture. Based on the 101 reviewed studies, we propose a sequential preprocessing pipeline composed of 9 key stages that have been identified (Textbox 1).

**Figure 3.**
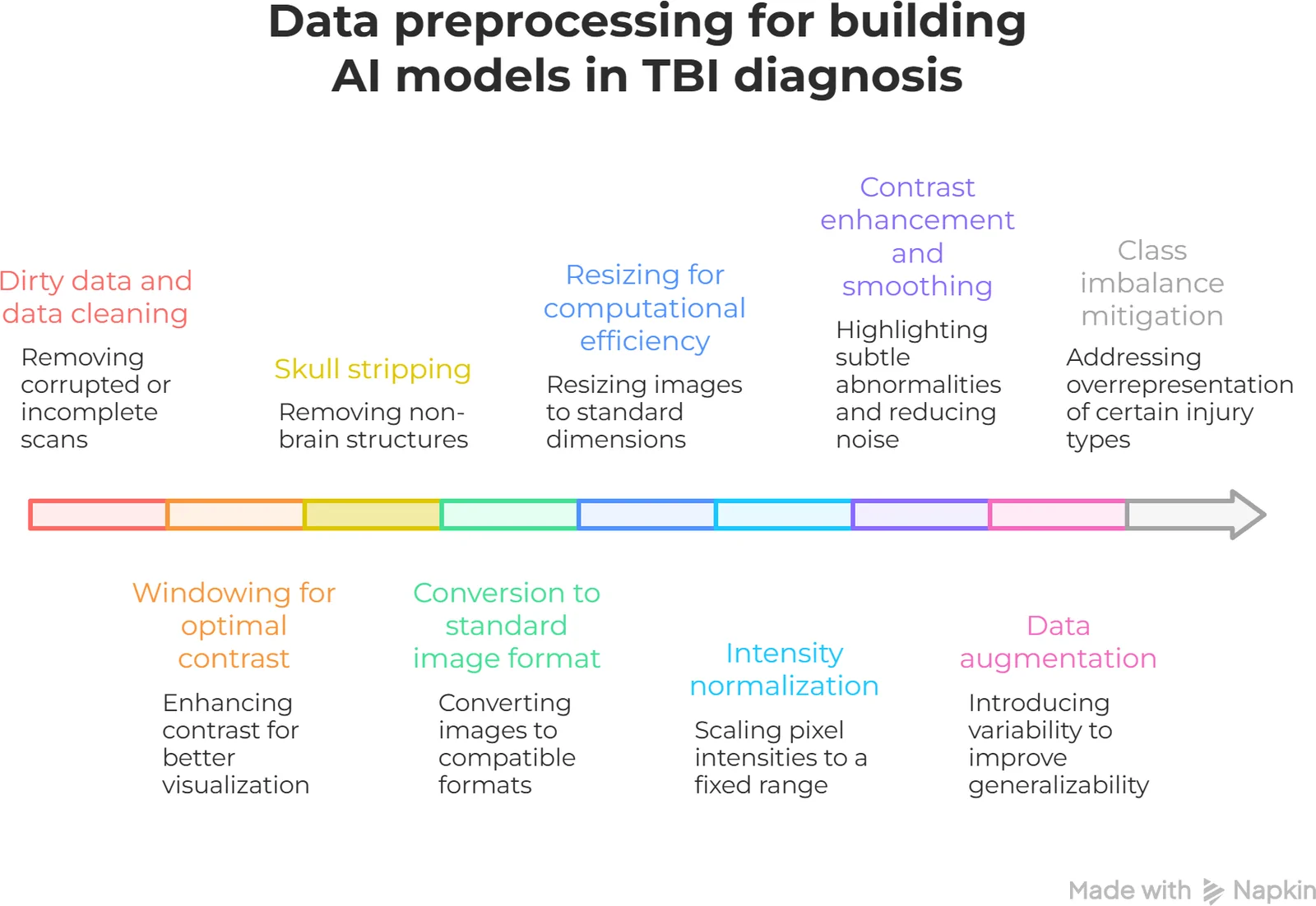
Proposed sequential 9-stage data preprocessing pipeline for preparing raw, unenhanced head computed tomography scans for AI model training and evaluation in TBI applications. TBI: traumatic brain injury.

Figure 3 presents this proposed 9-stage preprocessing pipeline.

Textbox 1. **Proposed 9-stage preprocessing pipeline.**

Dirty data and data cleaning: Dirty data refer to data with excessive motion artifacts, missing slices, or misaligned metadata. This can introduce noise or mislead the learning process, resulting in model bias or decreased predictive accuracy. Therefore, the first stage of the data preprocessing pipeline is to conduct a data cleaning process to remove corrupted, incomplete, or duplicate scans.

Windowing for optimal contrast: This stage involves applying windowing techniques to enhance the contrast of computed tomography (CT) scans. This allows brain structures of interest to be distinguishable compared to other structures. This is significant in traumatic brain injury (TBI) for differentiating between gray matter, white matter, cerebrospinal fluid, hemorrhages, and the skull. CT scans use a Hounsfield unit (HU) scale to represent tissue density. Standard window settings used in TBI assessment include:

- Brain window: width=80 HU, level=40 HU
- Subdural window: width=150 HU, level=50 HU
- Soft tissue window: width=375 HU, level=40 HU

Skull stripping: For the third stage, nonbrain structures (eg, the skull) are removed to prevent interference during lesion segmentation. This is typically performed using intensity thresholding since bone appears hyperdense (>1000 HU) compared to brain tissue (20-80 HU). In TBI assessment, skull stripping allows models to focus on low- to mid-intensity thresholds where lesions (eg, hemorrhages or edema) are most likely to occur, thereby improving detection accuracy and reducing computational complexity.

Conversion to standard image format: Images are converted from their native Digital Imaging and Communications in Medicine format to standardized formats such as PNG or JPG. This step ensures compatibility with most deep learning frameworks, which are optimized for standard image inputs. Although this conversion can introduce minor compression artifacts, it simplifies the data pipeline integration and reduces storage overhead. Moreover, it enables batch loading and further preprocessing with existing computer vision libraries.

Resizing for computational efficiency: This stage involves resizing the CT scans to standard dimensions, such as 512×512, 256×256, or 128×128, and the selection of dimensions is dependent on the model architecture and graphics processing unit memory constraints. Uniform image size across the dataset is essential to ensure consistent input dimensions, which prevents shape mismatches and simplifies batch processing. For TBI-focused models, resizing must preserve lesion boundaries and anatomical landmarks like ventricles or cisterns.

Intensity normalization: After resizing, pixel intensities are normalized using min-max scaling, mapping values to a fixed range of [0,1]. For example, if a CT scan has pixel intensity values ranging from 30 to 200, min-max normalization would transform the value 30 to 0 and 200 to 1, with all other values scaled proportionally within this range. A pixel value of 115, which is halfway between 30 and 200, would be normalized to approximately 0.5. Normalization for deep learning models improves accuracy by ensuring that features are a similar scale. Without normalization, features with higher pixel intensity might dominate the model's training due to their larger values. This stage is important for gradient-based optimization architectures such as Adam or Stochastic Gradient Descent.

Contrast enhancement and smoothing: Contrast enhancement techniques, such as histogram equalization or adaptive contrast enhancement, are used to highlight subtle abnormalities including small contusions or early-stage hemorrhages. Gaussian smoothing is then applied to reduce high-frequency noise, such as random bright spots or jagged edges, without significantly blurring important anatomical boundaries to emphasize lesion borders while minimizing false positives caused by noise.

Data augmentation: Data augmentation techniques are applied to introduce variability within the dataset to improve model generalizability and mitigate overfitting. Common augmentation techniques include random rotations, flips, and brightness adjustments. Some studies also leverage generative adversarial networks (GANs), which are generative models consisting of a generator and discriminator trained adversarially to synthesize realistic data samples, such as deep convolutional GAN [38] and CycleGAN [39], to synthesize new training examples, especially for underrepresented lesion types. These techniques are particularly valuable in TBI datasets, where some TBI conditions are less frequently observed.

Class imbalance mitigation: Class imbalance is a common challenge in TBI datasets where certain injury types or severities are overrepresented. This is addressed through resampling techniques such as the synthetic minority oversampling technique. Generating synthetic examples of underrepresented injury severities or features results in a balanced dataset. This is to reduce bias and improve model performance in classifying different TBI conditions.

#### Annotation Methods

A critical gap identified across the literature concerns the lack of transparency regarding annotation methodologies and data labeling practices. Whether examining studies using publicly available datasets or in-house datasets, the reporting typically focuses exclusively on dataset characteristics such as sample size, label categories, label distributions, and basic imaging parameters (eg, image size). There is no information about annotation protocols or the specific tools used.

#### Publicly Available TBI Datasets

Currently, publicly accessible TBI-related CT datasets are available to support model development. The 3 common datasets used in the literature are CQ500 [21], Radiological Society of North America (RSNA) [40], and PhysioNet [41]. Table 1 provides a detailed comparison of the dataset in terms of number of scans, CT slice thickness, annotation granularity, data sources, and the used CT scanner models.

**Table 1.** Characteristics of commonly used publicly available head computed tomography (CT) datasets (RSNA, CQ500, and PhysioNet) for training and evaluating AI models in traumatic brain injury (TBI) assessment, including sample sizes, annotation granularity, and institutional data sources.

	RSNA [40]	CQ500 [21]	PhysioNet [41]
CT scans or patients, n	25,272 scans	491 scans	82 patients
CT slices, n	870,301	193,317	2814
Slice width (mm)	3‐5	5	5
Annotated TBI	Intraparenchymal Intraventricular Subarachnoid Subdural Epidural	Intraparenchymal Intraventricular Subarachnoid Subdural Epidural Midline shift Mass effect Fracture	Intraparenchymal Intraventricular Subarachnoid Subdural Epidural
Annotation level	Slice	Scan	Pixel
Annotators, n	60	3	2
Data source	Federal University of São Paulo (2018) Stanford University (1999‐2014) Thomas Jefferson University Hospital (N/A^a^)	Centre for Advanced Research in Imaging, Neurosciences and Genomics (2017)	Al Hilla Teaching Hospital, Iraq (2018)
CT scanner models	N/A	GE BrightSpeed GE LightSpeed GE Discovery CT750 HD GE Optima CT660 Philips MX16 Philips Access CT 32	Siemens SOMATOM Definition AS

a N/A: not available.

The RSNA dataset contains over 25,000 head CT scans sourced from multiple institutions. Despite this dataset not being exclusive to TBI, it includes annotations for various types of ICHs relevant to TBI assessment, including epidural, subdural, and subarachnoid. Similarly, the CQ500 dataset includes 491 head CT scans from suspected patients with brain trauma or stroke. Each scan is annotated at the scan level for clinically significant findings, including MLS, mass effect, skull fractures, and hemorrhagic types. The PhysioNet ICH dataset is specific to TBI cases containing pixel-level annotations of 5 hemorrhage types across 82 patients. While small in size, it is unique in offering segmentation masks. In this dataset, scans were acquired using the same Siemens scanner model, limiting variability due to acquisition hardware.

Restricted large data repositories include the Federal Interagency Traumatic Brain Injury Research Informatics System (FITBIR) [42], Medical Information Mart for Intensive Care III (MIMIC-III) [43], and eICU [44]. FITBIR is a collaborative initiative created by the National Institutes of Health and the Department of Defense, providing a centralized repository for raw data from numerous studies, including clinical evaluations, medical imaging, and behavioral assessments. FITBIR includes the Transforming Research and Clinical Knowledge in TBI (TRACK-TBI) dataset [45] established by the National Institute of Neurological Disorders and Stroke. It contains standardized clinical data from 3000 participants across 18 sites in the United States, including blood biospecimens, CT or MRI scans, and detailed clinical outcomes. The use of standardized data ensures consistency in variables such as imaging protocols, clinical outcome assessments, and patient metadata, thereby facilitating cross-study comparison and reliable model validation. Despite the TBI data found in the MIMIC-III [43] and eICU [44] datasets, they are generic intensive care unit datasets that contain electronic health record (EHR) data. These datasets provide physiological data that can be paired with scan data from other sources for multimodal modeling. However, manual extraction or filtering for TBI-related records is necessary to remove data for nontraumatic brain conditions such as tumors or strokes.

### Binary TBI Classification

Clinicians assessing TBI examine CT scans for TBI abnormalities. This has been translated into AI binary classification tasks that identify the presence (or absence) of TBI. Dusenberry et al [46] used an artificial neural network (ANN) model to classify CT scans by detecting acute CT findings indicative of TBI. Their model achieved an accuracy of 93.14%, a recall of 97.78%, and a negative predictive value of 98.08%. These results demonstrate the model’s effectiveness in minimizing missed diagnoses. However, the model’s precision of 88% suggests a high rate of false positives. Gan et al [47] proposed a more complex architecture that combined a ResNet backbone with a squeeze-and-excitation (SE) module and a long short-term memory (LSTM) network. The model was initialized with ImageNet-pretrained weights and fine-tuned for the TBI classification task. It achieved an accuracy of 95.9%, with a sensitivity of 93.3% and a specificity of 98.9%. Zhang et al [48] extended this approach by integrating a traditional VGG-S CNN with both an SE module and a pixel-wise correlation retaining (PCR) module. This architecture, termed VGG-SE-PCR, improved feature extraction and retention of spatial relationships in CT scans. Although the model achieved a slightly lower overall accuracy of 89.3%, it exhibited a high sensitivity of 98.6%, suggesting a strong capability to detect TBI. However, its relatively low specificity of 82.9% demonstrates vulnerability to false positives. Ellethy et al [49] introduced a multimodal framework that combined CT imaging with clinical data using a region-based CNN architecture. The model achieved a comparatively lower performance, with accuracy, sensitivity, and specificity values of 82.4%, 82%, and 82%, respectively. These results suggest that while clinical data can offer valuable context, their inclusion may also introduce noise or increase model complexity if not effectively integrated. Despite these findings, Ellethy et al [49] highlighted the importance of a holistic approach to TBI assessment, suggesting that combining multiple modalities could be key to capturing the multifaceted nature of TBI presentations.

Across binary TBI classification studies, performance differences are driven primarily by model complexity and input aggregation strategy rather than dataset size alone. First, models relying exclusively on CT imaging consistently outperform naive multimodal approaches that incorporate clinical variables without structured fusion mechanisms. This suggests that poorly aligned clinical features may introduce noise rather than complementary information. Second, early ANN-based approaches achieve high sensitivity and negative predictive value, reflecting suitability for rule-out triage in older cohorts, but rely on handcrafted features and lack spatial modeling capacity. Meanwhile, CNN-based architectures augmented with channel attention (SE blocks) and temporal modeling (LSTM) demonstrate the highest overall accuracy and specificity, indicating improved discrimination of subtle injury patterns across CT slices. However, most binary classification models are evaluated on internal datasets only, limiting conclusions about generalizability. Moreover, despite improvements in accuracy and sensitivity, the challenge of balancing specificity to reduce false positives remains an open research question. Table S1 in Multimedia Appendix 3 [46-49] summarizes the reviews in TBI binary classification.

### Hemorrhage Detection, Classification, Segmentation, Localization, and Quantification

#### Overview

In total, 85% of our literature review focuses on leveraging AI for detecting and classifying hematomas in CT scans. In this section, we categorize the hematoma literature into 4 primary subgroups: binary hemorrhage detection, hemorrhage subtype classification, hemorrhage localization and classification, and hemorrhage segmentation and volume quantification*.* Binary hemorrhage detection identifies the presence or absence of bleeding in a scan. Hemorrhage subtype classification differentiates the types of ICH, such as EDH, SDH, SAH, and IPHs. Hemorrhage localization and classification techniques identify the location of hematomas and classify them accordingly. Hemorrhage segmentation and volume quantification apply linearization to measure the size of the hematoma.

#### Binary Hemorrhage Detection

The detection of ICH in TBI cases is fundamental for early diagnosis by determining the presence of bleeding in CT scans. From the 85% literature in hemorrhage detection, 9 reviewed studies were binary detection, and 89% (8 studies) of those studies used CNNs—a DL architecture that uses convolutional filters to capture spatial patterns, while only 1 study used a vision transformer (ViT)—a transformer-based architecture designed for image analysis [50] (Table S2 in Multimedia Appendix 3 [39,50-57]).

Sindhura et al [51] proposed a 2-stage DL framework incorporating an Intensity Transformed Sinogram Synthesizer and a cascaded CNN-RNN (recurrent neural network) model. This hybrid model captures high-level feature representations and spatial correlations within sinograms with an accuracy of 95.50%. However, the sinogram synthesis adds complexity that can be computationally costly and is not necessary. Altuve and Pérez [52] used transfer learning with a pretrained ResNet-18 model and achieved a similar accuracy of 95.93%. Similarly, Ganeshkumar et al [39] developed a ResNet50-based model; however, due to a small dataset of 82 patients with 2814 CT slices, and a class imbalance (only 318 CT slices with ICH, which is 11.3%), they used CycleGAN to synthesize hemorrhagic CT slices. Despite an increase in accuracy (96%) without compromising specificity (99%), the use of synthetic data raises concerns about ethical implications and the validity of training on artificially generated images. Agrawal et al [53] also adopted a CNN-based approach, implementing a 3D CNN to process volumetric data such as CT scans. However, in comparison, their results were moderate, achieving 80% accuracy, 90% sensitivity, and 70% specificity.

Improving upon these CNNs, Kar et al [54] introduced a CNN with integrated attention mechanisms to focus on key areas indicating ICH in the CT scans. This model achieved a near-perfect performance with an accuracy of 99.76%. However, this raises concerns on potential overfitting, whereby noisy and heterogeneous real-world medical data can result in lower accuracies. Attention mechanisms were also used by Pérez-Cano et al [55], who developed 2 multiple instance learning models, E2E-Att-GP and E2E-GP-Att, integrating CNNs, attention, and sparse Gaussian processes (SGPs). In the E2E-Att-GP architecture, features extracted from CT slices are weighted via an attention mechanism before classification by an SGP. In contrast, the E2E-GP-Att model first feeds the feature vectors directly into an SGP layer, followed by an attention mechanism. The E2E-Att-GP model achieved an accuracy of 87.6% and an area under the receiver operating characteristic curve (AUC-ROC) of 0.965 on the RSNA dataset. However, this performance dropped when testing it on the CQ500 dataset, resulting in an accuracy of 79.6% and an AUC-ROC of 0.918. López-Pérez et al [56] attempted to improve feature modeling by introducing the deep Gaussian processes for multiple instance learning model, which combines an attention-based CNN with Gaussian processes. This 2-phase training process initially applies a CNN with an attention mechanism for feature extraction. Then, by fixing its weights, a multilayer Gaussian process model is trained on those features using doubly stochastic variational inference. The deep Gaussian processes for multiple instance learning achieved 82.5% accuracy and an AUC-ROC of 0.957 on RSNA data. However, this too showed a reduction in performance on CQ500 with an accuracy of 71.7% and an AUC-ROC of 0.909. The discrepancy across various datasets in these 2 studies implies that these models may be overly tuned to the RSNA dataset, limiting generalization to diverse datasets in real-world applications.

Recently, researchers have applied other techniques including a transformer architecture and LSTM. Fan et al [50] introduced the transformer-based architecture, dual-task ViT. Dual-task ViT used the encoder of the ViT-large model pretrained on ImageNet-1K. The model achieved near-perfect performance with an accuracy of 99.68%, a precision of 99.35%, a recall of 99.78%, and an *F*_1_-score of 0.9957. However, as with Kar et al [54], such near-perfect metrics raise concerns about potential overfitting. In addition, ViT models require substantial computational resources and large datasets for effective training; thus, this limits the clinical feasibility of deploying such models, particularly in resource-limited settings. Wu et al [57] developed a weakly supervised DL framework combining transfer learning with an attention-based bidirectional long short-term memory (Bi-LSTM). The model was trained in 3 stages: first, an EfficientNet-B2 network was trained on scan-level labels from the RSNA dataset; second, a Bi-LSTM with attention was trained to model interslice dependencies; finally, the entire pipeline was fine-tuned using data from a local institution (10,699 scans from 7469 patients). This approach achieved an AUC-ROC of 0.96 on both internal and external datasets. However, the reliance on multistage training may hinder its adaptability for clinical sites with limited labeled data.

Binary hemorrhage detection performance varies substantially with evaluation dataset and supervision strategy rather than architectural choice alone. Models evaluated solely on large internal or private datasets, including attention-augmented CNNs and transformer-based architectures, frequently report near-perfect metrics, raising concerns about overfitting and limited external validity. In contrast, methods explicitly evaluated on both RSNA and CQ500 consistently demonstrate a measurable performance drop on CQ500, as observed in multiple instance learning frameworks and probabilistic Gaussian process–based models, highlighting domain shift effects across institutions and annotation protocols. Overall, binary hemorrhage detection appears to be a mature task on internal datasets, but performance on public benchmarks indicates that generalization remains the primary unresolved challenge.

#### Hemorrhage Subtypes Classification

For hemorrhage subtype classification, researchers have used ensemble techniques, which use multiple models with individual weighted coefficients. Malik and Vidyarthi [58] proposed an ensemble model combining SE-ResNeXt-50, EfficientNet-B3, and EfficientNet-B2. Each model generated a prediction vector, with an assigned weight. These weighted vectors were averaged to obtain the final prediction, achieving an accuracy of 98.56%. Similarly, Gudadhe and Thakare [59] introduced the weighted average 2D-CNN model, an ensemble of 3 distinct 2D-CNN architectures with 12, 11, and 9 layers. Grid search was used to assign optimal weights to each model, resulting in an accuracy of 95.86%.

Hybrid approaches decoupling feature extraction and classification have also been explored. Gençtürk et al [60] proposed a 2-stage segmentation-classification pipeline. The first stage used an MS R-CNN model with a ResNet-101+FPN (residual network+feature pyramid network) backbone to segment hemorrhage regions, and the second stage used EfficientNet-B2 to classify these regions. This method achieved accuracies of 94.3% on the CQ500 dataset and 97.3% on a private dataset. Umapathy et al [61] combined SE-ResNeXt for feature extraction with LSTM networks to capture sequential dependencies, resulting in an accuracy of 94%. Asif et al [62] used a combination of ResNet-101-V2 and Inception-V4 for feature extraction, followed by light gradient boosting machine for classification. Their model resulted in accuracies above 97% across the hemorrhage subgroups, specifically SDHs 98.7%, SAH 98.5%, EDHs 97.5%, IPHs 98.7%, and IVHs 98.5%. Naeem Akram et al [63] introduced a double-branch model based on the Xception architecture. One branch processed 3D images generated by concatenating grayscale images across different intensity windows. These intensity windows comprising the brain window (*L*=40; *W*=80), subdural window (*L*=100; *W*=200), and bone window (*L*=600; *W*=2800). The other analyzed neighboring slices with the skull removed to capture 3D spatial context. Features from both branches were concatenated and fed into a decision tree classifier, achieving an accuracy of 97.35%. Similarly, Ozaltin et al [64] developed a CNN named OzNet for feature extraction, followed by neighborhood component analysis (NCA) to select meaningful features. Classification was performed using various ML algorithms: ANN, AdaBoost, Bagging, decision tree, k-nearest neighbor, linear discriminant analysis, naïve Bayes, and support vector machine. Among them, the OzNet-NCA-ANN combination performed best, reaching an accuracy of 99.58%. The other configurations scored as follows: OzNet-AdaBoost 99.37%, OzNet-Bagging 99.58%, OzNet-decision tree 96.44%, OzNet-k-nearest neighbor 98.74%, OzNet-linear discriminant analysis 98.95%, OzNet-naïve Bayes 98.75%, and OzNet-support vector machine 99.37%. In another work, Sengupta et al [65] applied classical image processing for feature extraction. In this framework, Otsu’s thresholding technique was first used to segment the region of interest (RoI) by analyzing the grayscale intensity (Hounsfield unit [HU] values) distribution of the CT images. This method determines the maximum separability of the different ICH classes. Hybrid feature extraction was then performed using Tamura features (directionality, contrast, and coarseness) and gradient local ternary pattern descriptors to extract discriminative vectors from the segmented RoI regions. Tamura features capture texture characteristics, while gradient local ternary pattern encodes local texture information. A modified genetic algorithm was also proposed for feature optimization and dimensionality reduction. This modification incorporates an infinite feature selection technique to reduce redundancy within the extracted vectors by assessing the relevance between output vectors and regularized vectors using conditional entropy. The selected optimal vectors were fed into a Bi-LSTM network for classification. Despite an accuracy of 80%, the model achieved a high *F*_1_-score of 0.993.

Negm et al [66] combined hybrid and ensemble techniques with optimization algorithms proposing the Willow Catkin Optimization (WCO) with Voting Ensemble (Intracranial Haemorrhage Diagnosis using Willow Catkin Optimization with Voting Ensemble) model, which involved three main stages: (1) feature extraction using multihead attention-based CNN model—MAFNet, (2) hyperparameter tuning with the WCO algorithm, and (3) ensemble learning-based classification with the Majority Voting Ensemble Deep Learning (MVEDL) model. In (2), the hyperparameters of the MAFNet model are optimally tuned using the WCO algorithm, a novel meta-heuristic optimizer. WCO uses hybrid exploration and exploitation strategies. The fitness function for the WCO algorithm is designed to minimize the classification error rate. In (3), the MVEDL model is an ensemble of 3 DL models: RNN, Bi-LSTM, and extreme learning machine-stacked autoencoder. The MVEDL uses a weighted majority voting system based on the confidence probability of each constituent model to make the final classification. The final model achieved an accuracy of 98.45%. In addition to these studies, Table S3 in Multimedia Appendix 3 [38,58-76] includes additional related work.

Across subtype classification studies, the strongest results are typically reported by CNN-based ensembles and hybrid pipelines. Methods that explicitly model interslice context (convolutional long short-term memory or CNN-RNN) generally report improved recall for multilabel subtype prediction, but performance varies by subtype, with rarer classes (notably EDH) often showing weaker sensitivity even when overall accuracy appears high. Approaches evaluated on both RSNA and CQ500 provide more credible evidence of generalization, while near-ceiling metrics on single private datasets should be interpreted cautiously due to potential dataset homogeneity and label leakage. Transformer-only subtype classification (eg, ViT) underperforms CNN-dominant approaches in this set, consistent with the practical constraint that transformer models are less sample-efficient when domain-specific pretraining and large curated datasets are unavailable. Overall, subtype classification remains limited less by architecture and more by class imbalance, heterogeneous labeling standards, and cross-dataset robustness, which are only partially addressed by ensembles and sequential modeling.

#### Hemorrhage Localization and Classification

Recently, researchers have focused on the classification and localization of hemorrhages in CT scans, leveraging transfer learning, attention mechanisms, and optimization strategies to improve the accuracy and efficiency of these models. Kothala and Guntur [77] introduced the transfer learning–based lighter, faster, and frozen network, built on a single-stage object detection framework similar to YOLO. The model used CSPDarknet53 as the feature extraction backbone, pretrained on ImageNet, and incorporated transfer learning to reduce training time while improving performance. Model hyperparameters were optimized using a genetic algorithm, which mutated the top-performing sets from each generation to create new ones. This transfer learning–based lighter, faster, and frozen model achieved an accuracy of 98.7% and specificity of 99.3%. He et al [78] proposed a multiscale feature classification and weakly supervised localization framework, which incorporated three key components: (1) a window tuning optimization module, (2) a multiscale feature fusion module, and (3) a bleeding lesion localization module. The window tuning optimization module was designed to enhance the visibility of ICH in CT scans by mimicking the way radiologists adjust window width and level. It replaced the initial layer of the VGG-16 backbone network and used a 1×1×3 convolution layer with a custom activation function that has learnable parameters. This enabled dynamic adjustment of window settings during training to better capture relevant features from the original Digital Imaging and Communications in Medicine image information. The multiscale feature fusion module integrated features from different convolutional layers (C3, C4, and C5) of the VGG-16 network. It used channel and spatial attention mechanisms to aggregate these multiscale features, enabling the model to focus on critical areas. The final module, bleeding lesion localization module, used class-activated mapping for weakly supervised lesion localization. The model reported an AUC-ROC of 0.973. Vidhya et al [79] proposed YOLOv5s-CAM, an improved version of the YOLOv5s model incorporating 2 major enhancements. First, a cascaded attention module (CAM) was added to the neck of the network to emphasize clinically important regions by assigning them higher attention weights. Second, they replaced the standard spatial pyramid pooling module with the more efficient spatial pyramid pooling—fast (SPPF). Unlike spatial pyramid pooling, which performs parallel max-pooling at multiple kernel sizes and requires resizing feature maps, SPPF performs a sequence of three 8×8 max-pooling operations, reducing computational load while preserving spatial information. The improved model achieved a precision of 93.5%, recall of 90.8%, and *F*_1_-score of 0.921. Kothala et al [80] proposed the YOLOv5x-GCB, an enhanced version of the baseline YOLOv5x model that reduces the model’s complexity while maintaining a high accuracy. Traditional convolution layers produce redundant feature maps, leading to inefficiencies. However, to address this, Kothala et al [80] incorporated ghost convolution that extracts intrinsic feature maps using a 1×1 convolution and then generates additional feature maps through inexpensive 5×5 linear operations. They also replaced the original cross-stage partial modules with ghost bottleneck modules to obtain quality features from linear operations. The model achieved a precision of 92.1% and an *F*_1_-score of 0.900. Cheng et al [81] modified the RetinaNet model, adding attentional gates to increase the focus on critical areas in CT scans by finding the best anchor setting to maximize the overlap between the lesion bounding boxes and the anchors. The default anchor sizes and aspect ratios in RetinaNet were not suitable for the characteristics of ICH lesions; therefore, they included a differential evolution search algorithm to optimize the ratio and proportion of anchor points on the validation set. In addition, they improved the loss function to handle class imbalance, ensuring that the model learns from both common and rare cases. Their approach reached an overall AUC-ROC of 0.956, with subtype-specific area under the curve of 0.965 for IVHs, 0.972 for SDHs, and 0.948 for SAH. However, this study omits detailed information regarding the size, the specific characteristics, and the diversity of the training dataset, whereby the latter is a common challenge among the presented studies. Further research is needed to improve generalization across different patient populations.

Across localization-focused studies, single-stage object detectors (YOLO-family) dominate due to their favorable speed-accuracy trade-off and their native support for bounding-box outputs, with recent variants improving performance primarily through attention modules (eg, CAM or ECA or CBAM) and multiscale feature aggregation (SPPF and PANet). Weakly supervised localization approaches using CAM-based heat maps provide only coarse lesion localization and are less directly comparable to detector-based mAP, but they remain attractive when bounding-box labels are unavailable. Where metrics are reported, performance is generally stronger on BHX-style datasets with predefined bounding boxes than on settings with unclear dataset characterization, and missing dataset details substantially limit interpretability of reported AP or mAP. Overall, gains in this category are less about inventing new detectors and more about handling multiscale lesions, class imbalance, and computational constraints through targeted architectural refinements. Table S4 in Multimedia Appendix 3 [77-81] provides a summary of these studies.

#### Hemorrhage Segmentation and Quantification

Recent studies have explored different approaches to enhance hemorrhage segmentation and quantification for volume estimation through the use of various AI techniques such as multiscale feature extraction, attention mechanisms, semisupervised learning, and hybrid models combining object detection with segmentation.

Several studies have focused on improving feature extraction to boost segmentation performance. Zhang et al [82] proposed MOEL-Net, which combines shallow and deep feature extraction modules to retain spatial information while accurately capturing hemorrhage regions. By fusing multilevel semantic features, MOEL-Net achieved high segmentation accuracy with Dice scores of 0.9092 and 0.9095 on 2 datasets. However, this model is prone to segmentation errors due to misclassifying small gaps when 2 ICH regions are intricately connected. Xiao et al [83] introduced MPFR-Net, a multiscale feature perception model that distinguishes hemorrhage types by integrating global and local feature extraction. The model includes a multiscale perception module and a feature refinement module (FRM). The multiscale perception module concatenates deep features from the encoder and applies global and local branches to extract multiscale features. The feature refinement module then fuses these with shallow encoder features to enhance detail and detect small targets. The model achieved a Dice score of 0.907 for ICH and 0.879 for IPH. In another work, Xiao et al [84] developed DFMA-ICH, a 3-path model that uses mixed attention and deformable convolutions to improve feature extraction and supervision. Their model achieved Dice scores of 0.8603 and 0.8098 on their 2 datasets. However, it was trained on small private datasets of 307 CT scans. The authors acknowledged that their focus is primarily on segmentation, not encompassing the full clinical application procedure. Specifically, they highlighted the need for estimation and analysis of hemorrhage volume within a short time frame for effective preoperative preparation and improved patient prognosis. Xu et al [85] proposed a 2-stage encoder-decoder CNN. The first stage segments coarse hemorrhage areas, and the second refines this to detect finer regions. With the segmentation result, the volume of hemorrhage is further estimated by counting the segmented voxels and multiplying by voxel dimensions. This study achieved a Dice score of 0.8454. Nafees Ahmed and Prakasam [86] incorporated convolution-squeeze excitation-residual modules into a U-Net-based architecture to improve feature extraction. This model achieved Dice scores of 0.969 and 0.983 for SDH and EDH, respectively, with 100% accuracy, recall, and precision, raising concerns about potential overfitting. Liu et al [87] proposed DDANet, an enhanced U-Net framework consisting of feature extraction and feature aggregation. For feature extraction, the model used a pretrained ResNet-34 as the encoder. A novel dilated convolution pooling block is introduced in the intermediate layers of the encoder (excluding the first level) to enhance the receptive field and capture multiscale contextual information of ICHs of varying sizes. Additionally, a self-attention module was incorporated to capture global semantic information of high-level features and guide the processing of low-level features, improving the model’s ability to capture the overall structure while preserving details. For feature aggregation in the decoding phase, DDANet integrated residual networks, channel attention, and spatial attention mechanisms for joint optimization. This model achieved a Dice score of 0.712. Li et al [88] developed REUnet, a 3D CNN based on the nnU-Net framework with residual connections in its encoding block, for segmenting ICH and IVH. Despite achieving a Dice score of 0.777, its lower sensitivity of 0.707 indicates challenges in detecting smaller hemorrhage regions. Zhang et al [89] address the limitations of the fuzzy C-means algorithm that depends on data that forms round- or oval-shaped clusters when applying Euclidean distance. This fails when the data have unusual or complex shapes, since Euclidean distance may not reflect the real connections between data points [90]. Thus, Zhang et al [89] proposed a new kernel function that mapped pixel distances from the 2D space to a 3D space, transforming complex nonlinear problems into linear problems. This approach results in improved segmentation performance, achieving a Dice score of 0.91 and a root mean square error of 0.097.

Rajapakse et al [91] identified that datasets with segmentation masks are typically very small in comparison with datasets with bounding boxes. They proposed an approach using both bounding boxes and segmentation masks by combining YOLOv5 object detection with TransDeepLab segmentation to improve segmentation performance. However, this resulted in a relatively low Dice score of 0.6, suggesting that bounding box-based localization may not be as effective for precise segmentation.

To improve the generalization of segmentation models, Lin and Yuh [92] applied noisy student training, a semisupervised learning technique demonstrating that incorporating large unlabeled datasets with pseudolabeling can enhance segmentation accuracy. Their processes included (1) training a PatchFCN model on the small Atlantis pixel-labeled dataset to get a “teacher” model; (2) using the trained teacher model to generate pixel-level and scan-level pseudo labels on the unlabeled Kaggle-25K dataset; (3) ranking the unlabeled scans based on the teacher model’s predicted probability of hemorrhage, and only the top 10% of scans with the highest probability were considered positive; and (4) initializing another PatchFCN model from the teacher model’s weights and training it on the combination of the Atlantis and the Kaggle-25K dataset to get the final “student” model. This model was tested on the external CQ500 dataset. Compared to the baseline supervised model, PatchFCN that was trained on the Atlantis pixel-labeled dataset, the proposed model achieved a statistically significant higher examination of AUC-ROC (0.939, 95% CI 0.938-0.940 compared to 0.907, 95% CI 0.906-0.908; *P*=.009), Dice score (0.829, 95% CI 0.825-0.833 compared to 0.809, 95% CI 0.803-0.812; *P*=.01), and AP (0.848, 95% CI 0.843-0.853 compared to 0.828, 95% CI 0.817-0.828).

Leveraging a pretrained approach, Zhang et al [93] used GPT-4 with predefined question-answer format to directly generate annotations for ICH segments on CT scans. However, its misidentification percentages were quite high, with 50.2% for EDH, 50.5% for SAH, 54% for complex cases, 40.3% for chronic SDH, 32.6% for acute SDH, and 26.2% for IPH. The identification completeness percentage for chronic SDH was only 37.3%. Moreover, the model is sensitive to prompts, whereby inappropriate prompts can impact recognition effectiveness. Ethical and legal concerns also surround the use of AI, including GPT-4, in medical image analysis, and such technology may not be universally accepted [94,95].

Segmentation studies are dominated by U-Net-style encoder-decoder architectures and their derivatives, reflecting their strong bias toward dense prediction with limited pixel-level annotations, while reported gains typically come from multiscale context modeling (dilated convolutions and pyramid pooling) and attention mechanisms to recover small lesions and blurred boundaries. Results on small public datasets show moderate-to-strong Dice performance but also expose subtype-dependent failures, with small or thin hemorrhages (eg, EDH or SAH) consistently harder to segment than larger intraparenchymal bleeds. Methods incorporating transformer or deformable attention claim improved global context handling; yet, many are evaluated primarily on private datasets, limiting conclusions about robustness; in contrast, semisupervised learning with external testing provides more credible evidence that leveraging unlabeled data can improve generalization. Overall, recent research indicates the need for improving sensitivity, particularly for detecting small hemorrhages and reducing misclassification across different hemorrhage subtypes. Table S5 in Multimedia Appendix 3 [82-89,91-93,96-102] provides a summary of these studies and other relevant studies that achieved lower accuracies compared to those presented in detail in this section.

### MLS Measurement

Researchers have proposed various models to improve MLS estimation and can be classified into 2 categories: landmark-based approaches and DL-based approaches, where the former account for 25% of studies and the latter 75% (refer to Table S6 in Multimedia Appendix 3) [103-110].

**Figure 4.**
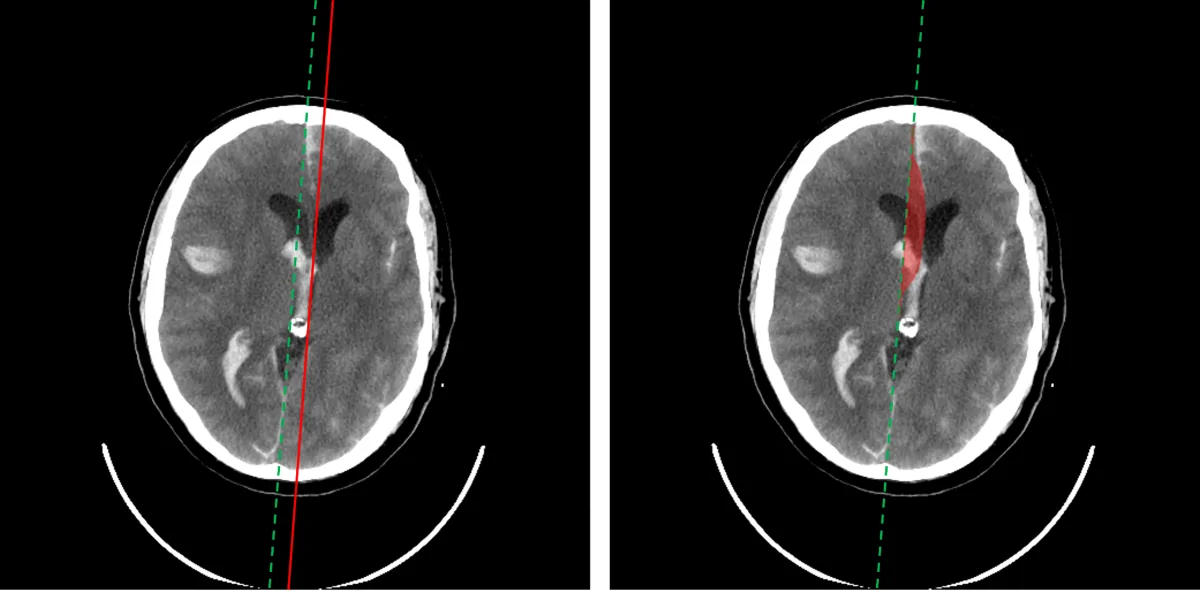
Visual comparison of automated linear midline shift and volumetric midsurface shift estimation techniques applied to a single noncontrast head computed tomography scan of a patient with traumatic brain injury. Adapted from Jiang et al [104], which is published under Creative Commons Attribution 4.0 International License [111], using a sample from the CQ500 dataset.

Landmark-based models rely on anatomical cues and geometric relationships. Hooshmand et al [103] developed a system that first selects a number of slices among all the slices in a CT scan based on metadata, as well as information extracted from the images such as the number of separate objects counted in that slice and the area of skull in comparison to brain tissue. Then, the line that divides the slices into 2 equal right and left partitions is marked as iML. Finally, they performed ventricles segmentation to detect the actual midline (aML). The aML is defined as the line that passes between the left and right lateral ventricles or through the third ventricle. MLS is then calculated as the distance between the iML and aML. Their model achieved moderate performance: 75% sensitivity, 65% specificity, 53% precision, 68% accuracy, and an *F*_1_-score of 0.62. However, this system focuses only on linear displacement and does not account for variations in tissue shape or volume. To address these limitations, Jiang et al [104] introduced a volumetric approach by proposing the midsurface shift (MSS). Figure 4 visualizes the difference between MLS and MSS estimations. MSS is defined as the ratio of midsurface volume to total brain volume, calculated from selected CT slices in a CT scan. First, they used 3D Chan-Vese segmentation combined with an active contour on contrast-adjusted CT scans to segment the brain mask. The midsurface is then manually annotated using biomarkers such as the anterior, the falx cerebri, the middle of the ventricle, and the posterior. The iML is defined by the intersections of the midsurface curve and the inner edge of the skull, and the volume of shift is calculated based on this iML. Finally, the ratio between the volume of shift and the brain volume is computed. While conceptually innovative, this study has not yet been quantitatively validated.

Contrarily, DL-based models learn discriminative features directly from imaging data. Wei et al [105] developed a regression-based line detection network that incorporates multiscale line detection, weighted line integration, and regression-based line refinement. Their method formulated the task of identifying the brain’s midline structure as a skeleton extraction problem and used a multiscale approach to capture both high-level semantic and low-level detailed information, along with a regression-based refinement. They achieved a mean line distance error of 1.17 (SD 0.72) mm and an *F*_1_-score of 0.78 on the CQ500 dataset. However, testing on a private dataset resulted in a larger mean error of 4.15 (SD 3.97) mm and a lower *F*_1_-score of 0.61, underscoring challenges in generalization across datasets. Yan et al [106] introduce a modified Keypoint R-CNN framework trained on a ResNet-FPN-50 backbone to detect 4 keypoints: (1) the anterior falx, (2) the posterior falx, (3) the anterior septum pellucidum near anterior commissure, and (4) the posterior septum pellucidum. The MLS is then calculated by the distance between the middle point of the connection of the anterior and posterior falx and the midpoint of the septum pellucidum. Their method reported a mean absolute error of 0.936 mm, sensitivity of 91.7%, specificity of 97.4%, and an intraclass correlation coefficient of 0.9899 compared to clinician assessments. This close agreement with expert evaluations positions their approach as highly competitive in clinical settings. Xia et al [107] designed a CNN framework with three stages: (1) U-Net for image alignment, (2) U-Net for midline segmentation, and (3) dynamic programming for pathfinding. The MLS is estimated by calculating the distance of midline shift (MLS-D) and the volume of midline shift (MLS-V). The horizontal distances between the aML and the iML are calculated on all shifted slices, with the largest distance chosen as the MLS-D. The MLS-V is calculated as the number of voxels between the aMLs and iMLs multiplied by the physical size of a voxel, whereby a voxel is defined as a discrete element of volume across the x, y, and z axes (x-spacing×y-spacing×z-spacing). They achieved a mean shift distance error of 1.14 (SD 0.91) mm and a mean shift area error of 0.88 (SD 0.79) cm^2^, with AUC-ROC scores of 0.799 and 0.831 for MLS-D and MLS-V, respectively. Nag et al [108] also applied a U-Net–based approach to segment the hemispheres and define the aML based on boundary detection, estimating the iML by connecting the top-most and bottom-most points of the aML. They achieved a validation accuracy of 94.05%, with a pixel error of 1.294 mm, an area error of 66.4 mm^2^, and a volume error of 253.73 mm^3^. Similarly, Wu et al [109] proposed a hemisphere-segmentation framework based on a 3D U-Net architecture. This network is divided into three components: (1) a hemisphere-segmentation network, (2) rectification learning, and (3) midline correction. They achieved an average surface distance of 0.84 (SD 0.4) mm and a mean Hausdorff distance of 9.1±7.5 mm on the CQ500 dataset.

### ICP Estimation

Recently, several noninvasive methods for estimating ICP have been developed. However, the body of research in this area remains relatively limited, accounting for only about 2.9% of the reviewed literature. Pappu et al [112] proposed a semiautomatic technique that isolates brain tissue from cerebrospinal fluid (CSF) on CT scans. Each pixel is assigned an indicator value for CSF, blood, or brain tissue. The algorithm calculates the ratio of cerebrospinal fluid volume (CSFV) to the intracranial vault volume (ICVV). These computed ratios (CSFV/ICVV) were plotted against the recorded ICP values. Statistical analysis was performed using the Statistics Toolbox in MATLAB. They found that a CSFV/ICVV ratio exceeding 0.034 correlated with an ICP below 20 mm Hg (*P*=.005). Based on this finding, they adopted 0.034 as a threshold to indicate elevated ICP, achieving a predictive accuracy of approximately 67%, indicating that further refinement is needed. Shan et al [113] investigated 3 predictive models using different types of input features: a HU model using HU-related imaging features, an MLS model relying on MLS data, and a clinical expertise (CE) model correlating clinical judgment with the actual ICP value. These models were evaluated for both binary classifications, indicating an increased ICP with a yes or no, and multiclass classification for ICP thresholds ≤22, 23‐29, and ≥30 mm Hg. For the binary classification task, the HU model outperformed the others, achieving an accuracy of 81% and an *F*_1_-score of 0.85. In contrast, the MLS and CE models had lower accuracies of 61.7% and 63.83%, respectively. When extended to the more complex multiclass task, all models experienced performance declines. The HU model’s accuracy dropped to 61.7%, and the MLS model to 40.43%. Most notably, the CE model failed to provide reliable classifications. This decline likely reflects difficulties in distinguishing subtle differences in imaging features that correspond to different increased ICP severities. Li et al [114] introduced a radiomics-based approach that leverages quantitative feature extraction from CT scans. By using PyRadiomics, the study extracted 18 first-order and 40 second-order features. First-order features capture basic statistics of voxel intensities within a RoI, reflecting the overall brightness or density of brain tissue or CSF, including mean, SD, and skewness. In contrast, second-order features describe the spatial relationships between pixel intensities, commonly known as texture features, and are derived using the gray-level co-occurrence matrices or the gray-level run-length matrix. Feature selection was performed using the least absolute shrinkage and selection operator, and 3 models were developed: a clinical features model, a first-order model, and a second-order model. The SO model yielded the best performance, with an accuracy of 80%, an *F*_1_-score of 0.83, and an AUC-ROC of 0.81. This finding highlights the potential of incorporating higher-order radiomic information to enhance predictive accuracy. Table S7 in Multimedia Appendix 3 [112-114] presents the details of the included studies.

## Discussion

### Principal Findings

Based on our survey, we identified numerous challenges in the current research, limiting AI for TBI application in real-world settings. First, current AI models are designed to detect isolated radiological findings such as ICH, MLS, and elevated ICP. While these targeted approaches have demonstrated high accuracy for individual tasks, they fail to capture the broader heterogeneity of TBI presentations. For instance, increased ICP is typically not an isolated finding, but often results from a combination of factors such as brain lesions, MLS, and compression of the basal cisterns. However, current studies rarely consider these interrelated indicators. This narrow, fragmented approach limits diagnostic performance in real-world settings, where multiple abnormalities commonly occur together. To address these limitations, future research should aim to develop integrated AI systems that combine multiple models into a unified diagnostic pipeline capable of recognizing the full spectrum of TBI-related abnormalities. One promising direction is the use of hierarchical AI frameworks, which incorporate specialized modules for different lesion types and severity levels. Such frameworks could be more context-aware decision support systems.

Second, clinical validation of these AI models is critically lacking. Among the reviewed literature, only one study [106] conducted formal validation involving health care professionals. In the context of this review, “formal validation involving health care professionals” means having clinicians directly involved in validating the performance of the models by actively evaluating the model’s outputs or having independent evaluation of the model’s output against targeted assessments made by clinical experts. Without such targeted clinical validation, model performance metrics, however strong, remain theoretical. Moreover, translation of AI-based TBI assessment tools into clinical practice requires substantially more evidence than is currently available in most studies. Regulatory approval pathways for AI-based diagnostic systems, such as Software as a Medical Device, typically require prospective validation, demonstration of generalizability across sites and scanners, clearly defined clinical use cases, and assessment of human-AI interaction within clinical workflows. The limitation in external clinical validation highlights a gap between methodological development and regulatory or clinical readiness. Addressing these requirements will be essential for future studies aiming to move beyond proof-of-concept toward deployable clinical systems. Thus, it is essential that future AI development be conducted in close collaboration with clinicians to ensure that AI tools not only have consistent technical performance but also have alignment with clinical expectations.

Third, the condition of the basal cisterns such as normal, compressed, or absent is a known prognostic indicator in TBI [115-117] but remains underexplored in AI literature. This gap likely exists due to the subjective nature of interpreting basal cistern conditions, making it difficult to establish clear criteria for training ML models. Similarly, AI-based ICP estimation is another underexplored area, with only 2.9% of the reviewed studies addressing it. Current methods remain insufficiently robust for clinical use. Future studies should investigate the potential for integrating multimodal data, combining imaging and physiological signals to improve the accuracy of ICP estimation.

Furthermore, there is a notable challenge in validating and comparing these AI systems. This is primarily due to the limited availability of publicly accessible benchmark datasets for TBI, especially extensive annotated datasets from clinical experts. Most studies reviewed in this paper relied on private datasets with limited access, which impedes efforts to benchmark segmentation algorithms with consistent training and testing data. The PhysioNet ICH dataset, which includes CT scans from 82 patients with TBI with pixel-level annotations, is currently the only publicly available resource for segmentation tasks. However, the small size of this dataset poses a risk of overfitting when training complex DL models with millions of parameters. To advance the field, there is a pressing need for multi-institutional collaborations to build large, diverse, and publicly available datasets with standardized annotations by expert clinicians. Such resources would not only enable fair comparison of algorithms but also foster the development of clinically viable models.

In addition to the need for larger datasets, AI models for TBI assessments are currently limited by their dependence on supervised learning techniques, which require large amounts of annotated data. This is particularly challenging in the medical domain, where TBI lesion annotations must be performed by clinicians with specialized knowledge. Furthermore, given CT scans represent 3D volumetric data, the annotation process is time-consuming and complex. To alleviate this burden, future research should explore alternative learning strategies, such as semi-supervised or self-supervised learning techniques, which can reduce the reliance on extensive annotated datasets. The study of Yao et al [118] is one of the few attempts to address this issue.

Finally, one of the most significant limitations of AI models in health care applications is their “black box” nature, which limits transparency and interpretability. To improve clinical acceptance and facilitate integration into TBI assessment, AI models should ideally be interpretable and aligned with clinical domain knowledge. Rule-based models, particularly those using fuzzy neural networks (FNN), are a promising solution. FNNs allow the extraction of interpretable “if-then” rules, which can be aligned with CE. This approach not only improves model performance but also reduces the need for large volumes of training data. Additionally, FNNs offer the flexibility to extract meaningful rules even when some variables are missing, making them particularly suitable for real-world clinical environments where data may be incomplete.

### Limitations

This review has several limitations. First, as a systematized review, it lacks prospective protocol registration, duplicate independent screening, and formal risk-of-bias assessment using standardized instruments. Furthermore, our highly aggressive exclusion threshold during the initial title and abstract screening phase, which resulted in zero exclusions at the full-text review stage, carries an inherent risk of selection bias. Relevant studies that failed to explicitly report specific inclusion criteria (such as imaging modality or patient age) in their abstracts may have been prematurely excluded. Although we qualitatively considered key sources of bias such as dataset size, single-center versus multicenter design, annotation practices, and validation strategies, our ability to systematically quantify study quality was limited, which may increase the risk of selection and reporting bias.

Second, the evidence base is constrained by heterogeneous study designs, outcome definitions, and reporting practices, which precluded meta-analysis and may introduce selective reporting and publication bias; for example, negative or inconclusive AI studies may be underrepresented in the published literature. Consequently, the findings should not be interpreted as definitive estimates of diagnostic accuracy or clinical effectiveness. Instead, the primary contribution of this review is to provide a structured mapping of AI tasks, methodological approaches, datasets, and evaluation practices used in CT-based TBI assessment. The review is intended to highlight methodological trends, recurring limitations, and gaps in validation and reporting, rather than to support direct clinical adoption or comparative performance claims between models.

Third, we restricted inclusion to English-language, peer-reviewed papers indexed in 4 major databases and did not systematically search gray literature, potentially missing relevant work. The review was restricted to English-language publications to ensure reliable interpretation of technical and methodological details, as incomplete or inaccurate translation could compromise assessment consistency. Moreover, peer-reviewed publications were prioritized to reduce the risk of incomplete, preliminary, or subsequently revised methodological claims.

Fourth, despite updating the literature search to include recent publications, the rapid pace of research in AI for medical imaging means that newly emerging methods may continue to appear after the search cutoff.

Finally, although multimodal AI approaches integrating CT imaging with clinical or physiological data are increasingly explored, they were excluded from this review by design to maintain a focused analysis of imaging-specific methodologies. Such approaches address distinct methodological and clinical questions related to outcome prediction rather than isolated imaging-based abnormality detection. Therefore, the lack of multimodal analysis in this study represents a limitation of our review scope, rather than a deficit in the broader scientific field. A future dedicated review focusing exclusively on multimodal TBI AI systems, as well as distinct reviews tailored to pediatric neurotrauma, are critically needed to complement these findings.

### Conclusions

We have highlighted the progress made in the application of AI for TBI assessment on CT scans. Despite advances in research to improve the performance of AI models in TBI assessment, several key challenges must be addressed to improve deploying these models into clinical workflows in real-world settings. These challenges include the need for more inclusive models that address clinical TBI conditions [119], as well as the development of large-scale, publicly available datasets to mitigate overfitting and ensure generalization. Most importantly, a gap in the literature is the annotation of data. To improve the development of these models, we need to explore alternative learning strategies to efficiently reduce annotation burdens. Given clinicians have the expertise needed for accurate annotation, their clinical commitment leaves minimal time for labeling data. Furthermore, ensuring transparency and interpretability of AI models is essential for successful integration into clinical practice and clinical acceptance. Finally, clinical validation with health care professionals is critical to confirm the practical utility and reliability of AI-based TBI diagnostic tools. By addressing these challenges, AI can become a more effective tool in TBI assessment.

## Supplementary material

10.2196/87794 Multimedia Appendix 1 Comparison of diagnostic imaging and assessment tools for suspected traumatic brain injury in critical care settings, detailing the invasiveness, turnaround time, cost, accessibility, and clinical utility of each modality.

10.2196/87794 Multimedia Appendix 2 Detailed database search queries and predefined inclusion and exclusion criteria applied during the systematized review to identify original peer-reviewed studies evaluating AI algorithms applied to noncontrast head computed tomography scans in adult patients with traumatic brain injury.

10.2196/87794 Multimedia Appendix 3 Search results detailing the study characteristics, algorithmic frameworks, and performance metrics of the reviewed literature.

10.2196/87794 Multimedia Appendix 4 Data extraction form.

10.2196/87794 Checklist 1 PRISMA checklist.

## Abbreviations

aML: actual midline
ANN: artificial neural network
AUC-ROC: area under the receiver operating characteristic curve
Bi-LSTM: bidirectional long short-term memory
CAM: cascaded attention module
CE: clinical expertise
CNN: convolutional neural network
CSF: cerebrospinal fluid
CSFV: cerebrospinal fluid volume
CT: computed tomography
DL: deep learning
EDH: epidural hematoma
EHR: electronic health record
FITBIR: Federal Interagency Traumatic Brain Injury Research Informatics System
FNN: fuzzy neural network
FPN: feature pyramid network
FRM: feature refinement module
GCS: Glasgow Coma Scale
GRADE: Grading of Recommendations Assessment, Development and Evaluation
HU: Hounsfield unit
ICH: intracranial hemorrhage
ICP: intracranial pressure
ICVV: intracranial vault
iML: ideal midline
IPH: intraparenchymal hemorrhage
IVH: intraventricular hemorrhage
LSTM: long short-term memory
MIMIC-III: Medical Information Mart for Intensive Care III
ML: machine learning
MLS: midline shift
MLS-D: distance of midline shift
MLS-V: volume of midline shift
MRI: magnetic resonance imaging
MSS: midsurface shift
MVEDL: Majority Voting Ensemble Deep Learning
NCA: neighborhood component analysis
PCR: pixel-wise correlation retaining
PET: positron emission tomography
PRISMA: Preferred Reporting Items for Systematic reviews and Meta-Analyses
PROBAST: Prediction Model Risk of Bias Assessment Tool
QUADAS-2: Quality Assessment of Diagnostic Accuracy Studies-2
RNN: recurrent neural network
RoI: region of interest
RSNA: Radiological Society of North America
SAH: subarachnoid hemorrhage
SDH: subdural hematoma
SE: squeeze-and-excitation
SGP: sparse Gaussian process
SPPF: spatial pyramid pooling—fast
TBI: traumatic brain injury
ViT: vision transformer
WCO: Willow Catkin Optimization
