# Supplementary material for: AI-Based Approaches for the Identification and Quantification of Traumatic Brain Injury in Computed Tomography Imaging: Systematic Review
Source: JMIR AI. 2026 Sep 10;5:e87794. doi: 10.2196/87794 (PMC13561197; doi:10.2196/87794)
Supplement: Supplementary file 1 [file ai-v5-e87794-s001.pdf]

Table S1. Comparison of diagnostic imaging and assessment tools for suspected TBI in critical care settings, detailing the invasiveness, turnaround time, cost, accessibility, and clinical utility of each modality.

	**Non-Invasive**	**Rapid Results**	**Cost**	**High Resolution Anatomical Imaging**	**Accessibility**	**Availability for Emergency Use**	**Contraindications**	**Clinical Utility**
**GCS**	✅	✅	Low (No required special equipment)	❌	High (available in all emergency settings)	✅	N/A	Initial assessment of consciousness, monitoring neurological status
**CT**	✅	✅	Moderate ($120K-$160K for an intermediate scanner)	✅	Moderate (available in widely available in hospitals)	✅	Radiation exposure, contrast allergies, pregnancy, diabetes, renal impairment	Rapid assessment of hemorrhage, skull fractures, edema
**MRI**	✅	❌	High ($500K - $700K for an intermediate scanner)	✅	Low (available in specialized centers)	❌	Implanted devices, metal objects, pregnancy, contrast allergies, claustrophobia, obesity	Preferred for soft tissue and subtle chronic injuries
**PET**	✅	❌	Very High ($1M-$2M for a scanner)	❌	Low (available in specialized centers)	❌	Radiation exposure, pregnancy, diabetes, claustrophobia, inability to remain still, obesity	Provides functional and metabolic information
**Ultrasound**	✅	✅	Low ($50K-80K for a machine)	❌	High (available in most clinical settings)	✅	Limited use in adults with normal skull thickness	Evaluates and monitors cerebral blood flow velocities in ICU settings, locates and evaluates tumor masses during brain surgery, useful in neonates and children
**Doppler Ultrasound**	✅	✅	Low	❌	High (available in most clinical settings)	✅	N/A	Assesses cerebral hemodynamics, checks on the risk of stroke
**EEG**	✅	❌	Low to Moderate	❌	Moderate (available in many large regional or urban hospitals)	❌ (limited use in non-ICU emergency settings)	Recent stroke, severe cardiovascular or respiratory disease, photophobia, inability to perform the procedure	Measures the brain's electrical activity, detects neurological disorders
