# Supplementary material for: AI-Based Approaches for the Identification and Quantification of Traumatic Brain Injury in Computed Tomography Imaging: Systematic Review
Source: JMIR AI. 2026 Sep 10;5:e87794. doi: 10.2196/87794 (PMC13561197; doi:10.2196/87794)
Supplement: Supplementary file 2 [file ai-v5-e87794-s002.pdf]

Table S1. Detailed database search queries utilized in the systematized review of AI approaches for CT-based TBI assessment, covering PubMed, Scopus, Web of Science, and IEEE Xplore for English-language peer-reviewed literature published from inception to December 31, 2025.

Database	Query
**PubMed**	("Brain Injuries, Traumatic"[MeSH Terms] OR "Cerebral Hemorrhage, Traumatic"[MeSH Terms]) AND ((traumatic AND brain AND injury) OR (traumatic AND brain AND injuries) OR (brain AND trauma) OR (brain AND traumas) OR (extradural AND hemorrhage) OR (extradural AND hematoma) OR (epidural AND hemorrhage) OR (epidural AND hematoma) OR (subdural AND hemorrhage) OR (subdural AND hematoma) OR (intraparenchymal AND hemorrhage) OR (intraparenchymal AND hematoma) OR (intracerebral AND hemorrhage) OR (intracerebral AND hematoma) OR ICH OR (midline AND shift) OR (increased AND intracranial AND pressure) OR ICP) AND ((computerized AND tomography) OR CT) AND (((deep OR convolutional OR neural) AND network) OR (deep AND learning) OR (machine AND learning) OR (artificial AND intelligence) OR AI) NOT ((brain AND tumor) OR stroke OR (toxic AND injury) OR (cerebral AND edema) OR (anoxic AND injury) OR aneurysm OR (drug AND abuse) OR (heart AND attack) OR encephalitis OR anoxia OR hydrocephalus OR meningitis OR (magnetic AND resonance) OR MRI OR electroencephalogram OR EEG OR (positron AND emission AND tomography) OR PET OR (single AND photon AND emission AND computed AND tomography) OR SPECT OR ultrasound) NOT Review[ptyp] AND English[lang]
**Scopus**	TITLE-ABS-KEY (((brain AND trauma) OR (brain AND traumas) OR (traumatic AND brain AND injury) OR (traumatic AND brain AND injuries) OR (extradural AND hemorrhage) OR (extradural AND hematoma) OR (epidural AND hemorrhage) OR (epidural AND hematoma) OR (subdural AND hemorrhage) OR (subdural AND hematoma) OR (intraparenchymal AND hemorrhage) OR (intraparenchymal AND hematoma) OR (intracerebral AND hemorrhage) OR (intracerebral AND hematoma) OR ich OR (midline AND shift) OR (increased AND intracranial AND pressure) OR icp) AND ((computerized AND tomography) OR ct) AND (((deep OR convolutional OR neural) AND network) OR (deep AND learning) OR (machine AND learning) OR (artificial AND intelligence) OR ai) AND NOT ((brain AND tumor) OR stroke OR (toxic AND injury) OR (cerebral AND edema) OR (anoxic AND injury) OR aneurysm OR (drug AND abuse) OR (heart AND attack) OR encephalitis OR anoxia OR hydrocephalus OR meningitis OR (magnetic AND resonance) OR mri OR electroencephalogram OR eeg OR (positron AND emission AND tomography) OR pet OR (single AND photon AND emission AND computed AND tomography) OR spect OR ultrasound)) AND PUBYEAR < 2026 AND (LIMIT-TO (DOCTYPE , "ar") OR LIMIT-TO (DOCTYPE , "cp")) AND (LIMIT-TO (LANGUAGE , "English"))
**Web of Science**	TS=(((brain AND trauma) OR (brain AND traumas) OR (traumatic AND brain AND injury) OR (traumatic AND brain AND injuries) OR (extradural AND hemorrhage) OR (extradural AND hematoma) OR (epidural AND hemorrhage) OR (epidural AND hematoma) OR (subdural AND hemorrhage) OR (subdural AND hematoma) OR (intraparenchymal AND hemorrhage) OR (intraparenchymal AND hematoma) OR (intracerebral AND hemorrhage) OR (intracerebral AND hematoma) OR ich OR (midline AND shift) OR (increased AND intracranial AND pressure) OR icp) AND ((computerized AND tomography) OR ct) AND (((deep OR convolutional OR neural) AND network) OR (deep AND learning) OR (machine AND learning) OR (artificial AND intelligence) OR ai) NOT ((brain AND tumor) OR stroke OR (toxic AND injury) OR (cerebral AND edema) OR (anoxic AND injury) OR aneurysm OR (drug AND abuse) OR (heart AND attack) OR encephalitis OR anoxia OR hydrocephalus OR meningitis OR (magnetic AND resonance) OR mri OR

	electroencephalogram OR eeg OR (positron AND emission AND tomography) OR pet OR (single AND photon AND emission AND computed AND tomography) OR spect OR ultrasound)) AND LA=English AND DT=("Article" OR "Proceedings Paper")
**IEEE Xplore**	((("Mesh_Terms": "Brain Injuries, Traumatic") OR ("Mesh_Terms": "Cerebral Hemorrhage, Traumatic")) AND (((brain AND trauma) OR (brain AND traumas) OR (traumatic AND brain AND injury) OR (traumatic AND brain AND injuries) OR (extradural AND hemorrhage) OR (extradural AND hematoma) OR (epidural AND hemorrhage) OR (epidural AND hematoma) OR (subdural AND hemorrhage) OR (subdural AND hematoma) OR (intraparenchymal AND hemorrhage) OR (intraparenchymal AND hematoma) OR (intracerebral AND hemorrhage) OR (intracerebral AND hematoma) OR ich OR (midline AND shift) OR (increased AND intracranial AND pressure) OR icp) AND ((computerized AND tomography) OR ct) AND (((deep OR convolutional OR neural) AND network) OR (deep AND learning) OR (machine AND learning) OR (artificial AND intelligence) OR ai) AND NOT ((brain AND tumor) OR stroke OR (toxic AND injury) OR (cerebral AND edema) OR (anoxic AND injury) OR aneurysm OR (drug AND abuse) OR (heart AND attack) OR encephalitis OR anoxia OR hydrocephalus OR meningitis OR (magnetic AND resonance) OR mri OR electroencephalogram OR eeg OR (positron AND emission AND tomography) OR pet OR (single AND photon AND emission AND computed AND tomography) OR spect OR ultrasound)))

Table S2. Predefined inclusion and exclusion criteria applied during the systematized review to identify original peer-reviewed studies evaluating artificial intelligence algorithms applied to non-contrast head CT scans in adult TBI patients.

IC1	The study involves the development of AI algorithms for TBI assessment (e.g., hemorrhage segmentation, midline shift measurement, or ICP estimation).
IC2	The study uses brain CT scans as input data.
IC3	The publication is a peer-reviewed journal article or conference paper.
EC1	The study is a duplicate.
EC2	The publication has been retracted.
EC3	The study uses data modalities other than CT scans.
EC4	The study is not related to TBI or focuses on unrelated conditions (e.g., stroke, brain tumors, or other neurological disorders).
EC5	The study focuses on infants or pediatric populations.
EC6	The study is not published in English.
EC7	The study is not original research (e.g., reviews, meta-analyses, or secondary analyses).
EC8	The study does not propose an AI algorithm (e.g., focuses solely on statistical analyses, data processing, data augmentation, therapeutic techniques, or pathological investigations)
EC9	The study reports non-relevant outcomes (e.g., hematoma expansion, patient outcomes, prognosis, or other risk prediction tasks).
