# Supplementary material for: AI-Based Approaches for the Identification and Quantification of Traumatic Brain Injury in Computed Tomography Imaging: Systematic Review
Source: JMIR AI. 2026 Sep 10;5:e87794. doi: 10.2196/87794 (PMC13561197; doi:10.2196/87794)
Supplement: Supplementary file 3 [file ai-v5-e87794-s003.pdf]

Table S1. Summary of artificial intelligence (AI) models and performance metrics for the binary classification and screening (presence/absence) of traumatic brain injury (TBI) in non-contrast head computed tomography (CT) scans of adult patients, identified through a systematized review from database inception to 31 December 2025.

Study	Model	Dataset	Data Size	Classification	Performances
Dusenberry et al. [46]	ANN	PRIVATE DATASET	514 patients	Presence of acute CT findings / Not presence of acute CT findings	Accuracy = 93.14%; Precision = 88.00%; Recall = 97.78%; Specificity = 89.47%; NPV = 98.08%
Gan et al. [47]	ResNet + SE + LSTM, Transfer learning with ImageNet pretrained weights	PRIVATE DATASET	226 patients	TBI / No TBI	Accuracy = 95.9%; Sensitivity = 93.3%; Specificity = 98.9%
Zhang et al. [48]	VGG-SE-PCR	PRIVATE DATASET	636 images	TBI / No TBI	Accuracy = 89.3%; Sensitivity = 98.6%; Specificity = 82.9%
Ellethy et al. [49]	Multi-modal architecture based on RCNN model, with an additional branch for clinical data and an infusion layer to merge CT features with clinical features	TRACK-TBI	296 patients	Normal / Mild TBI	Accuracy = 82.4% ± 1.7%; Sensitivity = 82.6% ± 5.1%; Specificity = 81.6% ± 5.4%

Table S2. Summary of artificial intelligence (AI) models and performance metrics for the binary detection of intracranial hemorrhage (presence/absence) in non-contrast head computed tomography (CT) scans of adult patients with traumatic brain injury (TBI), identified through a systematized review from database inception to 31 December 2025.

Study	Model	Dataset	Data Size	Performances
Sindhura et al. [51]	ITSS + cascaded CNN-RNN	RSNA	361,838 slices	Accuracy = 95.50%;<br>Sensitivity = 93.16%;<br>Specificity = 97.10%
Pérez-Cano et al. [55]	E2E-Att-GP, E2E-GP-Att	- RSNA (training)<br><br>- CQ500 (testing)	- Training: 674,258 images from 19,530 studies<br><br>- Testing: 491 patients	**RSNA:**<br>**E2E-Att-GP**<br>Accuracy = 87.6% ± 0.023 ;<br>F1-Score = 0.886 ± 0.011;<br>Precision = 82.5% ± 0.032;<br>Recall = 95.9% ± 0.025;<br>AUC-ROC = 0.965 ± 0.007<br><br>**E2E-GP-Att**<br>Accuracy = 85.6% ± 0.021 ;<br>F1-Score = 0.879 ± 0.015;<br>Precision = 82.3% ± 0.041;<br>Recall = 94.4% ± 0.022;<br>AUC-ROC = 0.964 ± 0.005<br><br>**CQ500**<br>**E2E-Att-GP**<br>Accuracy = 79.6% ± 0.018 ;<br>F1-Score = 0.785 ± 0.017;<br>AUC-ROC = 0.918 ± 0.003;<br>Cohen's kappa = 0.594 ± 0.035<br><br>**E2E-GP-Att**<br>Accuracy = 75.6% ± 0.023 ;<br>F1-Score = 0.736 ± 0.031;<br>AUC-ROC = 0.912 ± 0.003;<br>Cohen's kappa = 0.509 ± 0.048
Kar et al. [54]	CNN + attention mechanisms	PhysioNet	82 patients	Accuracy = 99.76%;<br>Precision = 99.53%;

				Recall = 99.68%;<br>F1-Score = 0.9961;<br>AUC-ROC = 0.99
Fan et al. [50]	DTViT	PRIVATE DATASET	15,936 slices	Accuracy = 99.68%;<br>Precision = 99.35%;<br>Recall = 99.78%;<br>F1-Score = 0.9957;<br>Specificity = 99.62%
Wu et al. [57]	Weakly supervised deep learning architecture: EfficientNet-B2 + attention-based bidirectional LSTM + transfer learning	- RSNA (training)<br><br>- CQ500 (testing)<br><br>- PRIVATE DATASET (fine-tuning): 2008–2012 non-contrast head CT scans performed at Northwestern Memorial Hospital, Chicago.	- Training: 674,258 images from 19,530 studies<br><br>- Testing: 491 patients<br><br>- Fine-tuning: 10,699 scans (7469 patients)	**Held-out Local Test:**<br>Accuracy = 93.7%;<br>Sensitivity = 84.1%;<br>Specificity = 96.3%;<br>NPV = 0.958;<br>AUC-ROC = 0.96;<br>Positive Predictive Value (PPV) = 0.857;<br>F1-Score = 0.849;<br><br>**External Test (CQ500):**<br>Accuracy = 91.6%;<br>Sensitivity = 88.5%;<br>Specificity = 89.6%;<br>NPV = 0.898;<br>AUC-ROC = 0.96;<br>PPV = 0.893;<br>F1-Score = 0.89;
Agrawal et al. [53]	3D CNN model	PRIVATE DATASET:<br>CT scans were collected prospectively from the Department of Neurosurgery, All India Institute of Medical Sciences, New Delhi from January 2021 to September 2021.	200 scans (training);<br>20 scans (test)	Accuracy = 80%;<br>Sensitivity = 90%;<br>Specificity = 70%
Pérez et al. [56]	DGPMIL: Att-CNN + Gaussian Processes	- Training: RSNA	- Training: 39,750 slices	**RSNA**<br>Accuracy = 82.5% ± 0.006;

		- Testing: CQ500	- Testing: 491 scans	F1-Score = 0.839 ± 0.006;<br>Cohen's kappa = 0.654± 0.011;<br>AUC-ROC = 0.957 ± 0.011;<br>Area under the (precision-recall) curve (AUC-PR) = 0.961 ± 0.011<br><br>**CQ500**<br>Accuracy = 71.7% ± 0.035;<br>F1-Score = 0.735 ± 0.022;<br>Cohen's kappa = 0.461± 0.059;<br>AUC-ROC = 0.909 ± 0.005;<br>AUC-PR = 0.889 ± 0.011
Ganeshkumar et al. [39]	CycleGAN + ResNet50	PhysioNet	82 scans	Sensitivity = 80%;<br>Specificity = 99%;<br>Accuracy = 96%;<br>AUC-ROC = 0.87;<br>F1-Score = 0.91
Altuve and Pérez [52]	Pre-trained ResNet-18 + transfer learning	Kaggle's Head CT-Hemorrhage DB	200 images	Accuracy = 95.93%;<br>Specificity = 96.20%;<br>Sensitivity = 95.65%;<br>Precision = 96.40%;<br>F1-Score = 0.9591

Table S3. Summary of artificial intelligence (AI) models and performance metrics for the multi-class classification of specific intracranial hemorrhage subtypes (e.g., epidural, subdural, subarachnoid, intraparenchymal) in non-contrast head computed tomography (CT) scans of adult patients with traumatic brain injury (TBI), identified through a systematized review from database inception to 31 December 2025.

Study	Model	Dataset	Data Size	Performances
Malik and Vidyarthi [58]	Ensemble: SE-ResNeXt-50 + EfficientNet B3 + EfficientNet B2	RSNA	874,035 slices	Accuracy = 98.56%;<br>Sensitivity = 95.89%;<br>Specificity = 99.58%;<br>AUC-ROC = 0.9847;<br>Precision = 98.3%;<br>Recall = 96.4%;<br>F1-Score = 0.985;<br>Kappa = 0.9
Gudadhe and Thakare [59]	WA2D-CNN	PUBLIC DATASET (from Kaggle)	36 patients (2510 images)	**Accuracy = 95.86%**<br><br>**ICH**<br>Precision = 89.52%;<br>Sensitivity = 88.68%;<br>F1-Score = 0.891<br><br>**SAH**<br>Precision = 96.34%;<br>Sensitivity = 95.18%;<br>F1-Score = 0.9576<br><br>**EDH**<br>Precision = 95.17%;<br>Sensitivity = 96.17%;<br>F1-Score = 0.9567<br><br>**Normal**<br>Precision = 96.15%;<br>Sensitivity = 92.59%;<br>F1-Score = 0.9454

Gencturk et al. [60]	Hybrid: MS R-CNN (ResNet-101+FPN backbone) + EfficientNet-B2	- CQ500<br><br>- PRIVATE DATASET: Collected at Niğde Ömer Halisdemir University Faculty of Medicine Neurosurgery Clinic and Cumhuriyet University Faculty of Medicine Neurosurgery Clinic	- CQ500: 491 scans<br><br>- Private dataset: 2434 images	**CQ500**<br>Accuracy = 94.301%;<br>Precision = 90.426%;<br>Recall = 97.701%;<br>F1-Score = 0.9392<br><br>**PRIVATE DATASET**<br>Accuracy = 97.331%;<br>Precision = 96.914%;<br>Recall = 99.054%;<br>F1-Score = 0.9797
Faiz et al. [67]	ViT	Refined RNSA CT Scans 12K	12,000 images	Accuracy = 81.2%
Umapathy et al. [61]	Hybrid: SE-ResNeXT + LSTM	- RSNA<br><br>- CQ500	- RSNA: 133,709 slices (train) + 14,600 slices (test)<br><br>- CQ500: 491 scans	Accuracy = 94%;<br>Precision = 96%;<br>Recall = 98%;<br>F1-Score = 0.97;<br>AUC-ROC = 0.99
Sengupta et al. [65]	- Region Segmentation: Otsu's thresholding technique<br>- Hybrid feature extraction: Tamura features + GLTP<br>- Classification: Bi-LSTM	RSNA	25,272 images (870,301 slices)	F1-Score = 0.993;<br>Matthews' Correlation Coefficient (MCC) = 0.9912;<br>Sensitivity = 99.40%;<br>Accuracy = 80%;<br>Specificity = 99.48%
Rajagopal et al. [68]	Hybrid Conv-LSTM	RSNA	752,534 slices	Sensitivity = 93.87%;<br>Specificity = 96.45%;<br>Accuracy = 95.14%;<br>Precision = 95.21%
Ozaltin et al. [64]	OzNet	PhysioNet	82 patients	**Oznet-NCA-ANN (Best results)**<br>Accuracy = 99.58%;<br>Sensitivity = 99.44%;<br>Specificity = 99.86%;<br>Precision = 99.31%;<br>F1-Score = 0.9937;

				G-Mean = 0.9965;<br>AUC-ROC = 0.9999
Negm et al. [66]	ICHD-WCOVE	PhysioNet	82 patients	Accuracy = 98.45%;<br>Precision = 97.93%;<br>Sensitivity = 92.82%;<br>Specificity = 98.74%;<br>F1-Score = 0.9514
Liu et al. [69]	Hybrid: CNN (ResNet50 & DenseNet121) + Bi-LSTM	RSNA	25,272 scans (870,301 slices)	Hamming Loss = 0.0348;<br>Accuracy = 87.22%;<br>Precision = 85.95%;<br>Recall = 77.76%;<br>F1-Score = 0.8165
Asif et al. [62]	Hybrid: ResNet101-V2 & Inception-V4 + LGBM	- Training: RSNA<br><br>- Testing: CQ500	- Training: 80,000 slices<br><br>- Testing: 491 scans	**Any**<br>AUC-ROC = 0.985;<br>Sensitivity = 95.4%;<br>Specificity = 97.2%;<br>Precision = 94.7%;<br>F1-Score = 0.963;<br>AUC-PR = 0.974;<br>Accuracy = 97.5%<br><br>**SDH**<br>AUC-ROC = 0.974;<br>Sensitivity = 96.5%;<br>Specificity = 98.3%;<br>Precision = 96.3%;<br>F1-Score = 0.972;<br>AUC-PR = 0.971;<br>Accuracy = 98.7%<br><br>**SAH**<br>AUC-ROC = 0.976;<br>Sensitivity = 96.2%;<br>Specificity = 97.3%;<br>Precision = 96.4%;

				F1-Score = 0.963;<br>AUC-PR = 0.972;<br>Accuracy = 98.5%<br><br>**EDH**<br>AUC-ROC = 0.980;<br>Sensitivity = 97.2%;<br>Specificity = 96.3%;<br>Precision = 96.6%;<br>F1-Score = 0.952;<br>AUC-PR = 0.964;<br>Accuracy = 97.5%<br><br>**IPH**<br>AUC-ROC = 0.979;<br>Sensitivity = 96.8%;<br>Specificity = 98.2%;<br>Precision = 97.3%;<br>F1-Score = 0.972;<br>AUC-PR = 0.965;<br>Accuracy = 98.7%<br><br>**IVH**<br>AUC-ROC = 0.977;<br>Sensitivity = 96.2%;<br>Specificity = 97.3%;<br>Precision = 97.2%;<br>F1-Score = 0.963;<br>AUC-PR = 0.968;<br>Accuracy = 98.5%
Akram et al. [63]	Hybrid: Double-branch Xception + Decision Tree	RSNA	- Training: 180,000 slices<br>- Validation: 12,000 slices	**EPH**<br>Accuracy = 99.73%;<br>Precision = 99.98%;<br>Recall = 97.01%;

			- Testing: 100,000 slices	F1-Score = 0.9847<br><br>**IPH**<br>Accuracy = 99.10%;<br>Precision = 96.01%;<br>Recall = 92.40%;<br>F1-Score = 0.9416<br><br>**IVH**<br>Accuracy = 99.49%;<br>Precision = 96.10%;<br>Recall = 90.01%;<br>F1-Score = 0.9295<br><br>**SAH**<br>Accuracy = 99.49%;<br>Precision = 96.10%;<br>Recall = 90.01%;<br>F1-Score = 0.9295<br><br>**SDH**<br>Accuracy = 98.09%;<br>Precision = 96.01%;<br>Recall = 94.04%;<br>F1-Score = 0.9501<br><br>**Any**<br>Accuracy = 97.35%;<br>Precision = 98.0%;<br>Recall = 84.76%;<br>F1-Score = 0.9089
Zhou et al. [70]	Transfer learning with ResNet-18 and DenseNet-121	PRIVATE DATASET: Patients at People's Hospital of Guangxi	2768 images	**ResNet-18**<br>**Normal**<br>Sensitivity = 1.00;<br>Specificity = 0.87;

		from March 2018 to March 2020		AUC-ROC = 1.00<br><br>**EDH**<br>Sensitivity = 0.98;<br>Specificity = 0.88;<br>AUC-ROC = 1.00<br><br>**IVH**<br>Sensitivity = 0.85;<br>Specificity = 0.91;<br>AUC-ROC = 0.96<br><br>**CPH**<br>Sensitivity = 0.8;<br>Specificity = 0.91;<br>AUC-ROC = 0.96<br><br>**SAH**<br>Sensitivity = 0.81;<br>Specificity = 0.91;<br>AUC-ROC = 0.94<br><br>**SDH**<br>Sensitivity = 0.93;<br>Specificity = 0.89;<br>AUC-ROC = 0.98<br><br>**DenseNet-121**<br>**Normal**<br>Sensitivity = 0.98;<br>Specificity = 0.79;<br>AUC-ROC = 1.00<br><br>**EDH**<br>Sensitivity = 0.86;

				Specificity = 0.81;<br>AUC-ROC = 0.97<br><br>**IVH**<br>Sensitivity = 0.73;<br>Specificity = 0.85;<br>AUC-ROC = 0.91<br><br>**CPH**<br>Sensitivity = 0.76;<br>Specificity = 0.84;<br>AUC-ROC = 0.94<br><br>**SAH**<br>Sensitivity = 0.81;<br>Specificity = 0.83;<br>AUC-ROC = 0.95<br><br>**SDH**<br>Sensitivity = 0.85;<br>Specificity = 0.82;<br>AUC-ROC = 0.94
Sengupta and Alzbutas [71]	OGRU-CSO:<br>- Integrating Fuzzy C Means (FCM) clustering for segmentation<br>- Hybrid feature extraction using Histogram of Oriented Gradients (HoG), Local Ternary Pattern (LTP), and Local Binary Pattern (LBP)<br>- Feature optimization with the Cuckoo Search Optimization (CSO) algorithm<br>- Classification with an Optimized Gated Recurrent Unit (OGRU)	RSNA	25,272 scans (870,301 slices)	Accuracy = 99.36%;<br>Recall = 99.25%;<br>Specificity = 99.40%

Phaphuangwittayakul et al. [72]	DeepMedic (3D CNN)	- RSNA<br><br>- PhysioNet<br><br>- PRIVATE DATASET: Patients were collected from Maharaj Nakorn Chiang Mai Hospital.	- RSNA: 75,5948 slices<br><br>- PhysioNet: 82 scans (2814 slices)<br><br>- Private dataset: 321 patients (19,946 slices)	Jaccard = 0.777 ± 0.403;<br>Dice = 0.784 ± 0.399;<br>Precision = 97.96%;<br>Sensitivity = 95.77%;<br>Specificity = 96.90%;<br>F1-Score = 0.9685;<br>Accuracy = 96.21%
Monica Jenefer et al. [73]	ICH-ResNet-DenseNet-IRF framework:<br>- Feature extraction: Multilayer DenseNet + ResNet<br>- Classification: Improved random forest (IRF)	PhysioNet	82 scans	**SDH**<br>Accuracy = 99.53%;<br>Specificity = 93.86%;<br>Sensitivity = 91.86%;<br>Precision = 96.44%;<br>Recall = 92.03%;<br>F1-Score = 0.9545;<br><br>**EDH**<br>Accuracy = 98.75%;<br>Specificity = 90.53%;<br>Sensitivity = 93.87%;<br>Precision = 95.61%;<br>Recall = 91.92%<br>F1-Score = 0.9486;<br><br>**IPH**<br>Accuracy = 98.09%;<br>Specificity = 91.75%;<br>Sensitivity = 90.45%;<br>Precision = 94.43%;<br>Recall = 93.92%;<br>F1-Score = 0.9264;

				**IVH**<br>Accuracy = 99.8%;<br>Specificity = 93.86%;<br>Sensitivity = 93.97%;<br>Precision = 97.96%;<br>Recall = 92.93%;<br>F1-Score = 0.9366;<br><br>**SAH**<br>Accuracy = 99.46%;<br>Specificity = 95.87%;<br>Sensitivity = 92.33%;<br>Precision = 94.66%;<br>Recall = 89.02%<br>F1-Score = 0.9065;
Korra et al. [74]	CNN	RSNA	25,272 scans (870,301 slices)	Accuracy = 98.63%;<br>Specificity = 99.49%;<br>Sensitivity = 73.32%;<br>AUC-ROC = 0.9898
Hussain et al. [38]	Hybrid: ResNet-152V2 + XGBoost	RSNA	25,272 scans (870,301 slices)	**EDH**<br>Accuracy = 99.2%;<br>Recall = 97.1%;<br>Specificity = 96.4%;<br>F1-Score =0.961;<br>AUC-ROC = 0.983<br><br>**IPH**<br>Accuracy = 97.1%;<br>Recall = 94.9%;<br>Specificity = 95.1%;<br>F1-Score = 0.943;<br>AUC-ROC = 0.929<br><br>**IVH**<br>Accuracy = 96.7%;

				Recall = 95.4%;<br>Specificity = 92.1%;<br>F1-Score = 0.927;<br>AUC-ROC = 0.941<br><br>**SDH**<br>Accuracy = 96.7%;<br>Recall = 96.4%;<br>Specificity = 91.4%;<br>F1-Score = 0.955;<br>AUC-ROC = 0.950<br><br>**SAH**<br>Accuracy = 96.1%;<br>Recall = 95.6%;<br>Specificity = 90.8%;<br>F1-Score = 0.921;<br>AUC-ROC = 0.953
Ertugrul and Akil [75]	YOLOv4	BHX	23,409 images (39,668 bounding boxes)	Precision = 94%;<br>Recall = 92%;<br>F1-Score = 0.93
Alis et al. [76]	CNN-RNN	PRIVATE DATASET	55,179 scans	**ICH**<br>Sensitivity = 99.70%;<br>Specificity = 99.34%;<br>Precision = 97.24%;<br>Accuracy = 99.41%;<br>AUC-ROC = 0.998<br><br>**IPH**<br>Sensitivity = 95.90%;<br>Specificity = 99.28%;<br>Precision = 95.03%;<br>Accuracy = 98.85%;<br>AUC-ROC = 0.998

				**IVH**<br>Sensitivity = 95.22%;<br>Specificity = 99.56%;<br>Precision = 94.99%;<br>Accuracy = 99.21%;<br>AUC-ROC = 0.998<br><br>**SAH**<br>Sensitivity = 84.50%;<br>Specificity = 99.09%;<br>Precision = 92.35%;<br>Accuracy = 97.41%;<br>AUC-ROC = 0.991<br><br>**SDH**<br>Sensitivity = 91.13%;<br>Specificity = 99.33%;<br>Precision = 92.78%;<br>Accuracy = 98.62%;<br>AUC-ROC = 0.974<br><br>**EDH**<br>Sensitivity = 74.61%;<br>Specificity = 98.83%;<br>Precision = 51.56%;<br>Accuracy = 98.50%;<br>AUC-ROC = 0.980

Table S4. Summary of artificial intelligence (AI) models and performance metrics for the spatial localization and classification of hemorrhages in non-contrast head computed tomography (CT) scans of adult patients with traumatic brain injury (TBI), identified through a systematized review from database inception to 31 December 2025.

Study	Model	Dataset	Data Size	Performances
Kothala and Guntur [77]	TL-LFF Network + GA	Brain Hemorrhage Extended (BHX) dataset (extension of the qure.ai CQ500 dataset)	491 patients (21,132 slices from 205 positive patients)	Specificity = 99.3%;<br>Precision = 93.5%;<br>Recall = 94.5%;<br>Accuracy = 98.7%;<br>False Negative Rate (FNR) = 0.005;<br>FNR = 0.02;<br>MCC = 0.968;<br>F1-Score = 0.940;<br>mAP@.5 = 0.964;<br>mAP@.5:.95 = 0.728
He et al. [78]	A multiscale feature classification and weakly supervised localization framework	PUBLIC DATASET: ASNR (Through Kaggle)	750,000 scans	AUC-ROC = 0.973
Vidhya et al. [79]	YOLOv5s-CAM	BHX Dataset (extended version of CQ500)	491 scans (39,668 bounding boxes)	Precision = 93.5%;<br>Recall = 90.8%;<br>F1-Score = 0.921;<br>mAP@.5 = 0.943;<br>mAP@.5:.95 = 0.65
Kothala et al. [80]	YOLOv5x-GCB	- BHX Dataset (extended version of CQ500)<br><br>- PRIVATE DATASET	- BHX dataset: 491 scans (205 positive, 21,132 slices)<br><br>- Private dataset: 75 patients (2,500 slices - 318 slices positive)	Precision = 92.1%;<br>Recall = 88.9%;<br>F1-Score = 0.900;<br>mAP@.5 = 0.931;<br>map@.5:.95 = 0.624
Cheng et al. [81]	Modified RetinaNet	N/A	N/A	mAP = 0.38<br><br>**ICH**<br>AUC-ROC = 0.956;

				AP = 0.46<br><br>**IVH**<br>AUC-ROC = 0.965;<br>AP = 0.17<br><br>**SDH**<br>AUC-ROC = 0.972;<br>AP = 0.49<br><br>**SAH**<br>AUC-ROC = 0.948;<br>AP = 0.41

Table S5. Summary of artificial intelligence (AI) models and performance metrics for the pixel-level segmentation and volumetric quantification of intracranial hemorrhages in non-contrast head computed tomography (CT) scans of adult patients with traumatic brain injury (TBI), identified through a systematized review from database inception to 31 December 2025.

Study	Model	Dataset	Data Size	Performances
Zhang et al. [82]	MOEL-Net	PRIVATE DATASET	460 CT scans + 480 CT scans	**Dataset 1:**<br>Dice = 0.9092;<br>Jaccard = 0.8367;<br>Precision = 92.46%;<br>Sensitivity = 90.02%;<br>Specificity = 99.97%<br>**Dataset 2:**<br>Dice = 0.9095;<br>Jaccard = 0.8360;<br>Precision = 93.43%;<br>Sensitivity = 88.95%;<br>Specificity = 99.97%
Zhang et al. [89]	Improved FCM algorithm	CT-ICH	256 images	Dice = 0.91;<br>RMSE = 0.097
Zhang et al. [93]	GPT-4	PRIVATE DATASET:<br>CT scans with 6 types of cerebral hemorrhages at Ren Ji Hospital, Shanghai Jiao Tong University School of Medicine, between January and September 2023	208 scans	**CP:**<br>epidural: 89.0%;<br>intraparenchymal: 86.9%;<br>acute subdural: 74.4%;<br>subarachnoid: 71.5%;<br>complex: 76.4%;<br>chronic subdural: 37.3%<br>**MP:**<br>epidural: 50.2%;<br>intraparenchymal: 26.2%;<br>acute subdural: 32.6%;<br>subarachnoid: 50.5%;<br>complex: 54%;<br>chronic subdural: 40.3%
Xu et al. [85]	Two-staged encoder-decoder CNN	PRIVATE DATASET	401 scans (train: 280 scans, test: 121 scans)	Dice = 0.8454;<br>Recall = 86.03%

Xiao et al. [83]	MPFR-Net	- PRIVATE DATASET<br><br>- PhysioNet (for evaluation)	- Private dataset: 18,386 slices<br><br>- PhysioNet: 82 patients	**Dice:**<br>IVH: 0.7721;<br>IPH: 0.8799;<br>ICH: 0.9074;<br>Avg = 0.8260
Xiao et al. [84]	DFMA-ICH	PRIVATE DATASET<br>Spontaneous ICH Dataset: 207 scans were retrospectively collected from Taihu Hospital in Wuxi, China (NCCT, 512x512 resolution, 5 mm layer thickness, 0.45-0.55 mm^2 pixel spacing); 100 scans were from the public INSTANCE 2022 challenge (NCCT) from Shougang Hospital, Peking University<br><br>Traumatic ICH Dataset: Comprised of 80 patients' CT data of traumatic ICH from Daping Hospital in Chongqing, China (512x512 resolution), including SDH and EDH subtypes	- Spontaneous ICH dataset: 307 scans<br><br>- Traumatic ICH dataset: 80 patients	**Spontaneous ICH Dataset:**<br>Dice = 0.8603 ± 0.1224;<br>Intersection over Union (IoU) = 0.7698 ± 0.1395;<br>Hausdorff Distance (HD) = 12.35mm ± 14.75mm;<br>Recall = 83.33% ± 13.98%;<br>F1-score = 0.8494 ± 0.1135<br>**Traumatic ICH Dataset:**<br>Dice = 0.8098 ± 0.1168;<br>IoU = 0.6991 ± 0.1514;<br>HD = 47.28mm ± 45.79mm;<br>Recall = 80.52% ± 13.36%;<br>F1-score = 0.8004 ± 0.1383
Nafees Ahmed and Prakasam [86]	U-Net architecture + CSR	PhysioNet	82 patients	**SDH**<br>Dice = 0.969;<br>IoU = 0.687;<br>Recall = 100%;<br>Precision = 100%;<br>F1-Score = 1.0;<br>Accuracy = 100%;<br>**EDH**<br>Dice = 0.983;

				IoU = 0.749;<br>Recall = 100%;<br>Precision = 100%;<br>F1-Score = 1.0;<br>Accuracy = 100%;
Rajapakse et al. [91]	YOLOv5 + TransDeepLab.	- CQ500<br><br>- RSNA	- CQ500: 491 scans (193,317 slices)<br><br>- RSNA: 874,035 slices	Dice = 0.605;<br>IoU = 0.478
Liu et al. [87]	DDANet	PhysioNet	82 patients	Dice = 0.712 ± 0.223;<br>Jaccard = 0.601 ± 0.240;<br>Sensitivity = 73.0% ± 0.210;<br>Specificity = 99.8% ± 0.002;<br>Accuracy = 99.7% ± 0.002
Lin and Yuh [92]	Semi-supervised learning paradigm with PatchFCN	- Atlantis-labeled dataset<br><br>- Kaggle-25K (unlabeled)<br><br>- PRIVATE DATASET (validation)<br><br>- CQ500 (testing)	- Atlantis: 457 scans<br><br>- Kaggle: 25K scans<br><br>- Private dataset: 93 scans<br><br>- CQ500: 491 scans	AUC-ROC = 0.939;<br>Dice = 0.829;<br>AP = 0.848
Li et al. [88]	REUnet	PRIVATE DATASET: ShanDong (SD) dataset: The data was collected between July 2013 and October 2020 at Qilu Hospital of Shandong University Dezhou Hospital<br><br>TianJin (TJ) dataset (testing): The data was collected between January 2015 and August 2019 at Tianjin Medical University General Hospital	- SD dataset: 977 images<br><br>- TJ dataset: 375 images	Dice = 0.777;<br>Sensitivity = 70.7%;<br>Precision = 91.0%;<br>Absolute Volume Difference (AVD) = 1.312;<br>Absolute Volume Difference Ratio (AVDR) = 0.229

Wang and Wang [96]	MSRL-Net – a modified U-Net structure:<br>- Using MSPooling (MaxPool + SoftPool) instead of just max-pooling<br>- Integrating Multi-Rate Hybrid Dilated Convolution (MRHDC) structure for mitigating the influence of image resolution reduction.	PRIVATE DATASET	82 scans	Dice = 0.717;<br>Precision = 71.2%;<br>Sensitivity = 77.9%;<br>Jaccard = 0.596
Liu et al. [97]	Viola-Unet:<br>- An asymmetric encoder-decoder U-Net with 7-depth layers.<br>- Viola attention module: Firstly, uses an adaptive average pooling (AdaAvgPool) module to squeeze the input feature volume into three latent representation spaces along each orthogonal axis (height, width, and depth). Then, customized dense dilated convolutions merging (DDCM) networks fuse cross-channel and non-local contextual information on each orthogonal direction with adaptive kernel sizes, dilated ratios, and strides. Finally, it constructs an attention volume based on the fused and reshaped latent representations, as described by mathematical equations involving Sigmoid, group normalization with Tanh, and ReLU activation functions, as well as tensor product and element-wise multiplication.	INSTANCE 2022 Challenge Dataset	200 scans	Dice = 0.7951 ± 0.171;<br>HD = 24.038 ± 29.236;<br>Normalized Surface Dice (NSD) = 0.5693 ± 0.125;<br>Relative Absolute Volume Difference (RVD = 0.1941 ± 0.179
Khan et al. [98]	Exploring various deep learning architectures, including U-Net, U-Net++, and FPN (Feature Pyramid	PhysioNet	82 scans	Dice = 0.85757;<br>IoU = 0.843;<br>Accuracy = 99.91%

	Network). These architectures were implemented with multiple pre-trained encoders (DenseNet121, DenseNet161, DenseNet201, ResNet18, ResNet152, InceptionV4, InceptionV2-ResNet) that were initially trained on the large-scale ImageNet dataset, utilizing the principle of transfer learning. The Densenet201_U-Net model achieved the best performance.			
Sharrock et al. [99]	Intracerebral intraVentricular Network (IV-Net):<br>- Based on the V-Net architecture<br>- Incorporating dilated convolutions for contextual propagation and multiclass attention-gating to focus learning on important context.<br>- Using Monte-Carlo Bernoulli dropout sampling to approximate uncertainty.	PRIVATE DATASET:<br>phase II (MISTIE II and CLEAR IVH) and phase III (MISTIE III and CLEAR III) clinical trials	1185 subjects	**MISTIE II**<br>Dice = 0.940;<br>Volume Correlation = 0.995;<br>RMSE = 2.22;<br>AVD = 0.655<br>**CLEAR IVH**<br>Dice = 0.905;<br>Volume Correlation = 0.995;<br>RMSE = 4.93;<br>AVD = 2.74
Peng et al. [100]	AttFocusNet:<br>- Based on the U-Net architecture.<br>- Integrating the AG into the encoder stage to suppress irrelevant features and focus on task-related ones during model learning.<br>- Building a focus structure which was adopted from YOLO-V5 to integrate image information into the channel space without initial pooling, aiming to preserve the integrity of ICH features, especially at small lesion points.	PRIVATE DATASET:<br>Patients from the hospital of Army Medical University (Southwest Hospital)	1027 scans	Dice = 0.908;<br>IoU = 0.874;<br>Sensitivity = 0.913;<br>PPV = 0.957;<br>HD95 = 5.960mm

Nijiati et al. [101]	Sym-TransNet:<br>- Based on the U-Net architecture.<br>- Connects encoding and decoding paths using symmetric Transformer.	PRIVATE DATASET:<br>Patients who were diagnosed with ICH from January 2019 to April 2022 at the First People's Hospital of Kashi Prefecture	1,157 patients	Dice:<br>ICH: 0.716;<br>IPH: 0.784;<br>IVH: 0.680;<br>EDH: 0.359;<br>SDH: 0.534;<br>SAH: 0.337;<br><br>Accuracy = 91.25%;<br>Sensitivity = 98.5%;<br>Specificity = 84.00%
Inkeaw et al. [102]	3D CNN based on the DeepMedic model architecture	PRIVATE DATASET:<br>Maharaj Nakorn Chiang Mai Hospital, Thailand, from January 1, 2014, to December 31, 2014.	153 patients	Dice = 0.59;<br>Sensitivity = 70%;<br>Specificity = 99%

Table S6. Summary of artificial intelligence (AI) models and performance metrics for the automated detection and quantification of midline shift in non-contrast head computed tomography (CT) scans of adult patients with traumatic brain injury (TBI), identified through a systematized review from database inception to 31 December 2025.

Approach	Study	Model	Dataset	Data Size	Performances
Landmark-based	Hooshmand et al. [103]	Ventricular geometric and anatomical information	PRIVATE DATASET: Internal dataset from the University of Michigan	170 patients	Sensitivity = 75%;<br>Specificity = 65%;<br>Precision = 53%;<br>Accuracy = 68%;<br>F1-Score = 0.62
	Jiang et al. [104]	Semi-automatic framework based on 3D Chan-Vese	N/A	N/A	N/A
DL-based	Wei et al. [105]	RLDN architecture with three phases: (1) multiscale line detection, (2) weighted line integration, and (3) regression-based line refinement	- Training & Testing: CQ500<br><br>- CLINICAL VALIDATION: PRIVATE DATASET - The Tongji Hospital Dataset	- Training: 100 scans - 500 slices<br>- Testing: 28 scans - 140 slices<br>- Clinical validation: 61 scans	**CQ500 test set:**<br>Mean line distance error = 1.17 ± 0.72 mm;<br>F1-score = 0.78<br><br>**Private dataset:**<br>Mean line distance error = 4.15 ± 3.97 mm;<br>F1-score = 0.61
	Yan et al. [106]	Modified Keypoint R-CNN	PRIVATE DATASET	300 scans - 7269 slices (split into 223 training, 31 validation, and 46 testing scans consisting of 5248, 769 and 1226 axial images)	MAE = 0.936;<br>Maximum sensitivity on testing set = 91.7%;<br>Maximum specificity on testing set = 97.4%;<br>Interclass correlation of 0.9899 with clinicians.
	Xia et al. [107]	CNNs framework with 3 stages: (1) U-Net for image alignment; (2) U-Net for midline segmentation; (3) Dynamic	- PRIVATE DATASET: Collected from Qilu Hospital of Shandong University (Qingdao) from January 2014 to April 2019.<br>- VALIDATION: dataset from 3 hospitals and CQ500	140 patients	Shift distance error = 1.14±0.91 mm;<br>Shift area error = 0.88±0.79 cm2; AUC-ROC of MLS-D = 0.799;<br>AUC-ROC of MLS-V = 0.831

		programming for pathfinding			
	Nag et al. [108]	2D U-Net	PRIVATE DATASET	45 patients	Accuracy = 94.05%;<br>Pixel error = 1.294 mm;<br>Area error = 66.4 mm2;<br>Volume error = 253.73 mm3
	Wu et al. [109]	3D U-Net architecture with three components: (1) a hemisphere-segmentation network, (2) rectification learning, and (3) midline correction	- PRIVATE DATASET<br>- EXTERNAL VALIDATION: CQ500 dataset	- Private dataset (519 patients, split 3:1:1)<br>- CQ500 dataset (491 patients)	**Private dataset:**<br>ASD = 0.96 ±0.5;<br>HD = 9.91±9.6<br><br>**CQ500 dataset**:<br>ASD = 0.84 ±0.4;<br>HD = 9.1±7.5
	Agrawal et al. [110]	3D CNN	PRIVATE DATASET: Collected from the Department of Neurosurgery, All India Institute of Medical Sciences, New Delhi from January 2020 to September 2020.	176 scans (train 156, test 20)	Accuracy = 55%;<br>Specificity = 70%;<br>Sensitivity = 40%;

Table S7. Summary of artificial intelligence (AI) models and performance metrics for intracranial pressure (ICP) estimation in non-contrast head computed tomography (CT) scans of adult patients with traumatic brain injury (TBI), identified through a systematized review from database inception to 31 December 2025.

Study	Methods	Dataset	Data Size	Performances
Pappu et al. [112]	- Develops a semiautomated image analysis algorithm using MATLAB to segment brain tissues from CSF in CT scans and calculate the ratio of CSF volume to the size of the intracranial vault (CSFV/ICVV).<br>- Plots the computed ratios against the recorded ICP values.<br>- Performs statistical analysis using the Statistics Toolbox in MATLAB.	PRIVATE DATASET: Collected from the University of New Mexico Hospital between January 2008 and December 2012.	20 patients - 45 scans	- A ratio CSFV/ICVV > .034 correlates with an ICP < 20 mmHg (p-value = 0.0046)<br><br>- A CSFV/ICVV threshold value of 0.034 was determined to have a predictive accuracy of nearly 67% for ICP
Shan et al. [113]	- Develops three models: the HU model; the MLS model; and the CE model.<br>- Uses these models to (1) predict increased ICP in a binary (yes/no) classification; and (2) classify ICP into three groups (≤22, 23–29, and ≥30 mmHg).	PRIVATE DATASET: Collected from the Department of Neurosurgery, Shanghai General Hospital from January 2018 to December 2019.	47 patients	**Binary classification:**<br>- HU Model:<br>Accuracy = 80.85%;<br>Precision = 83.33%;<br>Recall = 86.21%;<br>F1-score = 0.85<br><br>- MLS Model:<br>Accuracy = 61.7%;<br>Precision = 61.7%;<br>Recall = 100%;<br>F1-score = 0.76<br><br>- CE Model:<br>Accuracy = 63.83%;<br>Precision = 67.65%;<br>Recall = 79.31%;<br>F1-score = 0.78

				**Three-class classification:**<br>- HU Model:<br>Accuracy = 61.70%;<br>Precision = 61.06%;<br>Recall = 61.70%;<br>F1-score = 0.61<br><br>- MLS Model:<br>Accuracy = 40.43%;<br>Precision = 32.90%;<br>Recall = 40.43%;<br>F1-score = 0.29<br><br>- CE model: Failed according to the clinicians' expertise
Li et al. [114]	- Extracts 18 first-order and 40 second-order features from CT scans using PyRadiomics.<br>- Uses the LASSO method for feature filtration to select the most significant features.<br>- Develops three prediction models (CF, FO, SO) using multivariate logistic regression to predict intracranial hypertension (ICP >22 mmHg).	PRIVATE DATASET:<br>Collected from the department of neurosurgery, Shanghai General Hospital, from January 2020 to December 2021; and the department of neurosurgery of Qian nan People's Hospital from January 2021 to December 2021	- Shanghai: 50 patients<br>- Qian nan: 20 patients	- CF Model:<br>Accuracy = 64%;<br>Precision = 83.30%;<br>Recall = 65.79%;<br>F1-score = 0.74;<br>AUC-ROC = 0.65<br><br>- FO Model:<br>Accuracy = 74%;<br>Precision = 90%;<br>Recall = 72.97%;<br>F1-score = 0.81;<br>AUC-ROC = 0.76<br><br>- SO Model:<br>Accuracy = 80%;<br>Precision = 86.67%;<br>Recall = 81.25%;<br>F1-score = 0.83;

				AUC-ROC = 0.81

References

M. W. Dusenberry, C. K. Brown and K. L. Brewer, "Artificial neural networks: Predicting head CT findings in elderly patients presenting with minor head injury after a fall," *The American journal of emergency medicine,* vol. 35, no. 2, pp. 260-267, 2017.

C. Gan, D. Sun, K. Qin, H. Zhao and F. Xiao, "Improved traumatic brain injury classification approach based on deep learning," in *Proceedings of the 2020 9th International Conference on Bioinformatics and Biomedical Science*, 2020.

S. Zhang, T. Cao and H. Zhao, "Traumatic brain injury images classification method based on deep learning," in *Biometric Recognition: 15th Chinese Conference, CCBR 2021, Shanghai, China, September 10--12, 2021, Proceedings 15*, 2021.

H. Ellethy, V. Vegh and S. S. Chandra, "Interpretable 3D multi-modal residual convolutional neural network for mild traumatic brain injury diagnosis," in *Australasian Joint Conference on Artificial Intelligence*, 2023.

C. Sindhura, M. Al Fahim, P. K. Yalavarthy and S. Gorthi, "Fully automated sinogram-based deep learning model for detection and classification of intracranial hemorrhage," *Medical Physics,* vol. 51, no. 3, pp. 1944-1956, 2024.

J. Perez-Cano, Y. Wu, A. Schmidt, M. Lopez-Perez, P. Morales-Alvarez, R. Molina and A. K. Katsaggelos, "An end-to-end approach to combine attention feature extraction and Gaussian Process models for deep multiple instance learning in CT hemorrhage detection," *Expert Systems with Applications,* vol. 240, p. 122296, 2024.

N. K. Kar, S. Jana, A. Rahman, P. R. Ashokrao, G. Indhumathi and R. A. Mangai, "Automated Intracranial Hemorrhage Detection Using Deep Learning in Medical Image Analysis," in *2024 International Conference on Data Science and Network Security (ICDSNS)*, 2024.

J. Fan, X. Fan, C. Song, X. Wang, B. Feng, L. Li and G. Lu, "Dual-task vision transformer for rapid and accurate intracerebral hemorrhage CT image classification," *Scientific Reports,* vol. 14, no. 1, p. 28920, 2024.

Y. Wu, M. Iorga, S. Badhe, J. Zhang, D. R. Cantrell, E. J. Tanhehco, N. Szrama, A. M. Naidech, M. Drakopoulos, S. T. Hasan and others, "Precise Image-level Localization of Intracranial Hemorrhage on Head CT Scans with Deep Learning Models Trained on Studylevel Labels," *Radiology: Artificial Intelligence,* vol. 6, no. 6, p. e230296, 2024.

D. Agrawal, L. Poonamallee, S. Joshi and V. Bahel, "Automated intracranial hemorrhage detection in traumatic brain injury using 3D CNN," *Journal of Neurosciences in Rural Practice,* vol. 14, no. 4, p. 615, 2023.

M. Lopez-Perez, A. Schmidt, Y. Wu, R. Molina and A. K. Katsaggelos, "Deep Gaussian processes for multiple instance learning: Application to CT intracranial hemorrhage detection," *Computer Methods And Programs In Biomedicine,* vol. 219, p. 106783, 2022.

M. Ganeshkumar, V. Ravi, V. Sowmya, E. Gopalakrishnan, K. Soman and C. Chakraborty, "Identification of intracranial haemorrhage (ICH) using ResNet with data augmentation using CycleGAN and ICH segmentation using SegAN," *Multimedia Tools and Applications,* vol. 81, no. 25, pp. 36257-36273, 2022.

M. Altuve and A. Perez, "Intracerebral hemorrhage detection on computed tomography images using a residual neural network," *Physica Medica,* vol. 99, pp. 113-119, 2022.

P. Malik and A. Vidyarthi, "Stacked deep model-based classification of the multiclass brain hemorrhages in CT scans," *International Journal of Imaging Systems and Technology,* vol. 34, no. 1, p. e22955, 2024.

S. Gudadhe and A. Thakare, "A Novel Weighted Average 2D-CNN Ensemble for Intracranial Hemorrhage CT Image Classification Gudadhe," *International Journal of Engineering Trends and Technology,* vol. 72, no. 7, pp. 247-255, 2024.

T. H. Gencturk, F. K. Gulaglz and I. Kaya, "Detection and Segmentation of Subdural Hemorrhage on Head CT Images," *IEEE Access,* 2024.

M. N. Faiz, T. Badriyah and S. F. Kusuma, "Classification of Intracranial Hemorrhage Based on CT-Scan Image with Vision Transformer (ViT) Method," in *2024 International Electronics Symposium (IES)*, 2024.

S. Umapathy, M. Murugappan, D. Bharathi and M. Thakur, "Automated Computer-Aided Detection and Classification of Intracranial Hemorrhage Using Ensemble Deep Learning Techniques," *Diagnostics,* vol. 13, no. 18, p. 2987, 2024.

J. Sengupta, R. Alzbutas, P. Falkowski-Gilski and B. Falkowska-Gilska, "Intracranial hemorrhage detection in 3D computed tomography images using a bi-directional long short-term memory network-based modified genetic algorithm," *Frontiers in neuroscience,* vol. 17, p. 1200630, 2023.

M. Rajagopal, S. Buradagunta, M. Almeshari, Y. Alzamil, R. Ramalingam and V. Ravi, "An Efficient Framework to Detect Intracranial Hemorrhage Using Hybrid Deep Neural Networks," *Brain Sciences,* vol. 13, no. 3, p. 400, 2023.

O. Ozaltin, O. Coskun, O. Yeniay and A. Subasi, "Classification of brain hemorrhage computed tomography images using OzNet hybrid algorithm," *International Journal of Imaging Systems and Technology,* vol. 33, no. 1, pp. 69-91, 2023.

N. Negm, G. Aldehim, F. M. Nafie, R. Marzouk, M. Assiri, M. I. Alsaid, S. Drar and S. Abdelbagi, "Intracranial Haemorrhage Diagnosis Using Willow Catkin Optimization With Voting Ensemble Deep Learning on CT Brain Imaging," *IEEE Access,* vol. 11, pp. 75474-75483, 2023.

P. Liu, G. Ning, L. Shi, Q. Zhou and X. Chen, "Fine-grained classification of intracranial haemorrhage subtypes in head CT scans," *IET Computer Vision,* vol. 17, no. 2, pp. 170-188, 2023.

M. Asif, M. A. Shah, H. A. Khattak, S. Mussadiq, E. Ahmed, E. A. Nasr and H. T. Rauf, "Intracranial Hemorrhage Detection Using Parallel Deep Convolutional Models and Boosting Mechanism," *Diagnostics,* vol. 13, no. 4, p. 652, 2023.

M. N. Akram, M. U. Yaseen, M. Waqar, M. Imran and A. Hussain, "A Double-Branch Xception Architecture for Acute Hemorrhage Detection and Subtype Classification," *Computers, Materials & Continua,* vol. 76, no. 3, 2023.

Q. Zhou, W. Zhu, F. Li, M. Yuan, L. Zheng and X. Liu, "Transfer Learning of the ResNet-18 and DenseNet-121 Model Used to Diagnose Intracranial Hemorrhage in CT Scanning," *Current Pharmaceutical Design,* vol. 28, no. 4, pp. 287-295, 2022.

J. Sengupta and R. Alzbutas, "Intracranial Hemorrhages Segmentation and Features Selection Applying Cuckoo Search Algorithm with Gated Recurrent Unit," *Applied Sciences,* vol. 12, no. 21, p. 10851, 2022.

A. Phaphuangwittayakul, Y. Guo, F. Ying, A. Y. Dawod, S. Angkurawaranon and C. Angkurawaranon, "An optimal deep learning framework for multi-type hemorrhagic lesions detection and quantification in head CT images for traumatic brain injury," *Applied Intelligence,* pp. 1-19, 2022.

B. M. Monica Jenefer, K. Senathipathi, Aarthi and Annapandi, "Detection and categorization of acute intracranial hemorrhage subtypes using a multilayer DenseNet-ResNet architecture with improved random forest classifier," *Concurrency and Computation: Practice and Experience,* vol. 34, no. 22, p. e7167, 2022.

S. Korra, R. Mamidi, N. R. Soora, K. V. Kumar and N. C. S. Kumar, "Intracranial hemorrhage subtype classification using learned fully connected separable convolutional network," *Concurrency and Computation: Practice and Experience,* vol. 34, no. 24, p. Concurrency and Computation: Practice and Experience, 2022.

A. Hussain, M. U. Yaseen, M. Imran, M. Waqar, A. Akhunzada, M. Al-Ja'afreh and A. El Saddik, "An Attention-Based ResNet Architecture for Acute Hemorrhage Detection and Classification: Toward a Health 4.0 Digital Twin Study," *IEEE Access,* vol. 10, pp. 126712-126727, 2022.

O. F. Ertugrul and M. F. Akil, "Detecting hemorrhage types and bounding box of hemorrhage by deep learning," *Biomedical Signal Processing and Control,* vol. 71, p. 103085, 2022.

D. Alis, C. Alis, M. Yergin, C. Topel, O. Asmakutlu, O. Bagcilar, Y. D. Senli, A. Ustundag, V. Salt, S. N. Dogan and others, "A joint convolutional-recurrent neural network with an attention mechanism for detecting intracranial hemorrhage on noncontrast head CT," *Scientific Reports,* vol. 12, no. 1, p. 2084, 2022.

L. P. Kothala and S. R. Guntur, "TL-LFF Net: transfer learning based lighter, faster, and frozen network for the detection of multi-scale mixed intracranial hemorrhages through genetic optimization algorithm," *International Journal of Machine Learning and Cybernetics,* pp. 1-27, 2024.

B. He, Z. Xu, D. Zhou and L. Zhang, "Deep multiscale convolutional feature learning for intracranial hemorrhage classification and weakly supervised localization," *Heliyon,* vol. 10, no. 9, 2024.

V. Vidhya, U. Raghavendra, A. Gudigar, S. Basak, S. Mallappa, A. Hegde, G. R. Menon, P. D. Barua, M. Salvi, E. J. Ciaccio and others, "YOLOv5s-CAM: A Deep Learning Model for Automated Detection and Classification for Types of Intracranial Hematoma in CT Images," *IEEE Access,* vol. 11, pp. 141309-141328, 2023.

L. P. Kothala, P. Jonnala and S. R. Guntur, "Localization of mixed intracranial hemorrhages by using a ghost convolution-based YOLO network," *Biomedical Signal Processing and Control,* vol. 80, p. 104378, 2023.

J. Cheng, K. Gao and L. Zhou, "Study of intracranial haematoma localisation based on improved RetinaNet," *Proceedings of the 2023 8th International Conference on Multimedia and Image Processing,* pp. 73-78, 2023.

Y. Zhang, Y. Huang and K. Hu, "Multi-scale object equalization learning network for intracerebral hemorrhage region segmentation," *Neural Networks,* vol. 179, p. 106507, 2024.

L. Zhang, D. Song, H. Qiu, L. Ye and Z. Xu, "Fuzzy C-means clustering algorithm applied in computed tomography images of patients with intracranial hemorrhage," *Frontiers in Neuroinformatics,* vol. 18, p. 1440304, 2024.

D. Zhang, Z. Ma, R. Gong, L. Lian, Y. Li, Z. He, Y. Han, J. Hui, J. Huang, J. Jiang and others, "Using Natural Language Processing (GPT-4) for Computed Tomography Image Analysis of Cerebral Hemorrhages in Radiology: Retrospective Analysis," *Journal of medical Internet research,* vol. 26, p. e58741, 2024.

W. Xu, Z. Sha, T. Tan, W. Liu, Y. Chen, Z. Li, X. Pan, R. Jiang and H. Yang, "Automatic Segmentation of Intracranial Hemorrhage in Computed Tomography Scans with Convolution Neural Networks," *Journal of Medical and Biological Engineering,* vol. 44, no. 4, pp. 575-581, 2024.

Y. Xiao, Y. Hou, Z. Wang, Y. Zhang, X. Li, K. Hu and X. Gao, "Multi-scale Perception and Feature Refinement Network for multi-class segmentation of intracerebral hemorrhage in CT images," *Biomedical Signal Processing and Control,* vol. 88, p. 105614, 2024.

H. Xiao, X. Shi, Q. Xia, L. Chen, D. Chen, Y. Li, L. Li, Q. Liu and H. Zhao, "DFMA-ICH: a deformable mixed-attention model for intracranial hemorrhage lesion segmentation based on deep supervision," *Neural Computing and Applications,* vol. 36, no. 15, pp. 8657-8679, 2024.

A. Nafees and P. Prakasam, "Spatial attention-based CSR-Unet framework for subdural and epidural hemorrhage segmentation and classification using CT images," *BMC Medical Imaging,* vol. 24, p. 285, 2024.

J. C. Rajapakse, C. H. How, Y. H. Chan, L. C. P. Hao, A. Padhi, V. Adrakatti, I. Khan and T. Lim, "Two-Stage Approach to Intracranial Hemorrhage Segmentation From Head CT Images," *IEEE Access,* 2024.

H. Liu, Y. Zeng, H. Li, F. Wang, J. Chang, H. Guo and J. Zhang, "DDANet: A deep dilated attention network for intracerebral haemorrhage segmentation," *IET Systems Biology,* vol. 18, no. 6, pp. 285-297, 2024.

E. Lin and E. L. Yuh, "Semi-supervised Learning for Generalizable Intracranial Hemorrhage Detection and Segmentation," *Radiology: Artificial Intelligence,* vol. 6, no. 3, p. e230077, 2024.

G. Li, K. Gao, C. Liu and S. Li, "Intracranial hematoma segmentation on head CT based on multiscale convolutional neural network and transformer," *IET Image Processing,* vol. 18, no. 12, pp. 3480-3495, 2024.

H. Wang and X. Wang, "MSRL-Net: An Automatic Segmentation of Intracranial Hemorrhage for CT Images Based on the U-Net Framework," *Applied Sciences,* vol. 13, no. 21, p. 11781, 2023.

Q. Liu, B. J. MacIntosh, T. Schellhorn, K. Skogen, K. E. Emblem and A. Bjornerud, "Voxels Intersecting Along Orthogonal Levels Attention U-Net for Intracerebral Haemorrhage Segmentation in Head CT," in *2023 IEEE 20th International Symposium on Biomedical Imaging (ISBI)*, 2023.

M. M. Khan, M. E. Chowdhury, A. S. Arefin, K. K. Podder, M. S. A. Hossain, A. Alqahtani, M. Murugappan, A. Khandakar, A. Mushtak and M. Nahiduzzaman, "A Deep Learning-Based Automatic Segmentation and 3D Visualization Technique for Intracranial Hemorrhage Detection Using Computed Tomography Images," *Diagnostics,* vol. 13, no. 15, p. 2537, 2023.

M. F. Sharrock, W. A. Mould, M. Hildreth, E. P. Ryu, N. Walborn, I. A. Awad, D. F. Hanley and J. Muschelli, "Bayesian deep learning outperforms clinical trial estimators of intracerebral and intraventricular hemorrhage volume," *Journal of Neuroimaging,* vol. 32, no. 5, pp. 968-976, 2022.

Q. Peng, X. Chen, C. Zhang, W. Li, J. Liu, T. Shi, Y. Wu, H. Feng, Y. Nian and R. Hu, "Deep learning-based computed tomography image segmentation and volume measurement of intracerebral hemorrhage," *Frontiers in Neuroscience,* vol. 16, p. 965680, 2022.

M. Nijiati, A. Tuersun, Y. Zhang, Q. Yuan, P. Gong, A. Abulizi, A. Tuoheti, A. Abulaiti and X. Zou, "A symmetric prior knowledge based deep learning model for intracerebral hemorrhage lesion segmentation," *Frontiers in Physiology,* vol. 13, p. 977427, 2022.

P. Inkeaw, S. Angkurawaranon, P. Khumrin, N. Inmutto, P. Traisathit, J. Chaijaruwanich, C. Angkurawaranon and I. Chitapanarux, "Automatic hemorrhage segmentation on head CT scan for traumatic brain injury using 3D deep learning model," *Computers in Biology and Medicine,* vol. 146, p. 105530, 2022.

M. Hooshmand, S. R. Soroushmehr, C. Williamson, J. Gryak and K. Najarian, "Automatic midline shift detection in traumatic brain injury," in *2018 40th Annual International Conference of the IEEE Engineering in Medicine and Biology Society (EMBC)*, 2018.

C. Jiang, J. Cao, C. Williamson, N. Farzaneh, V. Rajajee, J. Gryak, K. Najarian and S. R. Soroushmehr, "Midline Shift vs. Mid-Surface Shift: Correlation with Outcome of Traumatic Brain Injuries," in *2019 IEEE International Conference on Bioinformatics and Biomedicine (BIBM)*, 2019.

H. Wei, X. Tang, M. Zhang, Q. Li, X. Xing, X. Sean Zhou, Z. Xue, W. Zhu, Z. Chen and F. Shi, "The delineation of largely deformed brain midline using regression-based line detection network," *Medical Physics,* vol. 47, no. 11, pp. 5531-5542, 2020.

J.-L. Yan, Y.-L. Chen, M.-Y. Chen, B.-A. Chen, J.-X. Chang, C.-C. Kao, M.-C. Hsieh, Y.-T. Peng, K.-C. Huang and P.-Y. Chen, "A robust, fully automatic detection method and calculation technique of midline shift in intracranial hemorrhage and its clinical application," *Diagnostics,* vol. 12, no. 3, p. 693, 2022.

X. Xia, X. Zhang, Z. Huang, Q. Ren, H. Li, Y. Li, K. Liang, H. Wang, K. Han and X. Meng, "Automated detection of 3D midline shift in spontaneous supratentorial intracerebral haemorrhage with non-contrast computed tomography using deep convolutional neural networks," *American Journal of Translational Research,* vol. 13, no. 10, p. 11513, 2021.

M. K. Nag, A. Gupta, A. Hariharasudhan, A. K. Sadhu, A. Das and N. Ghosh, "Quantitative analysis of brain herniation from non-contrast CT images using deep learning," *Journal of Neuroscience Methods,* vol. 349, p. 109033, 2021.

D. Wu, H. Li, J. Chang, C. Qin, Y. Chen, Y. Liu, Q. Zhang, B. Huang, M. Feng, R. Wang and others, "Automatic brain midline surface delineation on 3d ct images with intracranial hemorrhage," *IEEE Transactions on Medical Imaging,* vol. 41, no. 9, pp. 2217-2227, 2022.

D. Agrawal, S. Joshi, V. Bahel, L. Poonamallee and A. Agrawal, "Three dimensional convolutional neural network-based automated detection of midline shift in traumatic brain injury cases from head computed tomography scans," *Journal of Neurosciences in Rural Practice,* vol. 15, no. 2, p. 293, 2024.

S. Pappu, J. Lerma and T. Khraishi, "Brain CT to assess intracranial pressure in patients with traumatic brain injury," *Journal of Neuroimaging,* vol. 26, no. 1, pp. 37-40, 2016.

Y. Shan, Y. Li, X. Xu, J. Feng, X. Wu and G. Gao, "Evaluation of intracranial hypertension in traumatic brain injury patient: a noninvasive approach based on cranial computed tomography features," *Journal of Clinical Medicine,* vol. 10, no. 11, p. 2524, 2021.

Y. Li, G. Zhang, Y. Shan, X. Wu, J. Liu, Y. Xue and G. Gao, "Non-invasive assessment of intracranial hypertension in patients with traumatic brain injury using computed tomography radiomic features: a pilot study," *Journal of Neurotrauma,* vol. 40, no. 3-4, pp. 250-259, 2023.
