# Supplementary material for: AI-Based Approaches for the Identification and Quantification of Traumatic Brain Injury in Computed Tomography Imaging: Systematic Review
Source: JMIR AI. 2026 Sep 10;5:e87794. doi: 10.2196/87794 (PMC13561197; doi:10.2196/87794)
Supplement: Supplementary file 4 [file ai-v5-e87794-s004.pdf]

Title	Year	Author	URL	Main Ideas	Dataset Details	Dataset Size	Data Preprocessing	Methods / Models	Metrics	Performance
Three dimensional convolutional neural network-based automated detection of midline shift in traumatic brain injury cases from head computed tomography scans	2024	Agrawal et al.	https://ruralneuropractice.com/view-pdf/?article=b95058171c57f9e18b3296c7d201b1d6MXV4t/pzDLDveA==	Using CNNs to classify CT scans into Normal (Non-MLS) or MLS	**PRIVATE DATASET**<br><br>The study enrolled TBI cases, who presented at the Department of Neurosurgery, All India Institute of Medical Sciences, New Delhi. Institutional ethics committee permission was taken before starting the study. The data collection was carried out for over nine months, i.e., from January 2020 to September 2020. The data collection included head CT scans, patient demographics, clinical details as well as radiologist's reports.<br><br>Slice thickness of 1 mm and 30s kernel<br><br>**LABELING:** The binary labels for each imaging study, indicating negative or positive MLS, were derived from the clinical radiology reports	176 scans (train 156, test 20)	1. Resize (128x128x64)<br>2. Min-max normalization<br>3. Window level (-300), window width (1400)<br>4. Contrast enhancement<br>5. Gaussian smoothing<br>6. Rotations	3D CNNs to classify CT scans into Normal (Non-MLS) or MLS	Accuracy, Specificity, Sensitivity	Accuracy 55%, Specificity 70%, Sensitivity 40%
Automatic Brain Midline Surface Delineation on 3D CT Images With Intracranial Hemorrhage	2022	Wu et al.	https://ieeexplore-ieee-org.ezproxy-f.deakin.edu.au/document/9737524	Instead of segmenting midline directly, this paper proposes a novel and efficient hemisphere-segmentation framework (HSF) based on 3D-Unet architecture for 3D brain midline surface delineation using 3D CT images. The network can be divided into three components: (1) hemisphere-segmentation network, (2) rectification learning, (3) midline correction	**PRIVATE DATASET:**<br>In-house, no more information<br><br>**EXTERNAL VALIDATION:** CQ500 dataset	Private dataset (519 patients, split 3:1:1)<br>CQ500 dataset (491 patients)	1. Resample the voxel spacing (1.0 x 1.0 x 1.0 mm3)<br>2. Center crop, reisze (192x192x160)<br>3. Window level (brain 0->80, bone -800->2000, subdural -20->180)	Hemisphere-segmentation framework (HSF) based on 3D-Unet architecture. The network can e divided into three components: (1) hemisphere-segmentation network, (2) rectification learning, (3) midline correction	Average Surface Distance (ASD-mm) and Hausdorff Distance (HD-mm)	Private: ASD 0.96±0.5; HD 9.91±9.6<br>CQ500: ASD 0.84±0.4; HD 9.1±7.5
Automated detection of 3D midline shift in spontaneous supratentorial intracerebral haemorrhage with non-contrast computed tomography using deep convolutional neural networks	2021	Xia et al.	https://pmc.ncbi.nlm.nih.gov/articles/PMC8581948/	Proposed a 3-phase DL-based convolutional neural networks framework to non-contrast CT scans for automated 2D/3D brain midline shift measurement	**PRIVATE DATASET:**<br>Patients with a primary diagnosis of supratentorial ICH, admitted to the acute care stroke unit of Qilu Hospital of Shandong University (Qingdao) from January 2014 to April 2019. Patients were grouped by the degree of midline shifting into no-shifting and midline-shifting groups.<br><br>CT scans were performed using Siemens (SOMATOM Definition FLASH, Siemens Healthcare) and Philips (Brilliance 64, Philips Medical Systems) scanners<br><br>**VALIDATION:** dataset from 3 hospitals and CQ500	140 patients	N/A	CNNs framework with 3 stages: (1) U-Net for image alignment; (2) U-Net for midline segmentation; (3) Dynamic programming for pathfinding.<br><br>They estimated MLS by calculating the distance of midline shift (MLS-D) and volume of midline shift (MLS-V). The horizontal distances had been calculated between the actual midline and the ideal midline on all shifted slices and the largest one chosen as MLS-D. The MLS-V was calculated as the number of voxels between the actual and ideal midline multi-plied by the physical size of a voxel. The voxel was defined as a discrete element of volume (x-spacing × y-spacing × z-spacing).	Shift Distance Error<br>Shift Area Error<br><br>Area Under the Receiver Operating Characteristic curve (AUC)	Shift distance error 1.14±0.91 mm<br>Shift area error 0.88±0.79 cm2<br><br>AUC of MLS-D: 0.799<br>AUC of MLS-V: 0.831
Midline Shift vs. Mid-Surface Shift: Correlation with Outcome of Traumatic Brain Injuries	2021	Jiang et al.	https://pmc-ncbi-nlm-nih-gov.ezproxy-f.deakin.edu.au/articles/PMC7871460/	A novel measurement of overall mid-surface shift is proposed that quantifies the total volume of brain tissue shifted across the midline	N/A	N/A	N/A	Using 3D Chan-Vese segmentation combining with active contour on contrast-adjusted CT images to segment the brain mask. The mid-surface was then annotated manually using biomarkers in the brain. To calculate the volume of shift, the ideal midline is first identified as the line formed by the intersections of the annotated mid surface curve and the inner edge of the skull. Finally, the ratio between the volume of shift and brain volume is calculated.	N/A	N/A
Automatic Midline Shift Detection in Traumatic Brain Injury	2018	Hooshmand et al.	https://par.nsf.gov/servlets/purl/10298281	Using the ventricular geometric patterns to calculate the actual midline and also anatomical information to detect the ideal midline. The distance between these two lines is used as an estimate of MLS.	**PRIVATE DATASET:**<br>Internal dataset from the University of Michigan	170 patients	N/A	Using the ventricular geometric patterns to calculate the actual midline and also anatomical information to detect the ideal midline. The distance between these two lines is used as an estimate of MLS.	Sensitivity; Specificity; Precision; Accuracy; F1-Score	Sensitivity = 0.75; Specificity = 0.65; Precision = 0.53; Accuracy = 0.68; F1-Score = 0.62
Quantitative analysis of brain herniation from non-contrast CT images using deep learning	2021	Nag et al.	https://www-sciencedirect-com.ezproxy-b.deakin.edu.au/science/article/pii/S0165027020304568?via%3Dihub	Ideal midline (iML) divides the healthy brain into two (right and left) nearly equal hemispheres. In this study, a convolutional neural network (CNN) was used to predict the deformed left and right hemispheres.	**PRIVATE DATASET:**<br>Non-contrast CT. CT data were collected from n = 80 patients visiting one collabo rating neuroimaging center (EKO diagnostics, Medical College and Hospitals campus, Kolkata, India) with different types of brain hemorrhages. The slice thickness was 1.25 mm, rescalable slope of 1, and rescalable intercept at -1024. The window width and height were 100 Hounsfield Unit (HU) and 40 HU respectively. The collected samples were converted into neuroimaging informatics technology initiatives (NifTI format. The size of each CT human brain was 512 × 512 × 96, where 512 is the spatial resolution (pixels in length and width and 96 was the number of slices. The original dML was derived from the masks drawn by an expert radiologist (Dr. Anup Kumar Sadhu, MD; coauthor of this paper with more than 16 years of experience.	45 patients	N/A	Using 2D U-Net model to segment the hemispheres and based on that, quantify the iML and dML.	Accuracy, pixel error, area error, volume error	U-Net achieved 94.05% validation accuracy; Pixel error = 1.294 mm; Area error = 66.4 mm2; Volume error = 253.73 mm3
The delineation of largely deformed brain midline using regression-based line detection network	2020	Wei et al.	https://aapm-onlinelibrary-wiley-com.ezproxy-b.deakin.edu.au/doi/10.1002/mp.14302	They propose a novel regression-based line detection network (RLDN) for the robust mid line delineation, especially in largely deformed brains. Specifically, to improve the robustness of delineation in largely deformed brains, they regard the delineation of the midline as the skeleton extraction task and then use the multiscale bidirectional integration module to acquire more represen tative features. Based on the skeleton extraction, they incorporate the regression task into it to delineate more accurate and continuous midline, especially in largely deformed brains.	CQ500<br><br>**CLINICAL VALIDATION**: **PRIVATE DATASET -** The Tongji Hospital Dataset	training: 100 scans - 500 slices<br>testing: 28 scans - 140 slices<br><br>Clinical validation: 61 scans	1. Resample to uniform resolution (0.5 x 0.5 mm2)<br>2. Intensity correction<br>3. Crop (400x288, patch containing only the brain region)<br>4. Augmentation: random rotating, random flipping, random brightness changing	Uses a self-built RLDN, comprising three phases: multiscale line detection, weighted line integration, and regression-based line refinement.	Mean line distance error; F1-score; and some other distance errors	Mean line distance error and F1-score were 1.17 ± 0.72 mm and 0.78 on CQ 500 test set, and 4.15 ± 3.97 mm and 0.61 on the private dataset
A Robust, Fully Automatic Detection Method and Calculation Technique of Midline Shift in Intracranial Hemorrhage and Its Clinical Application	2022	Yan et al.	https://pmc-ncbi-nlm-nih-gov.ezproxy-b.deakin.edu.au/articles/PMC8947005/	The automatic detection of MLS was based on modified Keypoint R-CNN with keypoint detection followed by training on the ResNet-FPN-50 backbone.	**PRIVATE DATASET:**<br>We retrospectively included 300 consecutively collected non-contrast brain CT scans from July 2019 to January 2020 from the same single CT machine (GE LightSpeed, slice thickness = 0.625 mm) in the emergent department in our institution.	300 scans - 7269 slices (split into 223 training, 31 validation, and 46 testing scans consisting of 5248, 769 and 1226 axial images)	1. Resize the slice to 512 x 512<br>2. Create 3-channel image by stacking 3 spatially adjacent slices<br>3. Window level and width: (80, 200), (40, 380), (600, 2800)<br>4, Normalization to (0, 1)<br>5. Augmentation: random flipping, random affine, random erasing, metal artifact simulation	Uses a modified Keypoint R-CNN with keypoint detection, trained on ResNet-FPN-50 backbone to detect 4 keypoints: (1) the anterior end of the falx, (2) the posterior end of the falx, (3) the anterior end of the septum pellucidum near anterior commissure, (4) and the posterior end of the septum pellucidum. The MLS is then calculated by the distance between the middle point of the connection of the anterior and posterior falx and the midpoint of the septum pellucidum.	Mean Square Error (MAE); Interclass correlation; Sensitivity; Specificity	MAE = 0.936; Maximum sensitivity on testing set = 91.7%; Maximum specificity on testing set = 97.4%; Interclass correlation of 0.9899 with clinicians.

Title	Year	Author	URL	Main Ideas	Dataset Details	Dataset Size	Data Preprocessing	Methods / Models	Metrics	Performance
Brain CT to assess intracranial pressure in patients with traumatic brain injury	2016	Pappu et al.	https://onlinelibrary-wiley-com.ezproxy-b.deakin.edu.au/doi/full/10.1111/jon.12289	Morphologic features of computed tomography (CT) scans of the brain can be used to estimate intracranial pressure (ICP) via an image-processing algorithm. A measure is proposed that is a function of the distribution of cerebrospinal fluid (CSF) in and around the brain.	**PRIVATE DATASET:**<br><br>Patients with severe traumatic brain injury who were admitted to the Level 1 trauma center of the University of New Mexico Hospital between January 2008 and December 2012, had ICP monitors placed and also had a noncontrast CT done within 2 hours of the placement of the monitor. The corresponding recorded ICP's were also obtained from the electronic medical record.	20 patients - 45 scans	N/A	A semiautomated image analysis algorithm was developed using MATLAB to segment brain parenchyma from CSF in CT scans. Each pixel is assigned an indicator value for CSF, blood, or parenchyma. The algorithm calculates the ratio of CSF volume to the size of the intracranial vault (csfv/icvv). The computed csfv/icvv ratios were plotted against the recorded ICP values. Statistical analysis was performed using the Statistics Toolbox from MATLAB.	Correlation Coefficient; Receiver Operating Characteristic (ROC) Curve; Accuracy	A ratio csfv/icvv > .034 correlates with an ICP < 20 mmHg (P = .0046)<br><br>A csfv/icvv threshold value of 0.034 was determined to have a predictive accuracy of nearly 67% for ICP
Evaluation of Intracranial Hypertension in Traumatic Brain Injury Patient: A Noninvasive Approach Based on Cranial Computed Tomography Features.	2021	Shan et al.	https://pmc-ncbi-nlm-nih-gov.ezproxy-b.deakin.edu.au/articles/PMC8200948/	Assessing intracranial pressure (ICP) levels in patients with traumatic brain injury (TBI) through investigating the Hounsfield unit (HU) features of computed tomography (CT) images	**PRIVATE DATASET:**<br><br>Clinical data of patients with TBI admitted to the Department of Neurosurgery, Shanghai General Hospital from January 2018 to December 2019	47 patients	N/A	Introduces three models: Hounsfield unit (HU) model using HU-related features as the input, MLS model using MLS data as the input, and clinical expertise (CE) model. The three models were used to predict increased ICP (binary yes/no classification), as well as classify ICP into three groups (≤22, 23–29, and ≥30 mmHg), and their respective performance was compared.	Accuracy; Precision; Recall; F1-score	**Binary classification:**<br>HU Model: Accuracy = 80.85%; Precision = 83.33%; Recall = 86.21%; F1-score = 0.85<br><br>MLS Model: Accuracy = 61.7%; Precision = 61.7%; Recall = 100%; F1-score = 0.76<br><br>CE Model: Accuracy = 63.83%; Precision = 67.65%; Recall = 79.31%; F1-score = 0.78<br><br>**Three-class classification:**<br>HU Model: Accuracy = 61.70%; Precision = 61.06%; Recall = 61.70%; F1-score = 0.61<br><br>MLS Model: Accuracy = 40.43%; Precision = 32.90%; Recall = 40.43%; F1-score = 0.29<br><br>**CE model:** Failed according to the clinicians' expertise
Noninvasive assessment of intracranial hypertension in patients with traumatic brain injury using CT radiomic features：a pilot study.	2023	Li et al.	https://www-liebertpub-com.ezproxy-b.deakin.edu.au/doi/epub/10.1089/neu.2022.0277		**PRIVATE DATASET:**<br>Patients with TBI from the department of neurosurgery, Shanghai General Hospital, from January 2020 to December 2021;<br><br>Patients with TI from the department of neurosurgery of Qian nan People's Hospital from January 2021 to December 202	50 patients<br><br>20 patients	N/A	Radiomic features, including 18 first-order and 40 second-order features, are extracted from CT images using PyRadiomics. First-order features describe the gray-level frequency distribution based on pixel intensity histogram. Second-order features describe the spatial distribution of voxel intensity levels based on co-occurrence matrixes such as gray-level co-occurrence matrix (GLCM) and gray-level run-length matrix (GLRLM). The least absolute shrinkage and selection operator (LASSO) method is used for feature filtration to select the most significant features. Three prediction models are developed using multivariate logistic regression to predict the intracranial hypertension (ICP >22mgHg): Clinical features (CF) model, First-order features (FO) model, Second-order features (SO) model	Accuracy; Precision; Recall; F1-score; AUC	CF Model: Accuracy = 64%; Precision = 83.30%; Recall = 65.79%%; F1-score = 0.74; AUC = 0.65<br><br>FO Model: Accuracy = 74%; Precision = 90%; Recall = 72.97%; F1-score = 0.81; AUC = 0.76<br><br>SO Model: Accuracy = 80%; Precision = 86.67%; Recall = 81.25%; F1-score = 0.83; AUC = 0.81

Title	Year	Author	URL	Main Ideas	Dataset Details	Dataset Size	Data Preprocessing	Methods / Models	Metrics	Performance
Artificial neural networks: Predicting head CT findings in elderly patients presenting with minor head injury after a fall	2017	Dusenberry et al.	https://linkinghub-elsevier-com.ezproxy-b.deakin.edu.au/retrieve/pii/S0735-6757(16)30780-X	To construct an artificial neural network (ANN) model that can predict the presence of acute CT findings with both high sensitivity and high specificity when applied to the population of patients ≥ age 65 years who have incurred minor head injury after a fall.	**PRIVATE DATASET:**<br><br>Patients ≥ 65 years old presenting or transferring to the ED of a Level 1 Trauma Center teaching hospital with minor head injury after a fall between January 1st 2008 and September 30th 2013.	514 patients	N/A	Builds an ANN model to classify the CT scans as acute CT findings presence or not.	Accuracy; Precision; Recall; Specificity; Negative Predictive Value (NPV)	Accuracy = 93.14%; Precision = 88.00%; Recall = 97.78%; Specificity = 89.47%; NPV = 98.08%
Improved Traumatic Brain Injury Classification Approach Based on Deep Learning	2020	Gan et al.	https://dl-acm-org.ezproxy-b.deakin.edu.au/doi/fullHtml/10.1145/3431943.3432288	Propose a novel imaging diagnosis model of TBI based on convolutional neural network (CNN) combined with recurrent neural network (RNN) and embedded squeeze-and-excitation (SE) module	**PRIVATE DATASET**	226 patients<br>Normal: 306 slices<br>TBI: 525 slices	1. Crop to 224x224<br>2. Data augmentation	Develops an architecture based on ResNet with embedded squeeze-and-excitation (SE) module and LSTM, then uses transfer learning with ImageNet pretrained weights to fine-tune the model.	Accuracy; Sensitivity; Specificity	Accuracy = 95.9%; Sensitivity = 93.3%; Specificity = 98.9%
Interpretable 3D Multi-modal Residual Convolutional Neural Network for Mild Traumatic Brain Injury Diagnosis	2024	Ellethy et al.	https://link.springer.com/chapter/10.1007/978-981-99-8388-9_39	Introduce an interpretable 3D Multi-Modal Residual Convolutional Neural Network (MRCNN) for mTBI diagnostic model enhanced with Occlusion Sensitivity Maps (OSM)	TRACK-TBI	296 patients	1. Convert CT images to NIfTI format<br>2. Window width 80, window level 40<br>3. Resampling to 128x128x64<br>4. Remove background noises	Builds a multi-modal architecture based on RCNN model, with an additional branch for clinical data and an infusion layer to merge the CT features with the clinical features.	Accuracy; Sensitivity; Specificity	Accuracy = 82.4% ± 1.7%; Sensitivity = 82.6% ± 5.1%; Specificity = 81.6% ± 5.4%
Traumatic Brain Injury Images Classification Method Based on Deep Learning	2021	Zhang et al.	https://link.springer.com/chapter/10.1007/978-3-030-86608-2_18	Propose a new CNN architecture to classify CT images of TBI and determine whether there is damage.	**PRIVATE DATASET**	636 images	1. Crop to 224x224<br>2. Data augmentation	Proposes VGG-SE-PCR, which is composed of the traditional convolutional neural network VGG-S, the squeeze and excitation module SE and pixel-wise correlation retaining module PCR, to classify CT images of TBI and determine whether there is damage.	Accuracy; Sensitivity; Specificity	Accuracy = 89.3%; Sensitivity = 98.6%; Specificity = 82.9%

Title	Year	Author	URL	Main Ideas	Dataset Details	Dataset Size	Data Preprocessing	Methods / Models	Metrics	Performance
Fully automated sinogram-based deep learning model for detection and classification of intracranial hemorrhage	2024	Sindhura et al.	https://aapm-onlinelibrary-wiley-com.ezproxy-f.deakin.edu.au/doi/full/10.1002/mp.16714	Introduce a fully automated deep learning framework that can directly detect and classify Intracranial Hemorrhages (ICH) from sinograms	**RSNA**	361,838 slices	N/A	The proposed method consists of a two-stage end-to-end deep learning framework. The first stage of the framework is Intensity Transformed Sinogram Sythesizer (ITSS), which synthesizes sinograms that are equivalent to the intensity-transformed CT images. The second stage comprises of a cascaded Convolutional Neural Network-Recurrent Neural Network (CNN-RNN) model that detects and classifies hemorrhages from the synthesized sinograms. The CNN module extracts high-level features from each input sinogram, while the RNN module provides spatial correlation of the neighborhood regions in the sinograms.	Accuracy; Sensitivity; Specificity	Accuracy = 95.50%; Sensitivity = 93.16%; Specificity = 97.10%
An end-to-end approach to combine attention feature extraction and Gaussian Process models for deep multiple instance learning in CT hemorrhage detection	2024	Pérez-Cano et al.	https://www-sciencedirect-com.ezproxy-f.deakin.edu.au/science/article/pii/S0957417423027987	End-to-end multiple instance learning (MIL) method for hemorrhage detection.	**RSNA (training)**<br><br>**CQ500 (testing)**	674,258 images from 19,530 studies<br><br>491 patients	N/A	Proposes two main end-to-end MIL models:<br>1. E2E-Att-GP: This architecture first processes each CT slice through a CNN for feature extraction. The resulting feature vectors are then fed into an attention mechanism that calculates weights for each slice. A weighted sum of these feature vectors is computed, representing the aggregated bag-level features. This aggregated feature vector then serves as input to a Sparse Gaussian Process (SGP) for probabilistic classification, followed by a final fully connected layer.<br>2. In this variant, the CNN extracts features from each slice, which are then directly passed through an SGP layer, yielding stochastic features at the instance level. These stochastic features are then fed into an attention mechanism to obtain a weighted sum. Finally, a fully connected layer with sigmoid activation performs the bag-level classification.	Accuracy; F1-Score; Precision; Recall; ROC-AUC; Cohen's kappa	**RSNA:**<br>**E2E-Att-GP**<br>Accuracy = 0.876 ± 0.023 ; F1-Score = 0.886 ± 0.011; Precision = 0.825 ± 0.032; Recall = 0.959 ± 0.025; ROC-AUC = 0.965 ± 0.007<br>**E2E-GP-Att**<br>Accuracy = 0.856 ± 0.021 ; F1-Score = 0.879 ± 0.015; Precision = 0.823 ± 0.041; Recall = 0.944 ± 0.022; ROC-AUC = 0.964 ± 0.005<br><br>**CQ500**<br>**E2E-Att-GP**<br>Accuracy = 0.796 ± 0.018 ; F1-Score = 0.785 ± 0.017; ROC-AUC = 0.918 ± 0.003; Cohen's kappa = 0.594 ± 0.035<br>**E2E-GP-Att**<br>Accuracy = 0.756 ± 0.023 ; F1-Score = 0.736 ± 0.031; ROC-AUC = 0.912 ± 0.003; Cohen's kappa = 0.509 ± 0.048
Automated Intracranial Hemorrhage Detection Using Deep Learning in Medical Image Analysis	2024	Kar et al.	https://ieeexplore-ieee-org.ezproxy-b.deakin.edu.au/document/10691276		**PhysioNet**	82 patients	1. Resize (256x26)<br>2. Pixel normalization<br>3. Data augmentation	Utilizes a CNN with integrated attention mechanisms. The CNN is designed to efficiently extract hierarchical features from CT images. The attention modules are structured to guide the network's focus towards specific image regions indicative of hemorrhagic abnormalities.	Accuracy; Precision; Recall; F1-Score; AUC	Accuracy = 99.76%; Precision = 99.53%; Recall = 99.68%; F1-Score = 99.61%; AUC = 0.99
Dual-task vision transformer for rapid and accurate intracerebral hemorrhage CT image classification	2024	Fan et al.	https://www.nature.com/articles/s41598-024-79090-y	Proposes a dual-task vision transformer (DTViT) model for the simultaneous classification of normal vs. ICH images	**PRIVATE DATASET**	15,936 slices	1. Morphological treatment<br>2. Manual filtering<br>3. Data augmentation	Proposes a dual-task Vision Transformer (DTViT) model. DTViT utilizes the encoder of the Vision Transformer (ViT), which employs attention mechanisms for feature extraction from CT image patches. Transfer learning is employed by using a pre-trained ViT-Large model (trained on ImageNet-1K) as the backbone for the DTViT encoder.	Accuracy; Precision; Recall; F1-Score; Specificity	Accuracy = 0.9968; Precision = 0.9935; Recall = 0.9978; F1-Score = 0.9957; Specificity = 0.9962
Precise Image-level Localization of Intracranial Hemorrhage on Head CT Scans with Deep Learning Models Trained on Studylevel Labels	2024	Wu et al.	https://pmc-ncbi-nlm-nih-gov.ezproxy-b.deakin.edu.au/articles/PMC11605431/	Develops a weakly supervised deep learning architecture that combines transfer learning (TL) and long short-term memory (LSTM) networks that can automatically detect and localize intracranial hemorrhage (ICH).	**RSNA (training)**<br><br>**CQ500 (testing)**<br><br>**PRIVATE DATASET (fine-tuning):**<br>2008–2012 noncontrast head CT scans performed at Northwestern Memorial Hospital, Chicago.	674,258 images from 19,530 studies<br><br>491 patients<br><br>10,699 scans (7469 patients)	N/A	Develops a weakly supervised deep learning architecture that combines transfer learning (TL) and long short-term memory (LSTM) networks that can automatically detect and localize intracranial hemorrhage (ICH). The model was trained in three stages:<br>- Stage I: Pretraining an EfficientNet-B2 network on the image-level labeled RSNA dataset<br>- Stage II: Training an attention-based bidirectional LSTM network at the study level using the latent features extracted from the local dataset in Stage I. The LSTM aimed to capture interimage correlations<br>- Stage III: Fine-tuning the entire pipeline on the local institution's data using transfer learning to create an end-to-end model	Positive Predictive Value (PPV); AUC; Accuracy; F1-Score; Sensitivity; Specificity; Negative Predictive Value (NPV);	**Held-out Local Test:**<br>Positive Predictive Value (PPV) = 85.7%; AUC = 0.96; Accuracy = 93.7%; F1-Score = 84.9%; Sensitivity = 84.1%; Specificity = 96.3%; Negative Predictive Value (NPV) = 95.8%;<br><br>**External Test (CQ500):**<br>Positive Predictive Value (PPV) = 89.3%; AUC = 0.96; Accuracy = 91.6%; F1-Score = 89%; Sensitivity = 88.5%; Specificity = 89.6%; Negative Predictive Value (NPV) = 89.8%;
Automated intracranial hemorrhage detection in traumatic brain injury using 3D CNN	2023	Agrawal et al.	https://pmc-ncbi-nlm-nih-gov.ezproxy-b.deakin.edu.au/articles/PMC10696364/	Employs a 3D CNN model to detect ICH in CT scans.	**PRIVATE DATASET:**<br>CT scans were collected prospectively from the Department of Neurosurgery, All India Institute of Medical Sciences, New Delhi from January 2021 to September 2021.	200 scans (traing); 20 scans (test)	1. Resize (128x128)<br>2. Min-max normalization<br>3. Windowing (level 50, width 150)<br>4. Contract adjustment & Gaussian smoothing<br>4. Data augmentation	Employs a 3D CNN model to detect ICH in CT scans.	Accuracy; Sensitivity; Specificity	Accuracy = 80%; Sensitivity = 90%; Specificity = 70%
Deep Gaussian processes for multiple instance learning: Application to CT intracranial hemorrhage detection	2022	Pérez et al.	https://www.sciencedirect.com/science/article/pii/S0169260722001699#sec0008	Introduces a novel probabilistic model called Deep Gaussian Processes for Multiple Instance Learning (DGPMIL) for the detection of intracranial hemorrhage	**RSNA**<br><br>**CQ500**	39,750 slices<br><br>491 scans	1. Windowing<br>2. Normalization	Develops the Deep Gaussian Processes for Multiple Instance Learning (DGPMIL) model to detect ICH in CT scans. The proposed approach involves a two-phase training process:<br>1. Phase 1: Feature Extraction using Attention-based CNN (Att-CNN): A CNN with an attention mechanism is trained from scratch using the scan-level labels.<br>2. Phase 2: Classification using DGPMIL: The weights of the trained Att-CNN are frozen. The extracted slice-level features are then fed into the DGPMIL model for the final classification. DGPMIL uses L layers of Gaussian Processes to model the complex relationships in the extracted features. The model is trained using doubly stochastic variational inference by maximizing the Evidence Lower Bound (ELBO). The instance labels are treated as latent variables with an approximate posterior distribution that is iteratively updated. The bag label likelihood is parameterized based on the maximum predicted instance label in the bag. The model utilizes inducing points for scalability, and their locations are optimized per layer in DGPMIL.	Accuracy; F1-Score; Cohen's kappa; AUC-ROC; AUC-PR	**RSNA**<br>Accuracy = 0.825 ± 0.006; F1-Score = 0.839 ± 0.006; Cohen's kappa = 0.654± 0.011; AUC-ROC = 0.957 ± 0.011; AUC-PR = 0.961 ± 0.011<br><br>**CQ500**<br>Accuracy = 0.717 ± 0.035; F1-Score = 0.735 ± 0.022; Cohen's kappa = 0.461± 0.059; AUC-ROC = 0.909 ± 0.005; AUC-PR = 0.889 ± 0.011
Identification of intracranial haemorrhage (ICH) using ResNet with data augmentation using CycleGAN and ICH segmentation using SegAN	2022	Ganeshkumar et al.	https://link-springer-com.ezproxy-f.deakin.edu.au/article/10.1007/s11042-021-11478-8#Tab6	Develops a comprehensive one-stop model for the identification and segmentation of ICH	**PhysioNet**	82 scans	1. Resize (512x512)<br>2. Data augmentation with CycleGAN	To address the issue of class imbalance, a CycleGAN was trained to convert non-ICH CT slices into synthetic CT slices containing ICH. These data were then used to train a ResNet50 model for ICH detection.	Sensitivity; Specificity; Accuracy; F1-Score; AUC	Sensitivity = 0.80; Specificity = 0.99; AUC = 0.87; F1-Score = 0.91; Accuracy = 0.96
Intracerebral hemorrhage detection on computed tomography images using a residual neural network	2022	Altuve and Pérez	https://www-sciencedirect-com.ezproxy-f.deakin.edu.au/science/article/pii/S1120179722019901?via%3Dihub#ab005	Develop and evaluate a deep learning model for the automatic detection of ICH	**Kaggle's Head CT-Hemorrhage DB**	200 images	N/A	Utilizes transfer learning for ICH detection task by using a pre-trained ResNet-18 model that was initially trained on a subset of the ImageNet database.	Accuracy; Specificity; Sensitivity; Precision; F1-Score	Accuracy = 95.93%; Specificity = 96.20%; Sensitivity = 95.65%; Precision = 96.40%; F1-Score = 95.91%

Title	Year	Author	URL	Main Ideas	Dataset Details	Dataset Size	Data Preprocessing	Methods / Models	Metrics	Performance
Stacked deep model-based classification of the multiclass brain hemorrhages in CT scans	2024	Payal Malik and Ankit Vidyarthi	https://onlinelibrary-wiley-com.ezproxy-f.deakin.edu.au/doi/full/10.1002/ima.22955	Introduce a novel and specialized stacked deep model architecture tailored specifically for the multiclass classification of brain hemorrhages in CT scans.	**RSNA**	874,035 slices	1. Windowing<br>2. Data augmentation<br>3. Oversampling	Develops an ensemble of three pretrained deep learning models: SE-ResNeXt-50, EfficientNet B3, and EfficientNet B2 with weighted averaging fusion layer for classifying CT images into ICH subtypes (Subarachnoid, Intraparenchymal, Subdural, Epidural, Intraventricular)	Accuracy; Sensitivity; Specificity; AUC-ROC; Precision; Recall; F1-Score; Kappa	Accuracy = 0.9856; Sensitivity = 0.9589; Specificity = 0.9958; AUC-ROC = 0.9847; Precision = 0.983; Recall = 0.964; F1-Score = 0.985; Kappa = 0.9
A Novel Weighted Average 2D-CNN Ensemble for Intracranial Hemorrhage CT Image Classification	2024	Gudadhe and Thakare	https://ijettjournal.org/Volume-72/Issue-7/IJETT-V72I7P126.pdf	Develop a novel Weighted Average 2D-Convolutional Neural Network (WA2D-CNN) ensemble model for the classification of intracranial hemorrhage (ICH) subtypes	**PUBLIC DATASET (from Kaggle)**	36 patients (2510 images)	N/A	Proposes a Weighted Average 2D-CNN (WA2D-CNN) ensemble model for classifying ICH subtypes from CT scans. It is a combination of three distinct 2D-CNN models with different layered architectures (12 layers, 11 layers, and 9 layers) for feature extraction. Each model was assigned weights using a grid search approach.	Precision; Sensitivity; Accuracy; F1-Score	**Accuracy = 95.86%**<br><br>**ICH**<br>Precision = 89.52%; Sensitivity = 88.68%; Accuracy ; F1-Score = 89.10%<br><br>**SAH**<br>Precision = 96.34%; Sensitivity = 95.18%; Accuracy ; F1-Score = 95.76%<br><br>**EDH**<br>Precision = 95.17%; Sensitivity = 96.17%; Accuracy ; F1-Score = 95.67%<br><br>**Normal**<br>Precision = 96.15%; Sensitivity = 92.59%; [illegible]
Detection and Segmentation of Subdural Hemorrhage on Head CT Images	2024	Gencturk et al.	https://ieeexplore.ieee.org/document/10552268	Proposes a hybrid approach to brain hemorrhage detection fundamentally consists of two main components: segmentation and classification.	**CQ500**<br><br>**PRIVATE DATASET:** Collected at Niğde Ömer Halisdemir University Faculty of Medicine Neurosurgery Clinic and Cumhuriyet University Faculty of Medicine Neurosurgery Clinic	491 scans<br><br>2434 images	N/A	Proposes a hybrid model with two main stages:<br>1. Segmentation Stage: The MS R-CNN model is used to detect potential hemorrhage areas in the CT images and generate segmentation masks and bounding boxes. MS R-CNN utilizes a ResNet-101+FPN backbone for feature extraction and incorporates a Mask IoU (Intersection Over Union) component to improve mask quality.<br>2. Classification Stage: The EfficientNet-B2 architecture is employed to classify whether the regions segmented in the first stage truly contain hemorrhages. This stage acts as a second level of verification to reduce false positives. The EfficientNet-B2 model is trained using correctly identified hemorrhagic regions and healthy regions mistakenly identified as hemorrhagic by the segmentation model	Accuracy; Precision; Recall; F1-Score	**CQ500**<br>Accuracy = 94.301%; Precision = 90.426%; Recall = 97.701%; F1-Score = 93.923%<br><br>**PRIVATE DATASET**<br>Accuracy = 97.331%; Precision = 96.914%; Recall = 99.054%; F1-Score = 97.972%
Classification of Intracranial Hemorrhage Based on CT-Scan Image with Vision Transformer (ViT) Method	2024	Faiz et al.	https://ieeexplore-ieee-org.ezproxy-b.deakin.edu.au/document/10665827	Vision Transformer (ViT) modeling is carried out as an expert tool to classify ICH based on CT-Scan	**Refined RNSA CT Scans 12K**	12,000 images	1. Resize (256x256)<br>2. Grayscale<br>3. Data augmentation	Trains a ViT model to classify ICH subtypes on CT scans.	Accuracy	Accuracy = 81.2%
Automated Computer-Aided Detection and Classification of Intracranial Hemorrhage Using Ensemble Deep Learning Techniques	2023	Umapathy et al.	https://pmc-ncbi-nlm-nih-gov.ezproxy-f.deakin.edu.au/articles/PMC10527774/	Combines Squeeze and Excitation–based Residual Networks with the next dimension (SE-ResNeXT) and Long Short-Term Memory (LSTM) Networks for ICH subtypes classification	**RSNA**<br><br>**CQ500**	133,709 slices (train) + 14,600 slices (test)<br><br>491 scans	1. Windowing<br>2. Normalization<br>3. Augmentation	Develops an ensemble of Convolutional Neural Networks (CNNs) combining SE-ResNeXT and LSTM networks to classify the intracranial hemorrhage. SE-ResNeXT integrates a Squeeze-Excitation block with a Residual network and Inception network, utilizing shortcut connections and group convolutions managed by a cardinality hyperparameter to learn significant lesion features. LSTM networks are used to capture temporal dependencies or sequential information.	Accuracy; Precision; Recall; F1-Score; AUC	Accuracy = 94%; Precision = 0.96; Recall = 0.98; F1-Score = 0.97; AUC = 0.99
Intracranial hemorrhage detection in 3D computed tomography images using a bi-directional long short-term memory network-based modified genetic algorithm	2023	Sengupta et al.	https://pmc-ncbi-nlm-nih-gov.ezproxy-f.deakin.edu.au/articles/[illegible]	Classifying hemorrhage in 3D CT scans using DL	**RSNA**	25,272 images (870,301 slices)	N/A	The proposed framework consists of 4 main steps: [illegible]	F1-Score; MCC; Sensitivity; Specificity; Accuracy	[illegible]
An Efficient Framework to Detect Intracranial Hemorrhage Using Hybrid Deep Neural Networks	2023	Rajagopal et al.	https://pmc-ncbi-nlm-nih-gov.ezproxy-f.deakin.edu.au/articles/[illegible]		**RSNA**	752,044 slices	1. Windowing<br>2. Normalization	Proposes a hybrid Conv-LSTM model integrated with a Systematic Windowing technique for classifying ICH subtypes in CT scans.	Sensitivity; Specificity; Accuracy; Precision	[illegible]
Classification of brain hemorrhage computed tomography images using OzNet hybrid algorithm	2023	Ozaltin et al.	https://onlinelibrary-wiley-com.ezproxy-f.deakin.edu.au/doi/full/[illegible]	Develops an effective automatic classification algorithm to detect brain hemorrhage	**PhysioNet**	82 patients	N/A	Develops a novel Convolutional Neural Network (CNN) architecture named OzNet for feature extraction. [illegible]	Accuracy; Sensitivity; Specificity; Precision; F1-Score; G-Mean; AUC	**OzNet-NCA-SVM (Best results)**<br>[illegible]
Intracranial Hemorrhage Diagnosis Using Willow Catkin Optimization With Voting Ensemble Deep Learning on CT Brain Imaging	2023	Negm et al.	https://ieeexplore.ieee.org/document/[illegible]	Develops a new model (Willow Catkin Optimization with Voting Ensemble (ICHD-WCOVE) Model) for ICH classification	**PhysioNet**	82 patients [illegible]	N/A	Proposes the Willow Catkin Optimization with Voting Ensemble (ICHD-WCOVE) Model for ICH classification. [illegible]	Accuracy; Precision; Sensitivity; Specificity; F1-Score	[illegible]
Fine-grained classification of intracranial haemorrhage subtypes in head CT scans	2023	Liu et al.	https://ietresearch.onlinelibrary.wiley.com/doi/[illegible]	Proposes a classification algorithm based on CNN-RNN architecture for ICH subtypes	**RSNA**	25,272 scans (870,301 slices)	1. Remove duplicate or invalid samples<br>2. Windowing (brain, subdural, soft tissue)<br>3. Resize (256x256)<br>4. Remove bones	Employs a CNN-RNN architecture for ICH subtypes classification. [illegible]	Hamming Loss; Accuracy; Precision; Recall; F1-Score	[illegible]
Intracranial Hemorrhage Detection Using Parallel Deep Convolutional Models and Boosting Mechanism	2023	Asif et al.	https://www.mdpi.com/2075-4418/13/4/652	Presents a new methodology to improve the detection and subtype classification of ICH based on two parallel paths and a boosting technique	**RSNA**<br><br>**CQ500 (test)**	[illegible]<br><br>491 scans	1. Windowing<br>2. Skull removal<br>3. Data augmentation	[illegible] computed tomography (CT) scans, followed by a Light Gradient Boosting Machine (LGBM) for detection and subtype classification.	AUC; Sensitivity; Specificity; Precision; F1-Score; AUPR; Accuracy	[illegible] Score = 0.963; AUPR = 0.972; Accuracy = 0.985<br><br>**EDH**<br>AUC = 0.980; Sensitivity = 0.972; Specificity = 0.963; Precision = 0.966; F1-Score = 0.952; AUPR = 0.964; Accuracy = 0.975<br><br>**IPH**<br>AUC = 0.979; Sensitivity = 0.968; Specificity = 0.982; Precision = 0.973; F1-Score = 0.972; AUPR = 0.965; Accuracy = 0.987
A Double-Branch Xception Architecture for Acute Hemorrhage Detection and Subtype Classification	2023	Akram et al.	https://www.techscience.com/cmc/v76n3/54380/html		**RSNA**	180,000 (training); 12,000(validation); 100,000(testing)	1. Windowing<br>2. Normalization<br>3. ROI extraction<br>4. Skull removal	Proposes a double-branch model based on xception architecture. One branch processes 3D images formed by concatenating grayscale images from the three different intensity windows. The other branch analyzes neighboring slices with the skull removed, linking them to create a 3D spatial context. Those features are then concatenated and fed to a decision tree classifier for final predictions.	Accuracy; Precision; Recall; Specificity; F1-Score	**EPH**<br>Accuracy = 99.73%; Precision = 99.98%; Recall = 97.01%; F1-Score = 98.47%<br><br>**IPH**<br>Accuracy = 99.10%; Precision = 96.01%; Recall = 92.40%; F1-Score = 94.16%<br><br>**IVH**<br>Accuracy = 99.49%; Precision = 96.10%; Recall = 90.01%; F1-Score = 92.95%<br><br>**SAH**<br>Accuracy = 99.49%; Precision = 96.10%; Recall = 90.01%; F1-Score = 92.95%<br><br>**SDH**<br>Accuracy = 98.09%; Precision = 96.01%; Recall = 94.04%; F1-Score = 95.01%<br><br>**Any**<br>Accuracy = 97.35%; Precision = 98.0%; [illegible]
Transfer Learning of the ResNet-18 and DenseNet-121 Model used to Diagnose Intracranial Hemorrhage in CT Scanning	2022	Zhou et al.	https://www.eurekaselect.com/article/119384/eyJpdiI6IjM3b2RGelZENWM0aXNlTTdyQ0RzSHc9PSIsInZhbHVlIjoiSzBwTVNFYmZVZksxS1NuLzZBZjk3ZzI3MkovNktRZGE2WS81QlI5WjRBNmtNOTBabkZ2allzVkFyS3I5Qm1NVERFRjBqRUxWeENwRGhEVlRsSFRGdWh3TlI2M1hlblVtc2ZiMm1DMVM2akhmOXFGQTl4eW9Eb1VTbXpsczlmU0RnZHNEcmVqK1hzdk8rY2dlTnU1aHd3PT0iLCJtYWMiOiIxMWE4MjQzN2Y0YTc5MDZkNWVlMmQzNzQwZWEwMWE0MmRiZWYwOTU3ZTlmMjc2ZjVjZjMwOWU3NmM2NzRiZmZlIiwidGFnIjoiIn0=	Uses ResNet-18 and DenseNet-121 for classifying ICH subtypes	**PRIVATE DATASET:**<br>Patients at People's Hospital of Guangxi from March 2018 to March 2020	2768 images	1. Resize (512x512)	Employs two pre-trained convolutional neural network (CNN) models, ResNet-18 and DenseNet-121, with transfer learning to classify ICH subtypes in CT scans.	Sensitivity; Specificity; AUC	**ResNet-18**<br>**Normal**<br>Sensitivity = 1.00; Specificity = 0.87; AUC = 1.00<br><br>**EDH**<br>Sensitivity = 0.98; Specificity = 0.88; AUC = 1.00<br><br>**IVH**<br>Sensitivity = 0.85; Specificity = 0.91; AUC = 0.96<br><br>**CPH**<br>Sensitivity = 0.8; Specificity = 0.91; AUC = 0.96<br><br>**SAH**<br>Sensitivity = 0.81; Specificity = 0.91; AUC = 0.94<br><br>**SDH**<br>Sensitivity = 0.93; Specificity = 0.89; AUC = 0.98<br><br>**DenseNet-121**<br>**Normal**<br>Sensitivity = 0.98; Specificity = 0.79; AUC = 1.00
Intracranial Hemorrhages Segmentation and Features Selection Applying Cuckoo Search Algorithm with Gated Recurrent Unit	2022	Sengupta et al.	https://www.mdpi.com/2076-3417/12/21/10851	Presents a novel automated model called OGRU-CSO for the detection and classification of intracranial hemorrhages	**RSNA**	25,272 scans (870,301 slices)	N/A	Proposes an automated model called OGRU-CSO for the detection and classification of ICH in 3D CT scans. It aims to improve classification accuracy and reduce computational time by integrating Fuzzy C Means (FCM) clustering for segmentation, hybrid feature extraction using Histogram of Oriented Gradients (HoG), Local Ternary Pattern (LTP), and Local Binary Pattern (LBP), feature optimization with the Cuckoo Search Optimization (CSO) algorithm, and classification with an Optimized Gated Recurrent Unit (OGRU)	Accuracy; Recall; Specificity	Accuracy = 99.36%; Recall = 99.25%; Specificity = 99.40%
An optimal deep learning framework for multi-type hemorrhagic lesions detection and quantification in head CT images for traumatic brain injury	2022	Phaphuangwittayakul et al.	https://link.springer.com/article/10.1007/s10489-021-02782-9	Integrates a fine-tuned multi-label classification model (for identifying ICH subtypes) with an optimal multi-class semantic segmentation model (for precisely delineating hemorrhage regions) and a novel quantitative assessment algorithm (for measuring thickness and volume)	**RSNA**<br><br>**PhysioNet**<br><br>**PRIVATE DATASET:**<br>Patients were collected from Maharaj Nakorn Chiang Mai Hospital.	75,5948 slices<br><br>82 scans (2814 slices)<br><br>321 patients (19,946 slices)	1. Windowing<br>2. Resize (256x256)<br>3. Data augmentation<br>4. Normalization	Develops an optimized DeepMedic model, which is a 3D CNN network for the semantic segmentation of different ICH subtypes on the CT scans.This model generates 3D masks where each pixel is classified into a specific hemorrhage subtype or background.	Dice; Jaccard; Precision; Sensitivity; Specificity; F1-Score; Accuracy	Jaccard = 0.777 ± 0.403; Dice = 0.784 ± 0.399; Precision = 97.96%; Sensitivity = 95.77%; Specificity = 96.90%; F1-Score = 96.85%; Accuracy = 96.21%
Detection and categorization of acute intracranial hemorrhage subtypes using a multilayer DenseNet-ResNet architecture with improved random forest classifier	2022	Jenefer et al.	https://onlinelibrary-wiley-com.ezproxy-f.deakin.edu.au/doi/full/10.1002/cpe.7167	Develops a highly accurate and computationally efficient method for the detection and categorization of ICH subtypes	**PhysioNet**	82 scans	1. Gaussian filter	Proposes ICH-ResNet-DenseNet-IRF framework, which involves the following modules:<br>1. A multilayer DenseNet combined with a ResNet architecture is employed to extract relevant features from the preprocessed images<br>2. An improved random forest (IRF) classifier is used to classify the extracted features into the five subtypes of ICH (EDH, SAH, IPH, SDH, IVH).	Accuracy; Specificity; Sensitivity; Precision; F1-Score; Recall	**SDH**<br>Accuracy = 99.53%; Specificity = 93.86%; Sensitivity = 91.86%; Precision = 96.44%; F1-Score = 95.45%; Recall = 92.03%<br><br>**EDH**<br>Accuracy = 98.75%; Specificity = 90.53%; Sensitivity = 93.87%; Precision = 95.61%; F1-Score = 94.86%; Recall = 91.92%<br><br>**IPH**<br>Accuracy = 98.09%; Specificity = 91.75%; Sensitivity = 90.45%; Precision = 94.43%; F1-Score = 92.64%; Recall = 93.92%<br><br>**IVH**<br>Accuracy = 99.8%; Specificity = 93.86%; Sensitivity = 93.97%; Precision = 97.96%; F1-Score = 93.66%; Recall = 92.93%<br><br>**SAH**<br>Accuracy = 99.46%; Specificity = 95.87%; Sensitivity = 92.33%; Precision = 94.66%; F1-Score = 90.65%; Recall = 89.02%
Intracranial hemorrhage subtype classification using learned fully connected separable convolutional network	2022	Korra et al.	https://onlinelibrary-wiley-com.ezproxy-f.deakin.edu.au/doi/full/10.1002/cpe.7218	Introduces a novel learned fully connected separable convolutional network for the automated classification of intracranial hemorrhage subtypes	**RSNA**	25,272 scans (870,301 slices)	1. Windowing<br>2. Resize (256x256)<br>3. Data augmentation	Proposes a learned fully connected separable convolutional network for ICH subtypes classification. It consists of four layers: two fully connected layers and two convolutional layers (depth-wise and point-wise), totaling 13 layers with components like dropout, ReLU activation, softmax, and max pooling	Accuracy; Specificity; Sensitivity; AUC	Accuracy = 98.63%; Specificity = 99.49%; Sensitivity = 73.32%; AUC = 98.98%
An Attention-Based ResNet Architecture for Acute Hemorrhage Detection and Classification: Toward a Health 4.0 Digital Twin Study	2022	Hussain et al.	https://ieeexplore.ieee.org/document/9966583	Develops an attention-based ResNet architecture for the detection and classification of ICH subtypes	**RSNA**	25,272 scans (870,301 slices)	1. Resize (224x224)<br>2. Convert to PNG<br>3. Windowing<br>4. Normalization<br>5. Skull removal<br>6. Data augmentation with DCGAN	Proposes a novel hybrid model incorporating an attention-based ResNet-152V2 architecture for focused feature extraction, Principal Component Analysis (PCA) for dimensionality reduction and redundant feature removal, and a XGBoost classifier to predict the subtype of ICH.	Accuracy; Recall; Specificity; F1-Score; AUC	**EDH**<br>Accuracy = 99.2%; Recall = 97.1%; Specificity = 96.4%; F1-Score = 96.1%; AUC = 98.3%<br><br>**IPH**<br>Accuracy = 97.1%; Recall = 94.9%; Specificity = 95.1%; F1-Score = 94.3%; AUC = 92.9%<br><br>**IVH**<br>Accuracy = 96.7%; Recall = 95.4%; Specificity = 92.1%; F1-Score = 92.7%; AUC = 94.1%<br><br>**SDH**<br>Accuracy = 96.7%; Recall = 96.4%; Specificity = 91.4%; F1-Score = 95.5%; AUC = 95.0%<br><br>**SAH**<br>Accuracy = 96.1%; Recall = 95.6%; Specificity = 90.8%; F1-Score = 92.1%; [illegible]
Detecting hemorrhage types and bounding box of hemorrhage by deep learning	2022	Ertugrul and Akil	https://www-sciencedirect-com.ezproxy-f.deakin.edu.au/science/article/pii/S174680942100682O?via%3Dihub	Uses YOLOv4 to detect and localize different types of ICH in CT scans	**BHX**	23,409 images (39,668 bounding boxes)	1. Windowing<br>2. Noise removal (morphology dilation)	Trains YOLOv4 to detect and localize different types of ICH in CT scans.	Precision; Recall; F1-Score	Precision = 94%; Recall = 92%; F1-Score = 93%
A joint convolutional-recurrent neural network with an attention mechanism for detecting intracranial hemorrhage on noncontrast head CT	2022	Alis et al.	https://www.nature.com/articles/s41598-022-05872-x	Uses a joint convolutional neural network-recurrent neural network (CNN-RNN) with an attention mechanism for detecting and classifying ICH subtypes	**PRIVATE DATASET**	55,179 scans	1. Windowing<br>2. Normalization<br>3. Resize (480x480)<br>4. Data augmentation	Introduces a novel joint CNN-RNN with an attention mechanism, for automated detection and subcategorization of ICH on CT scans. InceptionResNetV2 was used as the base CNN for feature extraction from the head CT images	Sensitivity; Specificity; Precision; Accuracy; AUC-ROC	**ICH**<br>Sensitivity = 99.70%; Specificity = 99.34%; Precision = 97.24%; Accuracy = 99.41%; AUC-ROC = 0.998<br><br>**IPH**<br>Sensitivity = 95.90%; Specificity = 99.28%; Precision = 95.03%; Accuracy = 98.85%; AUC-ROC = 0.998<br><br>**IVH**<br>Sensitivity = 95.22%; Specificity = 99.56%; Precision = 94.99%; Accuracy = 99.21%; AUC-ROC = 0.998<br><br>**SAH**<br>Sensitivity = 84.50%; Specificity = 99.09%; Precision = 92.35%; Accuracy = 97.41%; AUC-ROC = 0.991<br><br>**SDH**<br>Sensitivity = 91.13%; Specificity = 99.33%; Precision = 92.78%; Accuracy = 98.62%; AUC-ROC = 0.974<br><br>**EDH**<br>Sensitivity = 74.61%; Specificity = 98.83%; Precision = 51.56%; Accuracy = 98.50%;

Title	Year	Author	URL	Main Ideas	Dataset Details	Dataset Size	Data Preprocessing	Methods / Models	Metrics	Performance
TL-LFF Net: transfer learning based lighter, faster, and frozen network for the detection of multi-scale mixed intracranial hemorrhages through genetic optimization algorithm	2024	Kothala and Guntur	https://link-springer-com.ezproxy-f.deakin.edu.au/article/10.1007/s13042-024-02324-y	Develops a lighter and faster model for categorizing multi-scale mixed hemorrhages in a specific CT	**Brain Hemorrhage Extended (BHX) dataset** (extension of the qure.ai CQ500 dataset)	491 patients (21,132 slices from 205 positive patients)	1. Windowing<br>2. Data augmentation	Proposes a novel Transfer Learning-based Lighter, Faster, and Frozen (TL-LFF) Network. The architecture builds upon a single-stage object detection framework, similar to YOLO. The source model, pre-trained on ImageNet, consists of three main modules: backbone (based on CSPDarknet53 with C3 layers and a Spatial Pyramid Pooling Faster (SPPF) layer), neck (Path Aggregated Network - PANet), and head. To achieve a lightweight nature, the backbone and neck sections of the source model are frozen during the backpropagation stage of training the target model. Transfer learning is applied to improve the accuracy of the frozen network. Additionally, a Genetic Algorithm (GA) is used to optimize the hyperparameters of the model, with mutation employed to generate new hyperparameter sets based on previous generations' best performers.	Specificity; Accuracy; False Positive Rate (FPR); False Negative Rate (FNR); Matthew's Correlation Coefficient (MCC); Precision; Recall; F1-Score; mAP	Specificity = 0.993; Accuracy = 0.987; FPR = 0.005; FNR = 0.02; MCC = 0.968; Precision = 0.935; Recall = 0.945; F1-Score = 0.940; mAP@.5 = 0.964; mAP@.5:.95 = 0.728
Deep multiscale convolutional feature learning for intracranial hemorrhage classification and weakly supervised localization	2024	He et al.	https://www.sciencedirect.com/science/article/pii/S2405844024063011	Presents a novel deep multiscale convolutional feature fusion framework for accurate classification and weakly supervised localization of intracranial hemorrhage (ICH) and its subtypes in brain CT scans	**PUBLIC DATASET:**<br><br>**ASNR (Through Kaggle)**	750,000 scans	1. Windowing	Proposes a multiscale feature classification and weakly supervised localization framework. The proposed framework consists of three key modules:<br>1. Window Tuning Optimization Module: This module is designed to enhance the visibility of ICH in CT images by mimicking the way radiologists adjust window width and level. It replaces the initial layer of the VGG-16 backbone network and uses a 1x1x3 convolution layer with a custom activation function that has learnable parameters. This allows the model to dynamically adjust the window settings during training to better capture relevant features from the original Dicom image information.<br>2. Multiscale Feature Fusion Module: To handle the varying sizes and appearances of different ICH types, this module integrates features from different convolutional layers (C3, C4, C5) of the VGG-16 network. It employs channel and spatial attention mechanisms to aggregate these multiscale features, enabling the model to focus on important lesion areas, including smaller ones. A Squeeze-and-Excitation (SE) module is used for channel attention to recalibrate channel interdependencies, followed by a spatial attention module to further integrate spatial information.	AUC	AUC = 0.973
YOLOv5s-CAM: A Deep Learning Model for Automated Detection and Classification for Types of Intracranial Hematoma in CT Images	2023	Vidhya et al.	https://ieeexplore-ieee-org.ezproxy-f.deakin.edu.au/document/10345567	develop an automated deep learning model (YOLOv5s-CAM) for the detection and classification of six types of ICH	**BHX Dataset (extended version of CQ500)**	491 scans (39,668 bounding boxes)	1. Windowing	Proposes YOLOv5s-CAM, which is based on the baseline YOLOv5s model with several key improvements:<br>1. Cascaded Attention Module (CAM): A novel CAM is introduced in the neck of the YOLOv5s model. This module consists of the Efficient Channel Attention Module (ECA) placed in series with the Convolutional Block Attention Module (CBAM). The ECA focuses on significant channels, while CBAM refines the features by considering both channel and spatial information. The weighted feature map from CAM is then concatenated with the input feature maps.<br>2. Spatial Pyramid Pooling-Fast (SPPF): The original Spatial Pyramid Pooling (SPP) module in the YOLOv5s backbone is replaced with SPPF. SPPF uses serial max-pooling operations with a kernel size of 8 × 8, which reduces computational parameters, improves computational speed, and enhances prediction accuracy for hematoma.	Precision; Recall; F1-Score; mAP	Precision = 0.935; Recall = 0.908; F1-Score = 0.921; mAP@.5 = 0.943; mAP@.5:.95 = 0.65
Localization of mixed intracranial hemorrhages by using a ghost convolution-based YOLO network	2023	Kothala et al.	https://www-sciencedirect-com.ezproxy-f.deakin.edu.au/science/article/pii/S1746809422008321	Proposes a solution to detect multiple hemorrhages with limited resources by employing a ghost convolution process.	**BHX Dataset (extended version of CQ500)**<br><br>**PRIVATE DATASET**	491 scans (205 positive, 21,132 slices)<br><br>75 patients (2,500 slices - 318 slices positive)	1. Windowing (brain, subdural, bone)<br>2. Resize (256x256)<br>3. Data augmentation	Proposes a YOLOv5x-GCB model, which is based on the baseline YOLOv5x model, for object detection of mixed ICH. The methodology involves the following key components:<br>1. Ghost Convolution (GC) Module: To reduce computational complexity and model size, the researchers incorporated GC modules. GC generates more feature maps from a smaller number of intrinsic feature maps using inexpensive linear operations.<br>2. C3Ghost Module: The original C3 modules in the YOLOv5 backbone and neck are replaced with C3Ghost modules, which utilize ghost bottleneck (GB) structures to extract features efficiently	Precision; Recall; F1-Score; mAP	Precision = 0.921; Recall = 0.889; F1-Score = 0.900; mAP@.5 = 0.931; map@.5:.95 = 0.624
Study of intracranial haematoma localisation based on improved RetinaNet	2023	Cheng et al.	https://dl-acm-org.ezproxy-f.deakin.edu.au/doi/fullHtml/10.1145/3599589.3599601	Proposes an improved model based on RetinaNet for hematoma localization	N/A	N/A	N/A	Optimizing the original RetinaNet model in three key aspects:<br>1. Feature Extraction Network Structure: The original RetinaNet was improved by integrating attentional gates (AGs) to enable the network to focus on the target structures in the CT images by generating attention coefficients. This process prunes less relevant features, leaving only the activations pertinent to the task of haemorrhage localisation.<br>2. Anchor Settings: The default anchor sizes and aspect ratios in RetinaNet were deemed unsuitable for the characteristics of intracranial haemorrhagic lesions. Therefore, a differential evolutionary search algorithm was employed to optimise the ratio and proportion of anchor points on the validation set. The goal was to maximise the overlap between the lesion bounding boxes and the best anchors.<br>3. Training Techniques: To address the issue of imbalanced positive and negative samples common in single-stage target detection, the study utilised an improved Focal Loss function. The Focal Loss function is a modification of the cross-entropy loss designed to reduce the impact of easily classified samples and focus the training on hard-to-classify ones. The weighted cross-entropy loss function was first introduced, and then the final Focal	mAP; AUC; AP	mAP = 0.38<br><br>**ICH**<br>AUC = 0.956; AP = 0.46<br><br>**IVH**<br>AUC = 0.965; AP = 0.17<br><br>**SDH**<br>AUC = 0.972; AP = 0.49<br><br>**SAH**<br>AUC = 0.948; AP = 0.41

Title	Year	Author	URL	Main Ideas	Dataset Details	Dataset Size	Data Preprocessing	Methods / Models	Metrics	Performance
Multi-scale object equalization learning network for intracerebral hemorrhage region segmentation	2024	Zhang et al.	https://www-sciencedirect-com.ezproxy-b.deakin.edu.au/science/article/pii/S0893608024004313?via%3Dihub	Propose a multi-scale object equalization learning network for ICH region segmentation	**PRIVATE DATASET**	460 CT scans + 480 CT scans	1. HU range [0-100] to eliminate extraneous elements<br>2. Min-max normalization	MOEL-Net: Uses a shallow feature extraction module (SFEM) for obtaining shallow semantic representations to maintain sufficient and effective detailed location information. Then, a deep feature extraction module (DFEM) is leveraged to extract the deep semantic information of the ICH region from the combination of SFEM and original image features. The fused feature from SFEM and DFEM is then fed to a multi-level semantic feature equalization fusion module (MSFEFM) for obtaining a balanced representation of multi-level semantic features. The segmentation map is obtained by combining the output features of SFEM, DFEM and MSFEFM.	Dice; Precision; Jaccard; Sensitivity; Specificity	**Dataset 1:**<br>Dice = 0.9092; Precision = 0.9246; Jaccard = 0.8367; Sensitivity = 0.9002; Specificity = 0.9997<br><br>**Dataset 2:**<br>Dice = 0.9095; Precision = 0.9343; Jaccard = 0.8360; Sensitivity = 0.8895; Specificity = 0.9997
Fuzzy C-means clustering algorithm applied in computed tomography images of patients with intracranial hemorrhage	2024	Zhang et al.	https://www.frontiersin.org/journals/neuroinformatics/articles/10.3389/fninf.2024.1440304/full	Propose an improved fuzzy C-means (FCM) algorithm for ICH detection and segmentation	**CT-ICH**	256 images	1. Windows: brain (level 40, width 120), and skull (level 700, width 120)<br>2. Data augmentation	The study introduces a kernel function to measure pixel distances in a higher-dimensional space, transitioning from a two-dimensional hybrid algorithm to a three-dimensional one to better segment the ICH.	Dice; RMSE	Dice = 0.91; RMSE = 0.097
Using Natural Language Processing (GPT-4) for Computed Tomography Image Analysis of Cerebral Hemorrhages in Radiology: Retrospective Analysis	2024	Zhang et al.	https://pmc.ncbi.nlm.nih.gov/articles/PMC11467597/	Using NLP to annotate and analyze CT images	**PRIVATE DATASET**<br>CT scans with 6 types of cerebral hemorrhages at Ren Ji Hospital, Shanghai Jiao Tong University School of Medicine, between January and September 2023	208 scans	1. Windows: level 35, width 90<br>2. Reformat to JPG<br>3. Resize to 700x700	Uses GPT-4 with a predefined question-and-answer format to generate annotations for ICH segments on CT scans.	Identification completeness percentage (CP): (correct annotated area / total bleeding area) × 100%; Misidentification percentage (MP): (total annotated area – correct annotated area) / total annotated area × 100%	**CP:**<br>epidural: 89.0%; intraparenchymal: 86.9%; acute subdural: 74.4%; subarachnoid: 71.5%; complex: 76.4%; chronic subdural: 37.3%<br><br>**MP:**<br>epidural: 50.2%; intraparenchymal: 26.2%; acute subdural: 32.6%; subarachnoid: 50.5%; complex: 54%; chronic subdural: 40.3%
Automatic Segmentation of Intracranial Hemorrhage in Computed Tomography Scans with Convolution Neural Networks	2024	Xu et al.	https://link-springer-com.ezproxy-b.deakin.edu.au/article/10.1007/s40846-024-00892-6	Utilize a two-staged cascaded encoder-decoder convolutional neural network (CNN) to automatically segment ICH and estimate its volume from CT scans	**PRIVATE DATASET**	401 scans (train: 280 scans, test: 121 scans)	1. Data augmentation	Proposes a two-staged encoder-decoder convolution neural network, which segments the coarse hemorrhage area in the first stage and the fine hemorrhage area in the second stage. With the segmentation result, the volume of hemorrhage is further estimated.	Dice, Recall	Dice = 84.54%; Recall = 86.03%
Multi-scale Perception and Feature Refinement Network for multi-class segmentation of intracerebral hemorrhage in CT images	2024	Xiao et al.	https://www-sciencedirect-com.ezproxy-b.deakin.edu.au/science/article/pii/S1746809423010479?via%3Dihub	Introduces a novel deep learning network called the Multi-scale Perception and Feature Refinement Network (MPFR-Net) for the multi-class segmentation of intracerebral hemorrhage (ICH) in CT images	**PRIVATE DATASET**<br><br>**PhysioNet (for evaluation)**	18,386 slices<br><br>82 patients	N/A	Proposes MPFR-Net, which leverages multi-scale feature perception to handle different sizes of hemorrhages and feature refinement to recover detailed information and distinguish between IPH and IVH. The core of the network consists of two main modules: Multi-scale Perception Module (MPM) concatenates deep features from the encoder and then employs both global and local branches to extract multi-scale features; and Feature Refinement Module (FRM) fuses the multi-scale features from MPM with shallow features from the encoder to refine fuzzy details and supplement small target information.	Dice	IVP: 0.7721; IPH: 0.8799; ICH: 0.9074; Avg = 0.8260
DFMA-ICH: a deformable mixed-attention model for intracranial hemorrhage lesion segmentation based on deep supervision	2024	Xiao et al.	https://link-springer-com.ezproxy-b.deakin.edu.au/article/10.1007/s00521-024-09545-w	Proposes a Deformable Mixed-Attention Model for ICH lesion Segmentation (DFMA-ICH) to address challenges with small bleeding lesions and blurred boundaries.	**PRIVATE DATASET**<br>**Spontaneous ICH Dataset:** 207 scans were retrospectively collected from Taihu Hospital in Wuxi, China (NCCT, 512x512 resolution, 5 mm layer thickness, 0.45-0.55 mm² pixel spacing); 100 scans were from the public INSTANCE 2022 challenge (NCCT) from Shougang Hospital, Peking University<br><br>**Traumatic ICH Dataset:** Comprised of 80 patients' CT data of traumatic ICH from Daping Hospital in Chongqing, China (512x512 resolution), including SDH and EDH subtypes	307 scans<br><br>80 patients	1. Convert to NiFTi<br>2. Window width = 100; Window level = 65 (85-70 for set 2)<br>3. Slices/masks converted to PNG<br>4. Data augmentation	DFMA-ICH consists of three paths: a feature extraction path (based on the lightweight STDC backbone with a mixed-attention method), a feature fusion path (MFFM combining mixed attention and deformable convolution), and a deep supervision training path, which extract, fuse, and supervise features at different levels while training to improve the performance and generalization ability of the model	Dice; IoU; Hausdorff Distance (HD); Recall; F1-Score	**Spontaneous ICH Dataset:**<br>DSC = 86.03% ± 12.24%; IoU = 76.98% ± 13.95%; HD = 12.35mm ± 14.75mm; Recall = 83;33% ± 13.98%; F1-score = 84.94% ± 11.35%<br><br>**Traumatic ICH Dataset:**<br>DSC = 80.98% ± 11.68%; IoU = 69.91% ± 15.14%; HD = 47.28mm ± 45.79mm; Recall = 80.52% ± 13.36%; F1-score = 80.04% ± 13.83%
Spatial attention-based CSR-Unet framework for subdural and epidural hemorrhage segmentation and classification using CT images	2024	Nafees Ahmed and Prakasam	https://pmc-ncbi-nlm-nih-gov.ezproxy-f.deakin.edu.au/articles/PMC11494839/	Proposes a rich segmentation approach to segment both SDH and EDH	**PhysioNet**	82 patients	1. Resize<br>2. Denoise & Enhance Contrast<br>3. Class balancing (SMOTE)<br>4. Gamma correction	Proposes a Spatial attention-based CSR Unet framework for segmenting and classifying SDH and EDH from CT images. This is a U-shaped encoder-decoder network that incorporates CSR (Convolution-Squeeze Excitation-Residual) modules in the encoder to improve feature extraction	Recall; Precision; F1-Score; Accuracy; Dice; IoU	**SHD**<br>Dice = 0.969; IoU = 0.687; Recall = 1.0; Precision = 1.0; F1-Score = 1.0; AUC-RoC; Accuracy = 1.0;<br><br>**EHD**<br>Dice = 0.983; IoU = 0.749; Recall = 1.0; Precision = 1.0; F1-Score = 1.0; AUC-RoC; Accuracy = 1.0;
Two-Stage Approach to Intracranial Hemorrhage Segmentation From Head CT Images	2024	Rajapakse et al.	https://ieeexplore.ieee.org/document/10507829	Introduces a novel two-stage approach for intracranial hemorrhage (ICH) segmentation in head CT images	**CQ500**<br><br>**RSNA**	491 scans (193,317 slices)<br><br>874,035 slices	1. Resize (512x512)<br>2. Windowing<br>3. Pixels scale to 0-255<br>4. Data augmentation	Proposes a two-stage approach that utilizes both bounding boxes and segmentation masks to help improve segmentation performance. In the first stage, ICH regions are detected and localized with bounding boxes surrounding the lesion by using a supervised YOLOv5 object detector. In the second stage, the localized ICH foreground is automatically segmented using TransDeepLab, an attention-based transformer network.	Dice; IoU	Dice = 0.605; IoU = 0.478
DDANet: A deep dilated attention network for intracerebral haemorrhage segmentation	2024	Liu et al.	https://ietresearch-onlinelibrary-wiley-com.ezproxy-f.deakin.edu.au/doi/full/10.1049/syb2.12103	Introduces DDANet, a novel haematoma segmentation model for brain CT images. The main goal is to enhance the speed and precision of ICH detection, particularly for small or subtle haemorrhages, by addressing challenges such as the variability in the shape, size, and location of haemorrhages, as well as low contrast with surrounding tissue	**PhysioNet**	82 patients	N/A	Proposes DDANet, which is an enhanced U-Net framework consisting of feature extraction and feature aggregation stages. For feature extraction, the model employs a pre-trained ResNet-34 as the encoder. A novel dilated convolution pooling (DCP) block is introduced in the intermediate layers of the encoder (excluding the first level) to enhance the receptive field and capture multiscale contextual information of ICHs of varying sizes. Additionally, a self-attention module (SAM) is incorporated to capture global semantic information of high-level features and guide the processing of low-level features, improving the model's understanding of the overall structure while preserving details. For feature aggregation in the decoding phase, DDANet integrates residual networks, channel attention, and spatial attention mechanisms for joint optimization, effectively mitigating the class imbalance problem and aiding the training process	Dice, Jaccard; Sensitivity; Specificity; Accuracy	Dice = 0.712 ± 0.223, Jaccard = 0.601 ± 0.240; Sensitivity = 0.730 ± 0.210; Specificity = 0.998 ± 0.002; Accuracy = 0.997 ± 0.002
Semi-supervised Learning for Generalizable Intracranial Hemorrhage Detection and Segmentation	2024	Lin et al.	https://pubs.rsna.org/doi/full/10.1148/ryai.230077	Proposes using a semi-supervised learning (SSL) approach, specifically the noisy student paradigm, to improve the generalizability of these models by leveraging both labeled and unlabeled data	**Atlantis-labeled dataset**<br><br>**Kaggle-25K (unlabeled)**<br><br>**PRIVATE DATASET (validation)**<br><br>**CQ500 (testing)**	457 scans<br><br>25K scans<br><br>93 scans<br><br>491 scans	1. Data augmentation	Employs the noisy student semi-supervised learning paradigm. This involved the following steps:<br>1. Training a "teacher" model: A deep learning model (PatchFCN) was initially trained from scratch on the small Atlantis pixel-labeled dataset.<br>2. Generating pseudo labels: The trained teacher model was used to generate pixel-level and image-level pseudo labels (predictions) on the large unlabeled Kaggle-25K dataset<br>3. Ranking pseudo-labeled images: The unlabeled images were ranked based on the teacher's predicted probability of hemorrhage<br>4. Applying a threshold (Ranker): A threshold was applied to these probabilities. Only the top 10% of images with the highest probability were considered positive. For these positive images, pixels with high confidence were labeled positive, and those with low confidence negative, while intermediate confidence pixels were ignored during training. All other images were considered negative with all pixel predictions set to 0.<br>5. Training a "student" model: A second "student" model (also PatchFCN) was initialized with the weights of the teacher model and trained on the combination of	AUC; Dice; Pixel Average Precision (AP)	AUC = 0.939; Dice = 0.829; AP = 0.848
Deep learning models for separate segmentations of intracerebral and intraventricular hemorrhage on head CT and segmentation quality assessment	2024	Li et al.	https://aapm-onlinelibrary-wiley-com.ezproxy-f.deakin.edu.au/doi/10.1002/mp.17343	Develops a robust deep learning model for the segmentation of ICH and IVH	**PRIVATE DATASET:**<br><br>**ShanDong (SD) dataset:** The data was collected between July 2013 and October 2020 at Qilu Hospital of Shandong University Dezhou Hospital<br><br>**TianJin (TJ) dataset (testing):** The data was collected between January 2015 and August 2019 at Tianjin Medical University General Hospital	977 images<br><br>375 images	1. Brain extraction<br>2. Clipping<br>3. Normalization<br>4. Resampling	Develops REUnet, a 3D convolutional neural network based on the nnU-Net framework but incorporating residual connections in its encoding blocks for segmenting ICH and IVH.	Dice; Sensitivity; Precision; Absolute Volume Difference (AVD); Absolute Volume Difference Ratio (AVDR)	Dice = 0.777; Sensitivity = 0.707; Precision = 0.910; AVD = 1.312; AVDR = 0.229
MSRL-Net: An Automatic Segmentation of Intracranial Hemorrhage for CT Images Based on the U-Net Framework	2023	Wang et al.	https://www.mdpi.com/2076-3417/13/21/11781	Proposes MSRL-Net for hemorrhage segmentation.	**PRIVATE DATASET**	82 scans	1. Skull removal<br>2. Pixel conversion<br>3. Data augmentation	Proposes MSRL-Net architecture, which is based on the U-Net structure with several modifications, including the implementation of MSPooling, which is a combination of MaxPool and SoftPool, instead of max-pooling for multi-scale feature extraction, and the integration of Multi-Rate Hybrid Dilated Convolution (MRHDC) structure for mitigating the influence of image resolution reduction.	Dice; Precision; Sensitivity; Jaccard	Dice = 0.717; Precision = 0.712; Sensitivity = 0.779; Jaccard = 0.596
Voxels Intersecting Along Orthogonal Levels Attention U-Net for Intracerebral Haemorrhage Segmentation in Head CT	2023	Liu et al.	https://ieeexplore-ieee-org.ezproxy-b.deakin.edu.au/document/10230843	Proposes a novel and flexible attention based U-Net architecture for ICH segmentation	**INSTANCE 2022 Challenge Dataset**	200 scans	1. Windowing	Proposes viola-Unet architecture, which is an asymmetric encoder-decoder U-Net with 7-depth layers. The key component is the "Viola attention module". This module first uses an adaptive average pooling (AdaAvgPool) module to squeeze the input feature volume into three latent representation spaces along each orthogonal axis (height, width, and depth). Then, customized dense dilated convolutions merging (DDCM) networks fuse cross-channel and non-local contextual information on each orthogonal direction with adaptive kernel sizes, dilated ratios, and strides. Finally, the Viola unit constructs an attention volume based on the fused and reshaped latent representations, as described by mathematical equations involving Sigmoid, group normalization with Tanh, and ReLU activation functions, as well as tensor product and element-wise multiplication. The viola-Unet architecture also incorporates skip connections and can have additional output heads for deep supervision.	Dice; Hausdorff Distance (HD); Relative Absolute Volume Difference (RVD); Normalized Surface Dice (NSD)	Dice = 0.7951 ± 0.171; HD = 24.038 ± 29.236; NSD = 0.5693 ± 0.125; TVD = 0.1941 ± 0.179
A Deep Learning-Based Automatic Segmentation and 3D Visualization Technique for Intracranial Hemorrhage Detection Using Computed Tomography Images	2023	Khan et al.	https://www.mdpi.com/2075-4418/13/15/2537	Develops a machine-learning algorithm that can detect intracranial hemorrhage based on plain CT images	**PhysioNet**	82 scans	1. Windowing<br>2. Skull stripping<br>3. Image inversion<br>4. Data augmentation	Expores various deep learning architectures, including U-Net, U-Net++, and FPN (Feature Pyramid Network). These architectures were implemented with multiple pre-trained encoders (DenseNet121, DenseNet161, DenseNet201, ResNet18, ResNet152, InceptionV4, InceptionV2-ResNet) that were initially trained on the large-scale ImageNet dataset, utilizing the principle of transfer learning. They found that the Densenet201_U-Net model achieved the best performance.	Dice; IoU; Accuracy	Dice = 85.757%; IoU = 84.3%; Accuracy = 99.91%
Bayesian Deep Learning Outperforms Clinical Trial Estimators of Intracerebral and Intraventricular Hemorrhage Volume	2022	Sharrock et al.	https://pmc-ncbi-nlm-nih-gov.ezproxy-b.deakin.edu.au/articles/PMC9474710/#ABS1	Uses Bayesian DL for ICH and IVH volume estimation	**PRIVATE DATASET:**<br><br>phase II (MISTIE II and CLEAR IVH) and phase III (MISTIE III and CLEAR III) clinical trials	1185 subjects	1. Convert to NiFTi	Develops a custom DNN called Intracerebral intraVentricular Network (IV-Net) based on the V-Net architecture for 3D volumetric medical image segmentation. IV-Net incorporated dilated convolutions for contextual propagation and multiclass attention-gating to focus learning on important context. Monte-Carlo Bernoulli dropout sampling was used to approximate uncertainty. Dropout was added to each layer, and 10 stochastic forward passes were performed to obtain a distribution of model parameters.	Dice; Volume Correlation; RMSE; Absolute Volume Difference	**MISTIE II**<br>Dice = 0.940; Volume Correlation = 0.995; RMSE = 2.22; Absolute Volume Difference = 0.655<br><br>**CLEAR IVH**<br>Dice = 0.905; Volume Correlation = 0.995; RMSE = 4.93; Absolute Volume Difference = 2.74
Deep learning-based computed tomography image segmentation and volume measurement of intracerebral hemorrhage	2022	Peng et al.	https://pmc-ncbi-nlm-nih-gov.ezproxy-f.deakin.edu.au/articles/PMC9575984/	Enhancing the accuracy and practicability of CT image segmentation and volume measurement of ICH by using deep learning technology	**PRIVATE DATASET:**<br>Patients from the hospital of Army Medical University (Southwest Hospital)	1027 scans	1. Resize (512x512)	Proposes AttFocusNet, which is a 2D segmentation network based on the U-Net architecture and incorporates two key components: the attention gate (AG) mechanism and a focus structure. The attention gate (AG), adapted for 2D implementation, is used in the encoder stage to suppress irrelevant features and focus on task-related ones during model learning. It combines features from the encoder's skip connections and the up-sampled features from the decoder. The focus structure, adopted from YOLO-V5, is introduced in the encoder to integrate image information into the channel space without initial pooling, aiming to preserve the integrity of ICH features, especially at small lesion points.	Dice; IoU; Sensitivity; PPV; HD95	Dice = 0.908; IoU = 0.874; Sensitivity = 0.913; PPV = 0.957; HD95 = 5.960mm
A symmetric prior knowledge based deep learning model for intracerebral hemorrhage lesion segmentation	2022	Nijiati et al.	https://pmc-ncbi-nlm-nih-gov.ezproxy-f.deakin.edu.au/articles/PMC9727183/	Develops an accurate and efficient deep learning model for the segmentation and diagnosis of ICH	**PRIVATE DATASET:**<br>Patients who were diagnosed with ICH from January 2019 to April 2022 at the First People's Hospital of Kashi Prefecture	1,157 patients	1. Windowing<br>2. Resize (512x512)<br>3. Data augmentation	Proposes Sym-TransNet, a deep learning model built upon the U-Net architecture. It features an encoding path to extract high-level semantic information and a decoding path to restore the image resolution, connected by a novel symmetric Transformer. The symmetric Transformer takes the feature map from the encoder and its horizontally flipped version as input, embedding them into sequence features. It then uses multi-head self-attention (MHSA), where the query and value are derived from the original feature, and the key from the flipped feature, to model long-distance symmetric relationships. This is followed by a multi-layer perceptron (MLP) to refine the features before being fed into the decoding path with skip connections.	Dice; Accuracy; Sensitivity; Specificity	ICH_Dice = 0.716; IPH_Dice = 0.784; IVH_Dice = 0.680; EDH_Dice = 0.359; SDH_Dice = 0.534; SAH_Dice = 0.337; Accuracy = 91.25%; Sensitivity = 98.5%; Specificity = 84.00%
Automatic hemorrhage segmentation on head CT scan for traumatic brain injury using 3D deep learning model	2022	Inkeaw et al.	https://www.sciencedirect.com/science/article/pii/S0010482522003225	Develops a novel automatic method for segmenting three subtypes of intracranial hemorrhage (ICH) - subdural hemorrhage (SDH), epidural hemorrhage (EDH), and intraparenchymal hemorrhage (IPH) - on head CT scans using a 3D deep learning model	**PRIVATE DATASET:**<br>Maharaj Nakorn Chiang Mai Hospital, Thailand, from January 1, 2014, to December 31, 2014.	153 patients	1. Windowing	Employs a three-dimensional CNN based on the DeepMedic model architecture to segment three subtypes of ICH (EDH, SDH, and IPH). The model includes four parallel pathways processing the input at different resolutions and two fully connected layers.	Dice; Sensitivity; Specificity	Dice = 0.59; Sensitivity = 0.70; Specificity = 0.99
Deep Network for the Automatic Segmentation and Quantification of Intracranial Hemorrhage on CT	2021	Xu et al.	https://www.frontiersin.org/journals/neuroscience/articles/10.3389/fnins.2020.541817/full	Develops a framework based on a modified Dense U-Net architecture for the automatic segmentation and quantification of three types of intracranial hemorrhage.	**PRIVATE DATASET:**<br>Beijing Tiantan Hospital Affiliated to Capital Medical University (Hospital A)<br><br>QingPu Branch of Zhongshan Hospital Affiliated to Fudan University (Hospital B)	3,000 scans (training + validation); 211 scans (testing)<br><br>86 scans (external testing)	N/A	Utilizes a deep learning model based on the Dense U-Net architecture. This network combines the dense connectivity of DenseNet encoders with the U-Net structure for segmentation of ICH in CT scans.	Dice	ICH_Dice = 0.90 ± 0.06; EDH_Dice = 0.88 ± 0.12; SDH_Dice = 0.82 ± 0.16
